# Cluster randomised controlled trial of double-dose azithromycin mass drug administration, facial cleanliness and fly control measures for trachoma control in Oromia, Ethiopia: the stronger SAFE trial protocol

**DOI:** 10.1136/bmjopen-2024-084478

**Published:** 2024-12-23

**Authors:** Anna R Last, Oumer Shafi Abdurahman, Katie Greenland, Ailie Robinson, Claire Collin, Alexandra Czerniewska, Edao Sinba Etu, Bart Versteeg, Robert Butcher, Meseret Guye, Demitu Legesse, Kedir Temam Nuri, Gemeda Shuka, Munira Haji Mohammed Yousuf, Gaddisa Dheressa, Gebeyehu Dumessa, Melesse Akalu, Mesfin Tadesse, Dereje Adugna Kumsa, Fikre Seife Gebretsadik, Aida Abashawl, Esmael Habtamu, Virginia Sarah, Wondu Alemayehu, Anthony Solomon, Helen Anne Weiss, James G Logan, David Macleod, Matthew J Burton

**Affiliations:** 1 Clinical Research Department, London School of Hygiene & Tropical Medicine, London, UK; 2 The Fred Hollows Foundation, Addis Ababa, Ethiopia; 3 Department for Disease Control, London School of Hygiene and Tropical Medicine, London, UK; 4 Department of Infection Biology, London School of Hygiene & Tropical Medicine, London, UK; 5 Department for Disease Control, London School of Hygiene & Tropical Medicine, London, UK; 6 Department of Disease Control, London School of Hygiene & Tropical Medicine, London, UK; 7 Berhan Public Health Consultancy, Addis Ababa, Ethiopia; 8 Knowledge Institute of the Dutch Association of Medical Specialists, Utrecht, The Netherlands; 9 Clinical Research Department, London School of Hygiene and Tropical Medicine, London, UK; 10 Oromia Regional Health Bureau, Addis Ababa, Ethiopia; 11 Ethiopia Ministry of Science and Technology, Addis Ababa, Ethiopia; 12 London School of Hygiene & Tropical Medicine, London, UK; 13 The Fred Hollows Foundation UK, London, UK; 14 Global Neglected Tropical Diseases Program, World Health Organization, Geneva, Switzerland; 15 Epidemiology and Population Health, London School of Hygiene and Tropical Medicine, London, UK; 16 Arctech Innovation Ltd, Dagenham, UK; 17 Epidemiology and Population Health, London School of Hygiene & Tropical Medicine, London, UK; 18 Moorfields Eye Hospital NHS Foundation Trust and UCL Institute of Ophthalmology, London, UK

**Keywords:** Epidemiology, Entomology, PUBLIC HEALTH, OPHTHALMOLOGY, Mass Drug Administration, Clinical Trial

## Abstract

**Introduction:**

Trachoma is caused by the bacterium *Chlamydia trachomatis* (*Ct*). The WHO recommends the SAFE strategy for trachoma elimination: Surgery for trichiasis, Antibiotics, Facial cleanliness and Environmental improvement. Multiple rounds of SAFE implementation have proven insufficient to eliminate trachoma in Ethiopia, where over 50% of the global trachoma burden remains. More effective antibiotic treatment schedules and transmission-suppressing approaches are needed. The aim of stronger SAFE is to evaluate the impact of a novel package of interventions to strengthen the A, F and E of SAFE on the prevalence of ocular *Ct* and trachoma in Oromia, Ethiopia.

**Methods and analysis:**

68 clusters were randomised in a 1:1:1:1 ratio to one of (1) standard A/standard F&E (standard SAFE), (2) standard A/enhanced F&E, (3) enhanced A/standard F&E or (4) enhanced A/enhanced F&E (stronger SAFE). Enhanced A includes two height-based doses of oral azithromycin (equivalent to 20 mg/kg) given as single doses 2 weeks apart, as mass drug administration, annually. Enhanced F&E includes fly control measures (permethrin-treated headwear and odour-baited traps) and face-washing hygiene behaviour change implemented at household level in selected communities. The interventions will be implemented and reinforced over 3 years.

The primary outcome is the prevalence of ocular *Ct* by quantitative PCR in children aged 1–9 years at 36 months. A key secondary outcome is the prevalence of active (inflammatory) trachoma in the same children, assessed by validated trachoma graders and conjunctival photography. Laboratory technicians and photo-graders are masked to treatment allocation. Other important secondary analyses include process evaluations, assessment of behaviour change, fly indicators, adherence and coverage of interventions and a cost analysis.

**Ethics and dissemination:**

Study protocols have been approved by the National Research Ethics Review Committee of the Ethiopian Ministry of Science and Higher Education and the London School of Hygiene & Tropical Medicine Ethics Committee. An independent data safety and monitoring board oversees the trial. Results will be disseminated through peer-reviewed publications, presentations and reports.

**Trial registration number:**

ISRCTN40760473.

**STRENGTHS AND LIMITATIONS OF THIS STUDY**

- This is the only trial to test the proposed strengthened combined intervention package of double-dose azithromycin, targeted fly control and hygiene behaviour change, compared with standard trachoma elimination approaches in Ethiopia.
- Interventions have been codeveloped with communities and national/regional trachoma programmes, and pilot-tested for efficacy, feasibility and acceptability, with scalability in mind.
- The primary outcome is the masked detection of ocular *Chlamydia trachomatis* infection using quantitative PCR, which is more objective, sensitive and specific than clinical indicators and a better indication of whether transmission has been interrupted.
- Interventions were designed with and for communities in West Arsi, Oromia, which may reduce their generalisability to populations elsewhere.
- Due to the geographical proximity of some clusters, there is a potential risk of contamination.

## Introduction

 Trachoma is caused by conjunctival infection with the bacterium *Chlamydia trachomatis* (Ct). It is the most common and preventable infectious cause of blindness globally. The WHO recommends the SAFE Strategy for trachoma control: Surgery for trichiasis, Antibiotics for infection, Facial cleanliness and Environmental improvement.^1^ However, multiple rounds of SAFE implementation, predominantly focusing on the ‘A’ component delivered as a single round of mass drug administration (MDA), have proven insufficient to eliminate trachoma in Ethiopia, where over 50% of the global trachoma burden now exists.^2^ Strengthened, more effective antibiotic treatment schedules and transmission-suppressing approaches are needed.^3^,^7^ Facial cleanliness and environmental improvement (the ‘F&E’ components of SAFE) are thought to help suppress transmission^8^ but have limited evidence for efficacy, mostly from observational studies,^9 10^ with few randomised trials to date.^11^,^17^ Not all of these studies included an infection endpoint, and none examined behaviour change outcomes. We have previously modelled the efficacy of a package including double-dose azithromycin, facial cleanliness promotion and fly control measures compared with standard SAFE implementation, supporting their inclusion in the stronger SAFE trial.^7^

The stronger SAFE intervention is composed of ‘Enhanced Antibiotic MDA’ (two single doses of oral azithromycin 2 weeks apart), informed by a within-community mathematical model of trachoma transmission which predicted that this regime would control infection more successfully than a single dose or 6-monthly doses,^7^ combined with targeted transmission-reducing hygiene behaviour change and fly control strategies (‘enhanced F&E’), also informed by this model and previous studies,^718^,^22^ and codeveloped with stakeholders and local communities. In addition to the primary endpoint of ocular *Ct* detection by quantitative PCR (qPCR), this trial has key behaviour change, process and entomological outcomes that will significantly add to the F&E evidence base. Our aim is to provide robust evidence for decision-making for trachoma programme managers and policy-makers to accelerate progress towards global trachoma elimination.

## Methods and analysis

### Study aim

To test the hypothesis that stronger SAFE can more effectively control trachoma, determined by measuring the presence of ocular *Ct* by qPCR, than current standard approaches, in a trachoma-endemic area of Oromia Region, Ethiopia.

### Study design

**Figure 1:**
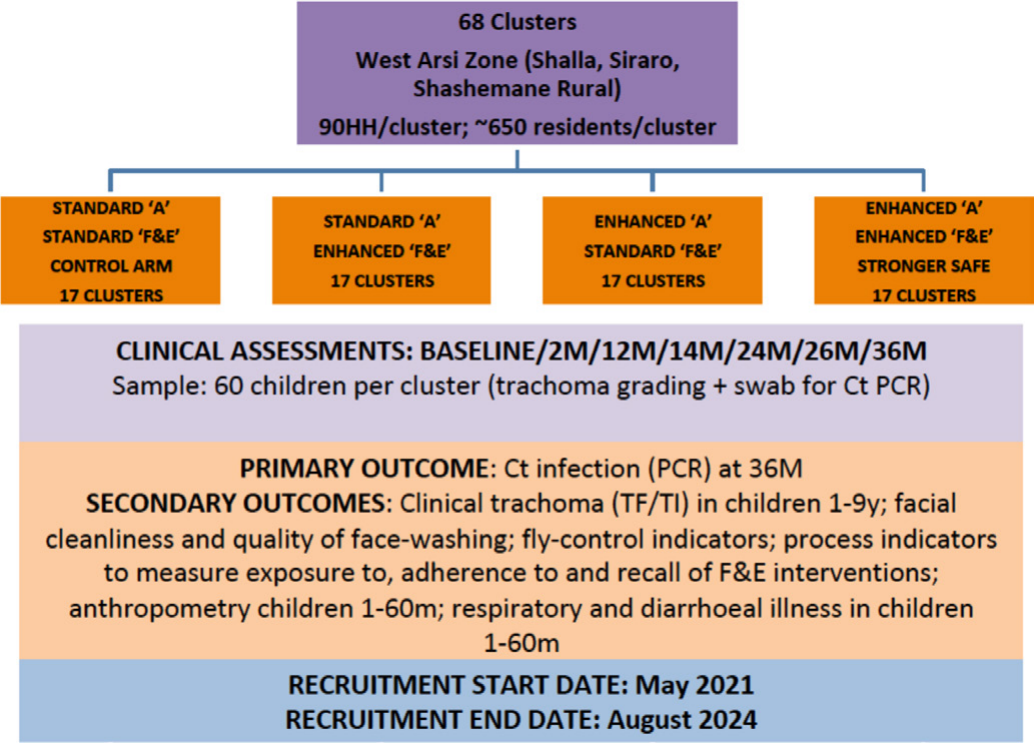
Stronger SAFE trial schematic. Schematic showing 68 clusters randomised to 1 of 4 arms in a 1:1:1:1 ratio in a parallel-group cluster randomised controlled trial. Ct, *Chlamydia trachomati*; HH, households; TF, trachomatous inflammation-follicular; TI, trachomatous inflammation-inflammatory.

This study is a parallel-group cluster randomised controlled trial. 68 clusters have been randomised to one of four arms in a 1:1:1:1 ratio (figure 1):

1. Standard antibiotic/standard F&E (standard SAFE, control arm).
2. Standard antibiotic/enhanced F&E.
3. Enhanced antibiotic/standard F&E.
4. Enhanced antibiotic/enhanced F&E (stronger SAFE, intervention arm).

To minimise contamination, clusters were configured using a ‘fried egg’ design, such that the intervention or control conditions are administered throughout the cluster (‘yolk’ and ‘white’) but participants selected to measure study outcomes are only selected from the centre of each cluster (‘yolk’).^23^ This design ensures that the outcome evaluation sample is completely surrounded by a buffer zone under the same intervention or control conditions.

### Study setting

The study is being conducted in rural communities in Shashemane Rural, Shalla and Siraro districts (*woreda*) in West Arsi, in the Oromia Region of Ethiopia. This is an arid trachoma-endemic region predominantly inhabited by Afan Oromo people. Kebeles are administrative units within each *woreda* comprising approximately 5000 people. Kebeles are subdivided into three *zones*, which are further divided into garees. Each garee includes approximately 30 households. Kebeles with a high prevalence of trachomatous inflammation-follicular (TF) in children aged 1–9 years, identified by the Global Trachoma Mapping Project and subsequent recent trachoma screening surveys, were purposively selected. MDA with azithromycin has been given annually in this region since 2016. Prior to the trial intervention MDA had not been given since 2018, in part due to restrictions imposed during the COVID-19 pandemic in 2020. In the trial, each cluster includes 2–3 garees (approximately 90 households). The ‘yolk’ includes 50 households in the centre of the cluster, from which the outcome population is selected.

### Trial interventions

#### Antibiotic intervention (A)

Within kebeles, zones were allocated to an antibiotic intervention arm. Each *zone* includes two trial clusters.

Azithromycin is donated to the national trachoma control programme by the manufacturer (Pfizer) through the International Trachoma Initiative. In antibiotic MDA, individuals aged ≥6 months will be offered height-based treatment with azithromycin, delivering approximately 20 mg/kg to a maximum dose of 1 g. Children below the age of 6 months will be given tetracycline eye ointment instead of azithromycin, in accordance with national policy for trachoma elimination.

##### Standard antibiotic (arms 1 and 2)

17 zones (34 clusters, 2 clusters per zone (see randomisation and assignment of interventions below)) were randomised to receive standard annual single-dose antibiotic MDA for 3 years.

##### Enhanced antibiotic (arms 3 and 4)

17 zones (34 clusters, 2 clusters per zone) were randomised to receive annual enhanced antibiotic, where an additional single dose of oral azithromycin (20 mg/kg up to a maximum dose of 1 g) will be given 2 weeks after the initial programmatic MDA (delivered as described above) to all individuals above the age of 2 years for three annual rounds in accordance with Ethiopian national regulatory body requirements.

MDA in all arms is directly observed and recorded. MDA is delivered by the regional trachoma elimination programme. Our study team is embedded within the distribution team to support data collection during distribution.

### F&E interventions (F&E)

#### Standard F&E (arms 1 and 3)

Programmatic promotion of latrine construction and facial cleanliness through health-promotion messaging and collaboration with the WASH (Water, Sanitation, Hygiene) sector to advocate for improved water supply in line with the current WHO recommendations are being delivered to 34 randomly selected clusters.

#### Enhanced F&E (arms 2 and 4)

Fly-control interventions and a hygiene behaviour change intervention are being delivered to 34 clusters randomised to receive the enhanced F&E intervention in addition to standard programmatic F&E.

### Fly control interventions

The fly-control interventions consist of a ‘push-pull’ strategy, involving permethrin-treated headwear (PTH) and odour-baited traps (OBT) distributed to entire clusters allocated to arms 2 and 4.

#### Permethrin-treated headwear

**Figure 2:**
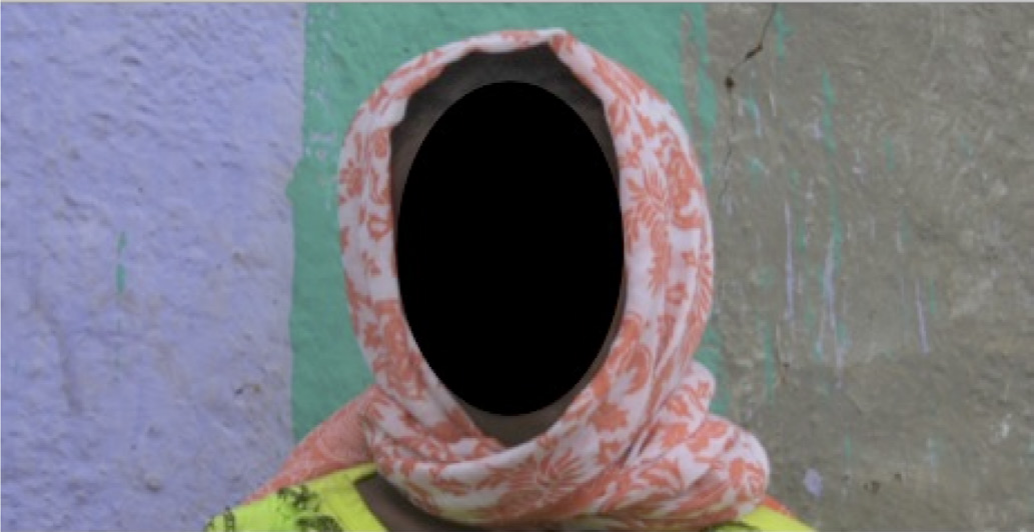
Repellent permethrin-treated headwear (PTH). A participant wearing a permethrin-treated scarf. The selected PTH is a commercial product made from 100% polyester, factory-treated with proprietary permethrin ((m-Phenoxybenzyl)-cis,trans-3-(2,2-dichlorovinyl)−2,2-dimethylcyclopropanecarboxylate, CAS number 52645-53-1) at a concentration of less than 1.25 g/m^2^ fabric (this is equivalent to 0.125 mg/cm^2^, the concentration that the US-Environmental Protection Agency considers to be safe for all ages) (InsectShield). Photograph with permission from Robinson *et al*.^21^

The ‘push’ is a repellent strategy using insecticide (permethrin)-treated headscarves or caps (InsectShield), worn by children aged 2–9 years to provide personal protection from eye-seeking flies, and potentially affecting fly survival.^21^ The choice of active ingredients and clothing items was based on our previous studies, which included work on acceptability and adherence.^21^ The selected PTH is a commercial product made from 100% polyester, factory-treated with proprietary permethrin (3-phenoxyphenyl-methyl 3-(2,2-dichloroethenyl)−2,2-dimethylcyclopropane-1-carboxylate, CAS number 52645-53-1) at a weight ratio of 0.52% w/w of the finished product. This concentration is less than 0.125 mg/cm^2^, the concentration that the US-Environmental Protection Agency considers to be safe for all ages) (InsectShield) (figure 2 photo with permission). The repellency of this product is US-EPA registered for durability of over 70 launderings, the perceived lifetime of the garment. Communities were involved in decision-making towards the final selection of PTH, to maximise engagement and uptake in the trial. The use of PTH is promoted in intervention clusters via health promotion campaigns including oral information and instructions. New PTH is distributed to participant households every 4 months to maximise durability.

#### Odour-baited traps

**Figure 3:**
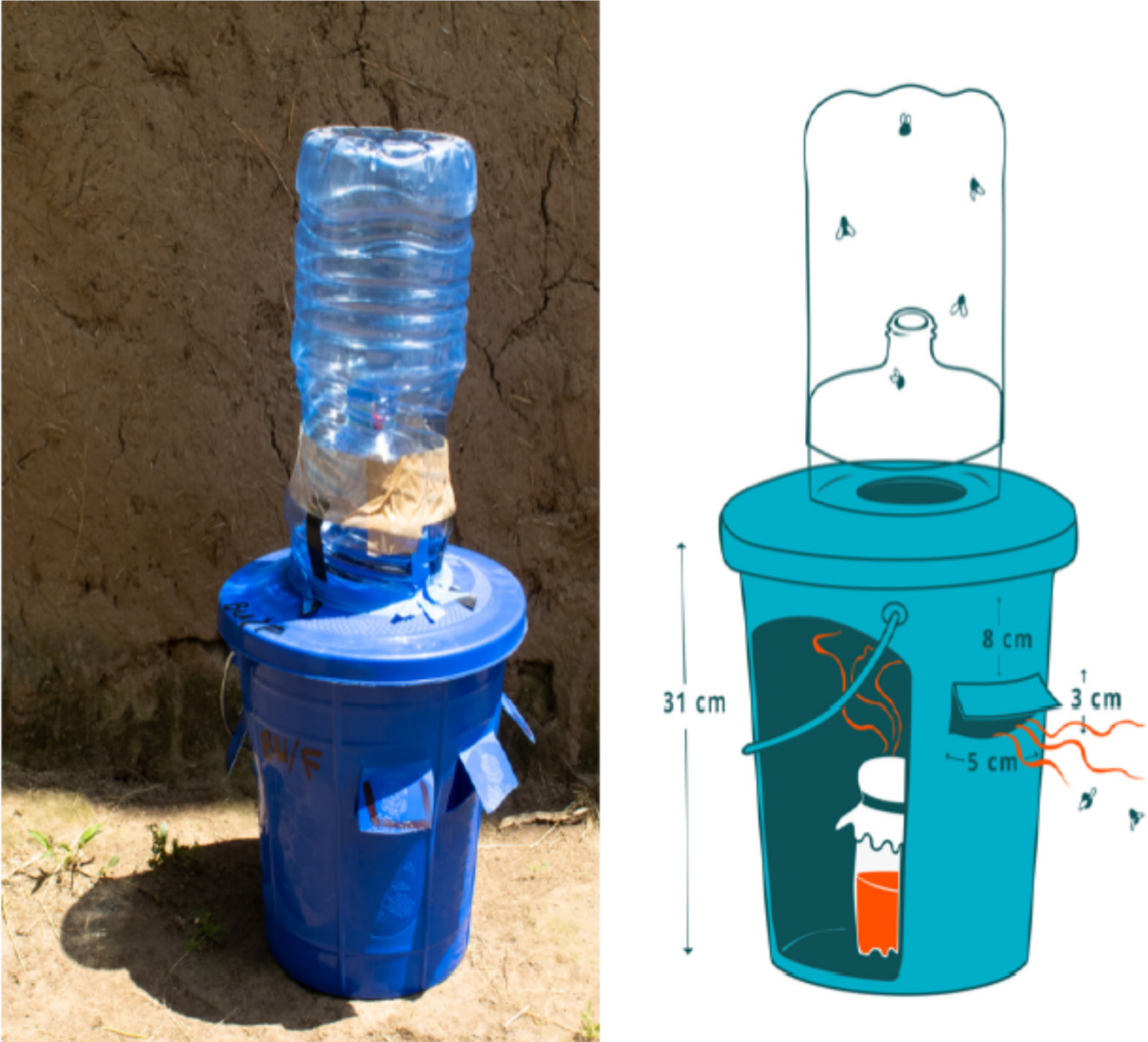
Odour-baited trap (OBT) deployed to capture *Musca sorbens* in stronger SAFE. A homemade OBT trap, designed using locally available materials, constructed from a 5 L water bottle attached to a plastic bucket (cut to specification), baited with a commercially available lure (The Buzz).

The ‘pull’ involves OBT deployed at the household level to reduce local fly population density. Commercially available and homemade lures and traps were tested initially to select the most efficacious OBT for use in the trial.^20^ The final selected OBT is a homemade trap designed using locally available materials, constructed from a 5 L water bottle trap attached to a plastic bucket (cut to specification), baited with a commercially available lure (The Buzz) made up within a 250 mL water bottle, covered with mesh (figure 3). OBT is distributed to recipient households, with instruction on their construction, siting and maintenance. The Buzz lure is active for 4 weeks and is, therefore, distributed monthly during the intervention period. Lure distribution is accompanied by spot-checks of user compliance with protocol and further instruction/guidance for use. Participants are encouraged/trained to contact the field team should they require lure more frequently.

#### Fidelity of fly control interventions

Sustained use of PTH is important and has been emphasised during recruitment. Following the first roll-out meeting, monthly visits to the households during the hot/dry season (December to February inclusive), by two health volunteers (HVs) per cluster supports participation and protocol adherence for both ‘push’ and ‘pull’ interventions. Additional reinforcement and replacement lure distribution is conducted monthly throughout the year by the HV. This intensive support will improve participation and protocol adherence for both ‘push’ and ‘pull’ interventions. To further assess trial adherence and embed quality control into the trial, a limited number of unannounced visits (spot checks) by the study team will take place in each cluster, to allow an opportunity for problems relating to ‘push-pull’ fly control interventions to be identified and addressed. Participants will be informed about the possibility of random spot checks at recruitment and during consent.

The physical and chemical durability of fly control products is monitored in a random selection of households every 6–8 weeks to measure attrition (loss of items), biological efficacy, chemical content (amount of permethrin) and physical integrity (physical condition). These visits are also used to understand participants’ adherence to and acceptability of the intervention.

### Hygiene behaviour change intervention

#### Formative research and intervention development

**Figure 4:**
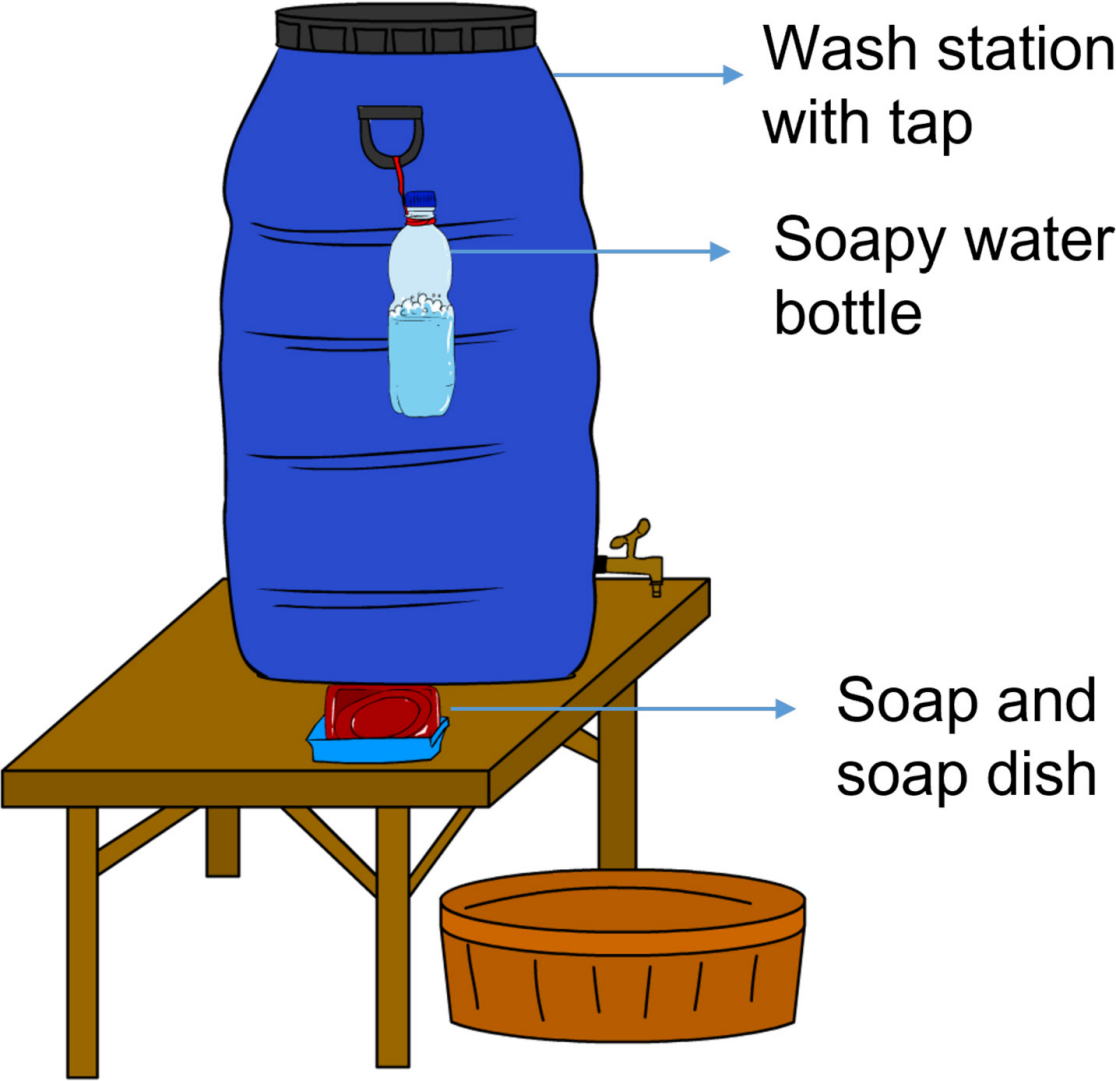
Household WASH (Water, Sanitation, Hygiene) station employed in stronger SAFE. A simple wash station with a faucet, bar of soap and a soap dish were distributed to all households in the yolk. All residents of the cluster were invited to the community event and received components to build the wash station, and instructions for washing with soap (or soapy water). Reinforcement of the target behaviour and troubleshooting of barriers affecting WASH station function and use were conducted through family forums.

The hygiene behaviour change package is based on a theory of change that multiple behavioural factors will influence specific facial cleanliness practices using behaviour change techniques delivered through multiple channels. The main behaviour change goal is increased frequency and effectiveness (soap use) of face washing of preschool aged children by their primary carer. Specifically, faces (and hands) of the whole family (particularly preschool children) should be thoroughly washed with soap three times a day: Morning after waking, before lunch and before the evening meal. The ‘Faces of Dignity’ intervention package seeks to increase the perceived value of face washing by amplifying motivational drivers associated with face washing, address perceptions about water availability and other barriers limiting face washing practices, provide cues in the environment to remind or trigger face washing and lower the transaction costs associated with face washing by encouraging the provision and use of convenient soap (soapy water) and water utensils (household wash station) (figure 4). More details about the content and delivery of the hygiene behaviour change intervention can be found in online supplemental file 1.

**Figure 5:**
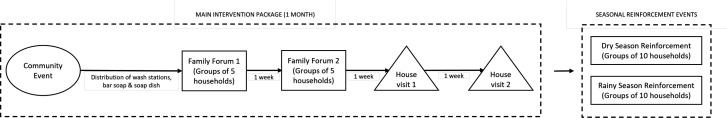
Hygiene behaviour change intervention developed for stronger SAFE. The hygiene behaviour change intervention was codeveloped with the community and is designed to be low-cost and sustainable, delivered by a team of implementers and health volunteers. The intervention comprises five main contact points over a month, with subsequent activities in the dry and rainy seasons each year.

The hygiene behaviour change intervention was codeveloped with the community and is designed to be low-cost and sustainable, delivered by a team of implementers and HVs. The intervention comprises five main contact points over a month, with subsequent activities in the dry and rainy seasons each year (figure 5). All residents of the cluster were invited to the community event and received components to build the wash station. The intensive intervention is being delivered to the cluster ‘yolk’.

#### Community event

All households in the cluster were invited to the ‘Faces of Dignity’ campaign: A community event intended to raise awareness and credibility of the campaign among the community, create buy-in (especially from men), introduce the target behaviour and begin to build washing-related knowledge and motivation. Behaviour change techniques include testimonials from influential leaders, public pledging and cultural activities (a drama involving traditional Chaltu puppetry). A simple wash station with a faucet, bar of soap and a soap dish was distributed to all households (figure 4).

#### Family forums

Following the community event, two family forums are held a week apart with groups of five households at a time in the cluster ‘yolk’ to reinforce the target behaviour, modify perceptions and troubleshoot barriers affecting wash station function and (continued) practice of face washing with soap.

#### Household visits

Additionally, two individual household visits are made to ‘yolk’ households to provide ongoing support to motivate families to wash faces with soap three times a day throughout the year and to ensure families feel equipped to maintain their wash stations.

#### Seasonal reinforcement events

Seasonal reinforcement events are conducted in the dry and rainy seasons to reinforce messaging and allow for problem solving of challenges faced by communities in adhering to the intervention, including overcoming specific seasonal barriers. These events involved gender-separate discussions with groups of 10 households at a time.

#### Fidelity of hygiene behaviour change intervention

All implementers received training in how to deliver the package, are covertly monitored at intervals throughout intervention delivery and receive subsequent refresher training to maximise fidelity. Enhanced efforts are made to reach socially excluded or vulnerable households throughout.

For all ‘enhanced F&E’ interventions, community HVs from within the intervention communities communicate with the study team on matters such as broken or lost items that need replacing during the study. This is monitored through reinforcement and follow-up.

### Randomisation and assignment of interventions

This study involves 68 clusters, with 17 allocated to each trial arm. MDA takes place at the zone level, to ensure an adequate treatment buffer zone between clusters, however, the household-level F&E intervention was anticipated to be too intensive to deliver to such a large area within the context of the trial, so a two-stage approach to randomisation was taken. First, 34 zones (within kebeles) were selected based on having a high prevalence of TF in children aged 1–9 years during screening surveys. Within each zone two clusters were identified; a cluster comprises 2–3 garees (approximately 90 households collectively). The 34 zones were randomly assigned to receive either standard or enhanced antibiotic (with 17 zones receiving each option), and randomisation was restricted to ensure balance between arms in terms of TF prevalence and geographical location. The two clusters within each zone were then randomised to either standard or enhanced F&E. The randomisation sequence was generated by the trial statistician using Stata V.17.

Additionally, clusters receiving enhanced antibiotic will have a buffer zone comprising the zone of the kebele around the two clusters in that arm. Standard SAFE (including standard antibiotic) will be implemented by the national trachoma elimination programme in all settlements in surrounding buffer zones with the additional aim of minimising spillover effects (online supplemental file 2).

### Census and baseline surveys

Following obtaining cluster and household-level consent, trained field workers conducted a baseline door-to-door census to enumerate all individuals from all households within each cluster (‘yolk’ and ‘white’). Name, age, gender and residency (permanent or temporary (defined as less than four weeks)) are recorded for each household member. Household head contact details and GPS coordinates are collected for each household. A baseline cluster survey was conducted to record the presence of schools, health facilities and water points used by the community. In each cluster, a baseline household survey to evaluate water and sanitation access and face washing behaviour was conducted in three randomly selected households with at least one child aged 1–6 years. Individuals residing in the household were invited to enrol in the study.

### Participants receiving interventions

All individuals resident within a cluster were screened for eligibility to receive antibiotics (single or double dose according to trial arm allocation). All households within clusters allocated to receive enhanced F&E interventions received an OBT, and children aged 2–9 years received PTH. All ‘yolk’ households within clusters allocated to receive enhanced F&E interventions received a WASH station and were invited to attend the community event and family forums and received household follow-up.

### Inclusion and exclusion criteria

All cluster residents (defined as individuals resident for 4 weeks or more during the trial) are eligible to participate providing the following specific inclusion/exclusion criteria are fulfilled.

### Inclusion criteria: intervention

#### Standard antibiotic

- Adults and children (over the age of 6 months) resident in the cluster.

#### Enhanced antibiotic

- Adults and children (over the age of 6 months for the first dose and 2 years for the second dose) resident in the cluster.

#### Standard F&E

- Adults and children resident in the cluster.

#### Enhanced F&E

- Hygiene behaviour change intervention—adults and children resident in the cluster yolk.
- OBT: Adults and children resident in the cluster.
- PTH: Children aged 2–9 years resident in the cluster.

### Exclusion criteria

- Children under 6 months of age for single-dose azithromycin in all antibiotic arms.
- Children under 2 years of age for the second dose of azithromycin in the enhanced antibiotic arms.
- Illness or incapacity.
- Inability to communicate.
- Inability to provide the samples required for the trial.
- Known hypersensitivity to azithromycin.
- Known hypersensitivity to permethrin.
- Known to be pregnant.
- Confirmed to be taking medications that may cause a serious drug interaction if taken with azithromycin.

Individuals who were excluded from or absent for the intervention would still be eligible to participate in outcome surveys.

### Population for outcome monitoring

At least 60 children aged 1–9 in the ‘yolks’ of each cluster will be examined and have conjunctival swabs taken at 2, 12, 14, 24, 26 and 36 months during the trial to assess clinical outcomes. 324 sentinel households (6 per cluster) are visited every 2 months on a rolling basis to evaluate the efficacy of fly-control interventions using entomological measures, in line with WHO guidance for the evaluation of vector-control interventions.^24 25^ 27 households are randomly selected from the ‘Enhanced F&E’ clusters every 6–8 weeks to evaluate durability of the PTH.^24^ Three households with one or more preschool children aged 1–6 years of age within the ‘yolk’ of each cluster are randomly selected to evaluate behaviour change at 3 months and at the trial endpoint. Proxies of behaviour (presence of soap and water and presence of a functional wash station) were observed and evaluated in all ‘yolk’ households in clusters 3 months after the main intervention delivery in the first year. These observations will be repeated 3 months after the final behaviour reinforcement visit at the trial end.

### Inclusion criteria: outcome monitoring population

- Children aged 1–9 years in the cluster yolk (presence of ocular *Ct* DNA and clinical outcome evaluations).
- Households with at least one child aged 1–6 years (behavioural outcome evaluations).
- Households with at least two children aged 2–9 years (sentinel and PTH durability monitoring).

### Outcome measures

#### Primary outcome

The primary outcome measure is the prevalence of ocular *Ct* detected by qPCR in children aged 1–9 years 12 months after the final MDA round (36 months). The primary comparison is between the Stronger SAFE arm (arm 4) and the standard SAFE arm (arm 1) and will be assessed using a cross-sectional randomly selected sample of at least 60 children aged 1–9 years randomly sampled from each cluster ‘yolk’. *Ct* is detected using a published *Ct*-specific qPCR assay at the Oromia Regional Health Bureau laboratory in Adama, Oromia.^26^

#### Secondary outcomes

Children in the cluster ‘yolk’ are examined for the presence of TF and have conjunctival swabs taken at each monitoring time point (at 2, 12, 14, 24, 26 and 36 months following the first MDA intervention). A key secondary outcome is TF, assessed using the WHO simplified and modified FPC (examination for the presence of follicles, papillary hypertrophy (inflammation) and conjunctival scarring) grading systems,^27 28^ and conjunctival digital photography at each time point. Further detail on the examination procedures for ocular examination and conjunctival swabbing and digital photography using previously published methods.^18^ Other secondary outcomes include facial cleanliness^29^ and quality of face-washing, fly control indicators, process indicators to measure exposure to, adherence to and recall and acceptability of F&E interventions, anthropometry and reported respiratory and diarrhoeal illness in children aged 1–60 months at 36 months and a cost analysis (online supplemental file 3).

### Statistical considerations and data analysis

#### Sample size for primary outcome

17 clusters per arm, with a sample of 60 children/cluster, gives 90% power and 95% confidence to detect an absolute difference of 4% between the control (standard SAFE) (5%) and intervention (stronger SAFE) (1%) arms, assuming a design effect of 2.5 (intracluster correlation less than 0.025, estimated from previous data^30^ and an expected cluster-level *Ct* prevalence range 0%–14%). This would also provide good power for other pairwise comparisons by arm, and at different time points. The sample size considerations for key secondary outcomes are included in online supplemental file 3.

#### Data collection and management

Census, cluster and risk factor surveys, clinical examination and entomological data are collected on mobile devices using custom-designed Open Data Kit forms. Electronic forms are encrypted on submission to a Higher Education and Research Act 2017-compliant secure server at London School of Hygiene & Tropical Medicine (LSHTM). These are uploaded as a relational database and will be downloaded for data cleaning and analysis using restricted access encryption keys. Data can be monitored in real time via the secure server. Paper forms are kept in limited-access locked storage at the trial site office during the trial and scanned for electronic storage thereafter. Data are deidentified, except for consent and MDA record forms, and will be held for a minimum of 7 years, including LSHTM electronic repositories, for which access to deidentified data can be requested. The trial protocol and statistical code will be open access.

#### Data analysis

CONSORT guidelines for analysing and reporting cluster randomised controlled trials will be followed.^31^ A flow chart will show all potentially eligible participants for the primary outcome survey performed at 36 months, and any reasons for exclusion. The number of participants assessed per cluster per arm will be shown, along with number with outcome data. The age and gender characteristics of survey participants will be summarised by arm.

#### Primary analysis

The primary analysis will be a comparison between the control arm (standard SAFE (arm 1)) and the arm receiving both enhanced F&E and enhanced antibiotic (stronger SAFE (Arm 4)). Primary analyses will be by intention to treat (ITT), with clusters/participants analysed according to the arm to which they were randomised with adjustment for baseline *Ct* prevalence.

The intervention effect on *Ct* prevalence will be estimated as a risk difference and 95% CI, estimated using a two-stage approach and adjusted for baseline prevalence of *Ct*. First a logistic regression will be fitted with baseline cluster *Ct* prevalence as an exposure and presence of *Ct* at end line as the outcome. The residuals of this regression will be used in an unpaired t-test to obtain the adjusted risk difference and the associated CI in a two-stage approach recommended by Hayes & Moulton.^23^

#### Secondary analyses

The secondary outcome of TF prevalence will be analysed in exactly the same manner as the primary outcome, except that we will adjust for baseline TF prevalence rather than *Ct*. The effectiveness of each intervention on *Ct* prevalence will be assessed separately, first comparing each trial arm with the control separately (accounting for the matching in comparing the F&E arms, by adjusting for matched pair in stage (1) then using a factorial-style approach (comparing arms 1 and 2 with arms 3 and 4 and comparing arms 1 and 3 with arms 2 and 4). The same two-stage approach employed for the primary comparison (with additional adjustment for the analysis of each intervention for the other in the factorial-style analysis) will be used.

Primary analysis of behavioural outcomes will be conducted on an ITT basis to assess whether the intervention was effective as delivered. Analysis will be adjusted for baseline levels of behaviour. Planned subgroup analysis will be done according to water access, level of education and socioeconomic status. If intervention reach is suboptimal or variable across clusters, per-protocol analyses will be performed to assess whether the intervention succeeded in changing behaviour among those directly exposed to it and subgroup analysis comparing intervention outcomes according to level of intervention exposure will also be performed. The two-stage approach will be followed as above.^23^ The costs of all aspects of the intervention will be tabulated for use in a cost analysis.

### Trial status

The baseline trachoma (and ocular *Ct*) prevalence survey was conducted in March 2021. Three annual rounds of antibiotic MDA were successfully delivered to all clusters in August of 2021, 2022 and 2023. Follow-up surveys have been conducted to date following MDA according to protocol. The behaviour change intervention has been implemented and reinforced according to protocol. The fly-control interventions have been distributed and reinforced according to protocol. InsectShield PTH was distributed every 4 months following the first annual round of MDA in 2021. Further reinforcement events for the ‘enhanced F&E’ interventions were held in 2023–2024. Process evaluations and sentinel surveillance are ongoing. The primary outcome survey was completed in August 2024, with laboratory analysis ongoing (online supplemental file 4).

### Safety monitoring and reporting

Participants are instructed to notify their Health Extension Worker in the case of any potentially intervention-related adverse events, including those linked to antibiotic distributions as well as any thought to be due to hygiene behaviour change interventions or fly-control interventions. Health extension workers report this information to the study team and the trial manager, who will make an assessment according to the approved trial adverse event algorithm (online supplemental file 5). A data safety and monitoring board (DSMB) is responsible for safeguarding the interests of trial participants, assessing the safety and efficacy of the interventions during the trial, and monitoring the overall conduct of the trial (online supplemental file 6). The DSMB meets annually, providing recommendations about whether the trial should be stopped or continued and whether antibiotics should be provided to study communities, and also recommendations relating to the selection, recruitment and retention of participants, data management and quality control.

## Ethics and dissemination

### Ethical approvals

This protocol (V.1.0) adheres to SPIRIT (Standard Protocol Items: Recommendations for Interventional Trials) guidelines^32^ and the trial will be reported according to CONSORT (Consolidated Standards of Reporting Trials) guidelines.^31^ The study adheres to the Declaration of Helsinki^33^ and to Good Clinical Practice.^34^ The trial is a collaboration between the LSHTM (UK), the Federal Ministry of Health (FMOH) (Ethiopia), the Oromia Regional Health Bureau (ORHB) (Ethiopia) and the Fred Hollows Foundation (Ethiopia and International). LSHTM is the trial sponsor (Patricia Henley, Research Governance and Integrity Office). Approval for this trial has been obtained from the LSHTM Ethics Committee (UK) (Reference 17494), the Oromia Regional Health Bureau (BEFO/DDFDHU/1-89/3515), the National Research Ethics Review Committee of the Ethiopian Ministry of Science and Higher Education (MOSHE//RD/141/8082/19) and the Ethiopian Food and Drug Authority (02/25/32/206). Community leaders provide verbal consent before enrolment of the community in the trial. Informed written consent is obtained by the study team after providing written or spoken information (according to literacy) in Afan Oromo. An independent witness signs all consent forms of non-literate participants. Parents/guardians will be asked to consent on behalf of all children below the age of 18 years. Children aged 10–17 years are additionally invited to provide written, informed assent (online supplemental file 7).

### Dissemination

Results will be published in open-access peer-reviewed journals and presented at national and international meetings. Results will also be shared directly with participating communities and discussed with national and international stakeholders with respect to programmatic implementation.

### Patient and Public Inolvement statement

Sensitisation and formative work have been conducted in these communities prior to commencing the trial to promote community engagement.^19^ We are conducting an additional separately funded school-based public engagement activity to enhance school-aged children’s knowledge about trachoma and its prevention to further enhance community engagement in the trial. The stronger SAFE intervention package was codeveloped with communities to ensure that the interventions are low-cost, acceptable and feasible.^19^,^22^ The delivery of the antibiotic MDA is achieved through strong links with regional MDA delivery systems, strengthening the delivery of MDA, reporting of MDA coverage and access, acceptance and adherence by communities. All methods were finalised following a discussion with the local PI (Principal Investigator), the woreda health offices, ORHB and FMOH. Regular feedback is obtained during intervention delivery, from the study team and health extension workers. Trial outcomes, social science, public engagement and qualitative work will be presented to stakeholders and communities.

## supplementary material

10.1136/bmjopen-2024-084478 online supplemental file 1

10.1136/bmjopen-2024-084478 online supplemental file 2

10.1136/bmjopen-2024-084478 online supplemental file 3

10.1136/bmjopen-2024-084478 online supplemental file 4

10.1136/bmjopen-2024-084478 online supplemental file 5

10.1136/bmjopen-2024-084478 online supplemental file 6

10.1136/bmjopen-2024-084478 online supplemental file 7

10.1136/bmjopen-2024-084478 online supplemental file 8

10.1136/bmjopen-2024-084478 online supplemental file 9

10.1136/bmjopen-2024-084478 online supplemental file 10

10.1136/bmjopen-2024-084478 online supplemental file 11

10.1136/bmjopen-2024-084478 online supplemental file 12

10.1136/bmjopen-2024-084478 online supplemental file 13
