# Supplementary material for: Cluster randomised controlled trial of double-dose azithromycin mass drug administration, facial cleanliness and fly control measures for trachoma control in Oromia, Ethiopia: the stronger SAFE trial protocol
Source: BMJ Open. 2024 Dec 23;14(12):e084478. doi: 10.1136/bmjopen-2024-084478 (PMC11751794; doi:10.1136/bmjopen-2024-084478)
Supplement: online supplemental file 1 [file bmjopen-14-12-s001.pdf]

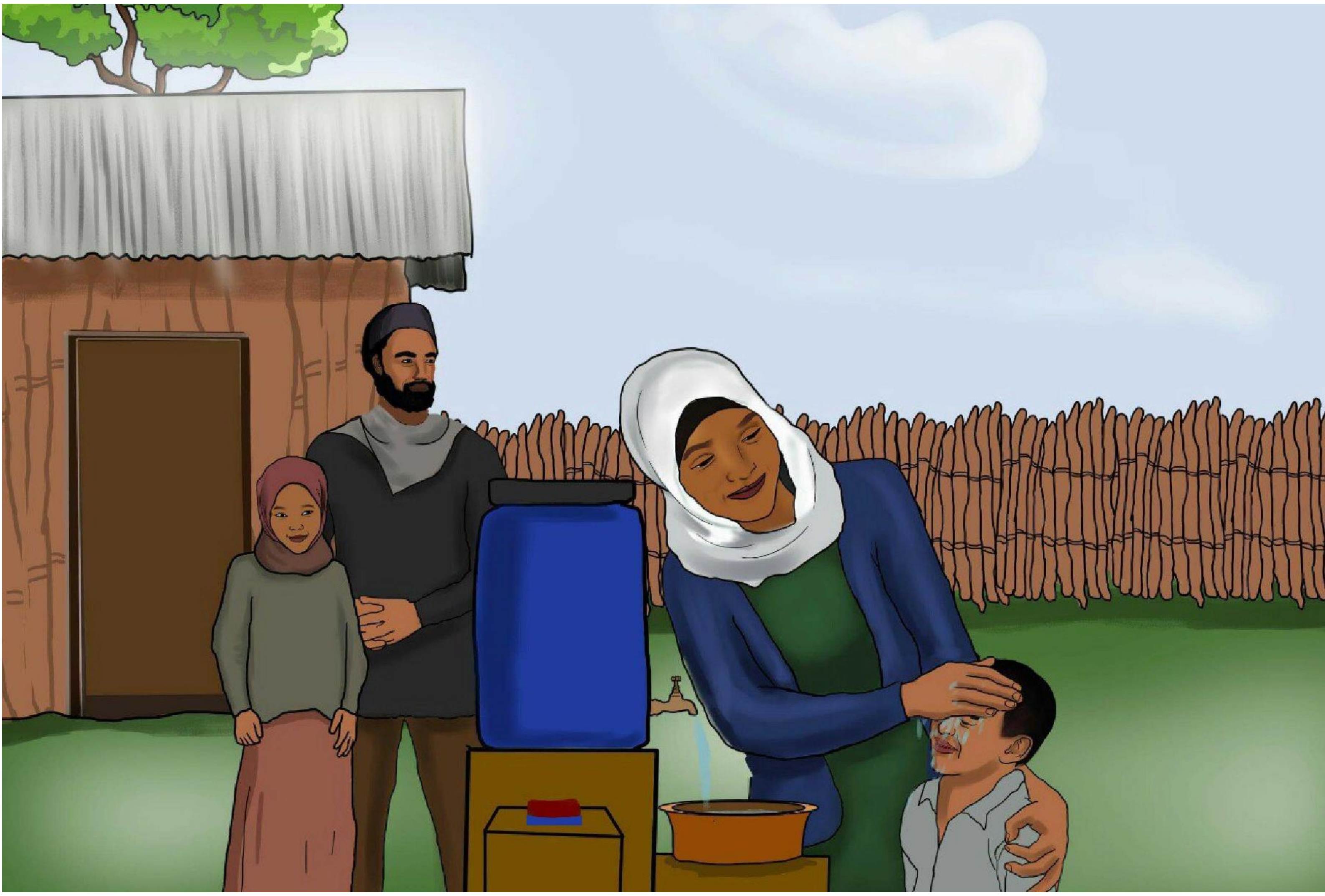

Faces of Dignity Campaign

Intervention Manual – Short Version for Training

Purpose of this Manual

This manual gives an overview of the *Faces of Dignity* intervention delivered during the Wellcome Trust funded Stronger-SAFE trial to improve trachoma elimination efforts in rural Oromia, Ethiopia. The intervention seeks to improve the frequency and quality of face washing with soap among all household members, but particularly pre-school age children (1 to 6 years old). Male and female heads of households with pre-school age children are the key target population of this intervention. The intervention will be delivered to about 2500 households, 1650 of which will receive an intensive version of the intervention.

This manual provides an overview of the content and materials used in the intervention and is not intended as a guide for intervention delivery. A more detailed account of the conduct of each activity can be found in the Implementation Guide for each event.

Intervention Overview

"Intensive Intervention"

The *Faces of Dignity* Campaign is a multi-level intervention comprising a large group event and a series of small group events and individual household visits.

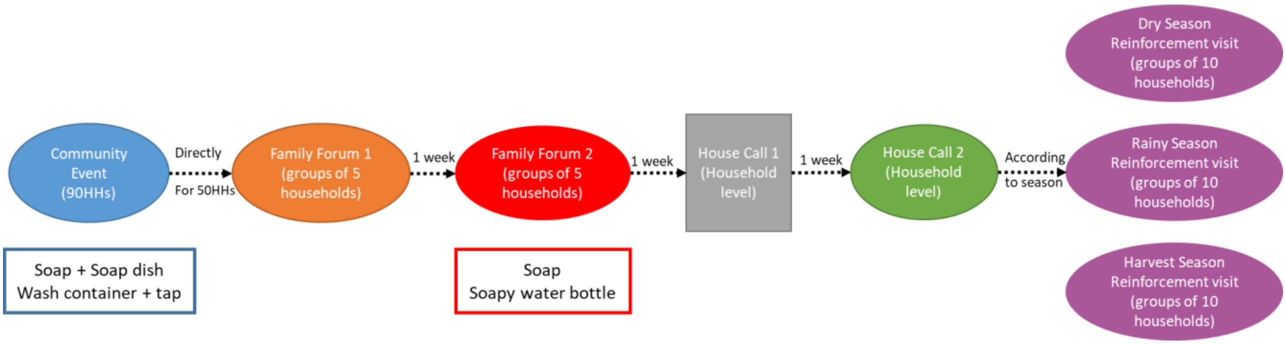

"Basic Intervention"

As the intervention is to be delivered in the context of the Stronger-SAFE trial, and the clusters are quite large, a basic version of the intervention will be delivered to the outer portion of each cluster. This version of the intervention will comprise the Community Event + Provision of a wash station, 2 soaps, a soap dish and a wash station flyer.

If we view a cluster as a fried egg, the structure of the intervention looks like this:

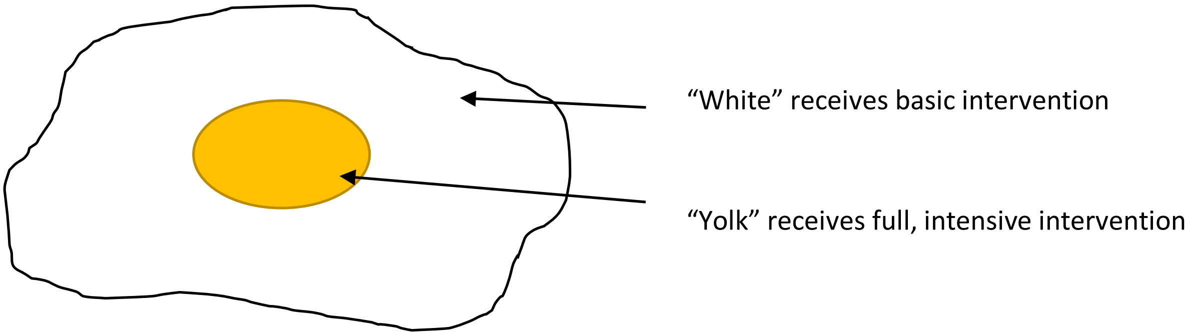

Personnel and Training

The intervention will be delivered in each cluster by a team of facilitators composed of 3 trained Actors, 1 trained Activator, 2 trained Health Volunteers (HVs). Other individuals required to support intervention activities are described in the content for each specific event. Involving both trained Implementers and local HVs intends to ensure intervention fidelity in the trial context whilst also providing a scalable and replicable model to improve intervention reach and uptake in each cluster.

Table. Overview of the *Faces of Dignity* Campaign in each cluster

Event	Facilitator	Purpose	Target Audience	Timing of event
**Community Event** (large-scale event)	3x trained Actors; Involvement of 3x Influential Leader; supporting individuals (HVs, Activators, etc.)	Raise awareness and credibility of the campaign among the community; Create buy-in; Begin to build wash-related knowledge and motivation	All household members living in the intervention cluster ('white' + 'yolk'); community leaders	Kick-starts the intervention in each cluster.
**Family Forum 1** (group event with 5xHHs)	1x trained Activator + 1x trained HV	Build wash-related knowledge, skills, and motivation; Empower households to construct wash stations to aid habit formation	All household members living within the 'yolk' of an intervention cluster	In the week following the community event
**Family Forum 2** (group event with 5xHHs)	1x trained Activator + 1x trained HV	Continue to build wash-related skills and motivation; Overcome early barriers related to wash station construction and use; Emphasis need to wash faces with soap three times a day now that everyone has a functioning wash station	All household members living within the 'yolk' of an intervention cluster	1 week after the previous forum (precise timing is dependent on logistics)
**House Call 1** (individual HH visits)	1x trained HV	Reinforce target messages; Provide encouragement to motivate continued action; Identify and overcome specific barriers to ongoing action, including those related to wash station construction and maintenance	All household members living within the 'yolk' of an intervention cluster	1 week after the previous forum (precise timing is dependent on logistics)
**House Call 2** (individual HH visits)	1x trained Activator	Provide further opportunity to reinforce target messages, develop skills and trouble-shoot specific barriers preventing optimal practise of face washing	All household members living within the 'yolk' of an intervention cluster	2 weeks after Family Forum 2 (precise timing is dependent on logistics)
**Reinforcement Events** (group event with 10xHHs)	2x HVs	Reinforce initial messages; overcome known seasonal barriers	All adults living within the 'yolk' of an intervention cluster	At start of the dry season, start of the rainy season and immediately prior to the main harvest

Considerations associated with COVID-19

The content and delivery of the intervention have been revised to ensure all standard precautionary COVID-19 preventative measures are followed and implemented routinely by all individuals involved in the *Faces of Dignity* Campaign. Safety of both the community and *Faces of Dignity* intervention facilitators is paramount. All intervention activities will take place outside.

The main precautionary measures taken include minimisation of unnecessary contact between any individuals (including between implementers), implementation of physical distancing (at least 2 metre between all individuals from different households) in all settings, use of facemasks in public places and appropriate disposal of those masks, handwashing with water and soap for at least 20 seconds or use of at least a 60% alcohol based hand sanitizer, and regular surface cleaning with detergent.

Detailed procedures to be observed by implementation staff (Actors, Activators and HVs) are described in each event manual. Due to the fast-changing nature of the pandemic, the latest national and local guidance on COVID-19 prevention will be followed at all times.

List of all Collaterals for facilitation

Collateral	Corresponding Event
– Puppet of the *Faces of Dignity* Drama	Community Event | FF1 | FF2 | HC2
– Community Banner	Community Event
– *Faces of Dignity* Banner	Community Event
– Flipchart for Family Forum 1 and Family Forum 2	FF1 | FF2
– Trachoma Transmission Routes – Magnetic board	FF1
– Trachoma Transmission Routes – Diagram	FF1 (follow-up visits)
– Face Wipe Emo-Demo – Washing protocol sheets	FF1
– “Dignified Day” Flashcards	FF2

List of all Giveaways for participants

NB. Households in the ‘yolk’ only receive the giveaways given Before and After the Community Event, i.e. an invitation, a branded wash station, a soap dish, 2 soaps, a wash station flyer.

Giveaway	Corresponding Event
– Invitation for Community Event	Before Community Event
– Branded wash station	After Community Event
– Soap dish	After Community Event
– Soaps (3 in total)	After Community Event |FF2
– Wash station flyer	After Community Event
– Line drawing of the puppet and wax crayons	FF1 | FF2
– Poster “Do you ensure your family’s Days are Dignified?”	FF1
– Soapy water bottle	FF2
– Dangler	HC1
– Certification sticker	HC2

COMMUNITY EVENT

NB. Refer to Community Event Manual for the full details of the below activities.

**Purpose**	This event is designed to raise awareness and credibility of the *Faces of Dignity* campaign among the community, to create buy in (especially from men) and to begin to build washing-related knowledge and motivation.
**Responsible parties**	3 trained Actors
**Supporting individuals**	2 trained Health Volunteers (HVs); community leaders; identified “Influential Role Models”; other volunteers if needed (to be adjusted during the pilot-testing of the intervention)
**Participants**	All household members living in the intervention cluster (‘white’ + ‘yolk’), community leaders
**Location**	Health post, garee office or kebele office compound (selected in advance)
**Duration**	1 hour

Preparation

- Community sensitisation
- Invitation distribution by Health Volunteers
- Set up on the day of the event

Activities

- Activity 1: Megaphone announcement & Music Playing
- Activity 2: Influential Role Model Introduction
- Activity 3: *Faces of Dignity* Drama
- Activity 4: *Faces of Dignity* Pledge
- Activity 5: *Faces of Dignity* Banner & Record Testimonials
- Following the Community Event: Distribution of Campaign materials

Collaterals used during the Community Event

- Puppet of the *Faces of Dignity* Drama
- Community Banner
- *Faces of Dignity* Banner

Giveaways received by participants

Before the Community Event

- 1 invitation for the Community Event per household

After the Community Event

- 1 branded wash station per household
- 1 soap dish per household
- 2 body soaps per household
- 1 wash station flyer per household

FAMILY FORUM 1

NB. Refer to Family Forum 1 Manual for the full details of the below activities.

**Purpose**	This event is designed to build washing related knowledge, skills, and motivation, and empower households to construct wash stations to aid habit formation.
**Responsible parties**	1 trained Activator + 1 trained Health Volunteer (2 HVs will assist the Activator in each cluster to spread the work load)
**Participants**	All household members living within the 'yolk' of an intervention cluster who attended the Community Event – Split into groups of 5 households.
**Location**	A HH compound (selected in advance when HHs are informed the time and date for the forum)
**Duration**	1h30

Preparation

- Recruitment of households by HVs
- Set up on the day of the event

Activities

- Activity 1: Introduction
- Activity 2: Trachoma Transmission Routes
- Activity 3: Face wipe Emo-Demo
- Activity 4: Wash stations – Constructing a stand and wash station use
- Activity 5: Testimonials
- Activity 6: Dignified Day Pledge
- Activity 7: Conclusion
- Following Family Forum 1: Individual follow-up visits with any non-attending household

Collaterals used during the Family Forum 1

- Puppet of the *Faces of Dignity* Drama
- Flipchart for Family Forum 1 and Family Forum 2
- Trachoma Transmission Routes – Magnetic board
- Trachoma Transmission Routes – Diagram (for follow-up visits only)
- Face Wipe Emo-Demo – Washing protocol sheets

Giveaways received by participants

- 1 line drawing of the puppet per child
- 1 poster "Do you ensure your family's Days are Dignified?" per household

FAMILY FORUM 2

NB. Refer to Family Forum 2 Manual for the full details of the below activities.

**Purpose**	Continue to build skills and motivation to practice face washing. Overcome early barriers related to wash station construction and use. Emphasize the need to wash faces with soap three times a day now that everyone has a functioning wash station.
**Responsible Parties**	1 trained Activator + 1 trained Health Volunteer (2 HVs will assist the Activator in each cluster to spread the work load)
**Participants**	All household members living within the 'yolk' of an intervention cluster who attended Family Forum 1 – Split into groups of 5 households.
**Location**	A HH compound (selected in advance when HHs are informed the time and date for the forum)
**Duration**	1h30

Preparation

- Recruitment of households by HVs
- Set up on the day of the event

Activities

- Activity 1: Introduction
- Activity 2: Live Testimonials
- Activity 3: Wash-Along
- Activity 4: "A Dignified Day"
- Activity 5: Barriers to Facewashing & Solutions
- Activity 6: Soap Giveaway
- Activity 7: Conclusion
- <u>Following Family Forum 2:</u> Individual follow-up visits with any non-attending household

Collaterals used during the Family Forum 2

- Puppet of the *Faces of Dignity* Drama
- Flipchart for Family Forum 1 and Family Forum 2
- "Dignified Day" Flashcards

Giveaways received by participants

- 1 soap per household
- 1 line drawing of the puppet and 1 wax crayon per child
- 1 soapy water bottle per household

HOUSE CALL 1

NB. Refer to House Call 1 Manual for the full details of the below activities and Checklist.

**Purpose**	To provide ongoing support to motivate families to wash faces thoroughly with soap x3 a day.
**Responsible parties**	1 trained Health Volunteer (HV)
**Participants**	All members of a household present at the time of this unannounced visit. Households living within the 'yolk' of an intervention cluster who attended Family Forum 2.
**Location**	Each participant's home
**Duration**	20 to 30 mins

Preparation

- Provide HV with materials required and refresher training for House Call 1

Activities

- Activity 1: Introduction
- Activity 2: Facial cleanliness assessment and Face Washing
- Activity 3: Wash station review
- Activity 4: "A Dignified Day" poster review
- Activity 5: Dangler giveaway
- Activity 6: Conclusion

Collaterals used during the House Call 1

No collateral used.

Giveaways received by participants

- 1 dangler per household

House Call 2

NB. Refer to House Call 2 Manual and House Call 2 Checklist for the full details of the below activities.

**Purpose**	To provide ongoing support to motivate families to wash faces with soap 3x a day throughout the year. Provide support to help families maintain their wash stations.
**Responsible parties**	1 trained Activator
**Participants**	All members of a household present at the time of this unannounced visit. Households living within the 'yolk' of an intervention cluster who received House Call 1.
**Location**	Each participant's home
**Duration**	30 to 45 mins

Preparation

No specific preparation required except preparation of materials

Activities

- Activity 1: Introduction
- Activity 2: Video Demo
- Activity 3: Wash station review
- Activity 4: Wash Station Maintenance
- Activity 5: Poster and Dangler Review
- Activity 6: Wash Station Certification
- Activity 7: Conclusion
- Following House Call 2: Community leaders will be gathered at the location where the Dignity Banner is up to publicly declare the community a Dignified Community. The Dignity Banner will be amended at the occasion. Consecutively, community leaders will be rewarded for their contribution to the intervention. *This section will be amended after discussing options with the field team and Berhan.*

Collaterals used during the House Call 2

- Puppet of the *Faces of Dignity* Drama

Giveaways received by participants

- 1 certification sticker per household
