# Supplementary material for: Cluster randomised controlled trial of double-dose azithromycin mass drug administration, facial cleanliness and fly control measures for trachoma control in Oromia, Ethiopia: the stronger SAFE trial protocol
Source: BMJ Open. 2024 Dec 23;14(12):e084478. doi: 10.1136/bmjopen-2024-084478 (PMC11751794; doi:10.1136/bmjopen-2024-084478)
Supplement: online supplemental file 2 [file bmjopen-14-12-s002.pdf]

EVENT 1 – COMMUNITY EVENT
ACTIVATOR MANUAL

**Purpose**	This event is designed to raise awareness and credibility of the *Faces of Dignity* campaign among the community, to create buy in (especially from men) and to begin to build washing-related knowledge and motivation.
**Responsible parties**	3 trained Actors
**Supporting individuals**	2 trained Health Volunteers (HVs); community leaders; identified "Influential Role Models"; other volunteers if needed (to be adjusted during the pilot-testing of the intervention)
**Participants**	All household members living in the intervention cluster ('white' + 'yolk'), community leaders
**Location**	Health post, garee office or kebele office compound (selected in advance)
**Duration**	1 hour
**Timing**	Mondays, Wednesdays, Sundays (avoiding local market days) – Morning from 9am to 1pm

Preparation

During the week before the event

Meet community leaders at cluster level (zone / garee leaders, clan / religious leaders, HVs / HEWs):

- If possible, hold the meeting in an outdoor location. Ensure you are wearing a facemask during the entire meeting. Wash your hands with soap or alcohol-based sanitizer before and after the meeting. Ensure you are safely disposing your facemasks in a sealable plastic bag.
- Remind them about the Stronger-SAFE trial and their community's allocation to the intervention arm (or communicate this if it has not already been discussed). Remind them which interventions they will receive. **Refer to Appendix A** for guidance on Community Sensitisation.
- Introduce the *Faces of Dignity* campaign and encourage them to support it.
- Brief them on the content of the Community Event.
- Identify a suitable and accessible location, date and time for the Community Event.
- Discuss who should assist with the set up and running of the Community Event.
- Identify prominent location to display the community's *Faces of Dignity* banner.
- Request the presence of all community leaders at the Community Event.
- Finalise selection of leaders to speak at the Community Event in the role of "Influential Role Models" – ensure Influential Role Models can read if anything written has to be read at loud. **Refer to Appendix B.**
- **Give wash stations, soaps, soap dishes and Campaign hi-vis jacket to Health Volunteers. Give wash stations, soaps, and soap dishes to Influential Role Models***. Explain why it is important that they model and start practicing the desired behaviour.
- Assess willingness / availability of Influential Role Models to intervene at the Community Event and voice-record Testimonials at the end of the Community Event. Purpose of Testimonials can be found in Appendix C.
- Arrange for storage of Wash Stations (soap dishes, soaps and wash station flyer) for the cluster (~90) in a secure location until they are distributed after the Community Event.

Refer to **Appendix D** for guidance on how to organise the wash station and giveaways distribution.

**NB: This may be amended. Likely to give wash stations, soaps, and soap dishes to the Kebele leader, Zone Leader and HEW as well. "Influential Role Models" might also receive campaign caps.*

Day before the event*

- Provide two HVs with the census list and invitations for the Community Event.
- Instruct HVs to go house-to-house to distribute 1 invitation to each family. If the primary caregiver or the household head are not at home, HVs can distribute the invitation to any child over the age of 14 who is a household member. HVs should ask this child to inform his/her parents about the event.
- Brief HVs on the invitation pitch and what to do if they identify anyone who needs support getting to the Community Event. As the Community Event might be the only event where household heads come, HVs should emphasize that both female primary caregiver and male household head are invited to come to the event with their children.

> **"We will be holding a drama tomorrow at [chosen place] at [chosen time] where you will learn how to make sure you and your family have *Faces of Dignity*. You will be informed where and when to get a gift at the end of the drama. Make sure you come on time so you do not miss the music and introduction by a surprise and special guest.**
>
> **Your whole family is invited. We can arrange for assistance if any members of your family will find it difficult to attend the event, for example because they are unable to walk to [chosen place] or sit comfortably."**

**NB: If it is logistically difficult to visit the cluster again before the event, instruction for recruitment should be given during the Sensitisation meeting outlined above. HVs should be requested to deliver invitations the day before the Community Event whenever possible to increase attendance. HVs will be called the day before the event to remind them to distribute invitations if they are given the invitations early.*

Setting

The sketch below illustrates the ideal setting for the community event. Activators are free to amend the set-up according to each health post/garee office/kebele office. Any setting should respect the following criteria as much as possible:

- Participants should be able to SEE and HEAR the full event easily no matter where they are sitting (test the microphones).
- Elderly or disabled participants should be directed to sit on chairs or stools at the back or sides of the stage (wherever they will not obstruct the view of anyone else).
- Participants should be comfortable i.e. they should be sat in the shade / should be able to view the stage without being blinded by the sun.
- Materials for the drama should be organised and located out of sight of the audience as much as possible.
- Participants should sit with their household members and observe a physical distance of at least 2m with other households, where possible.

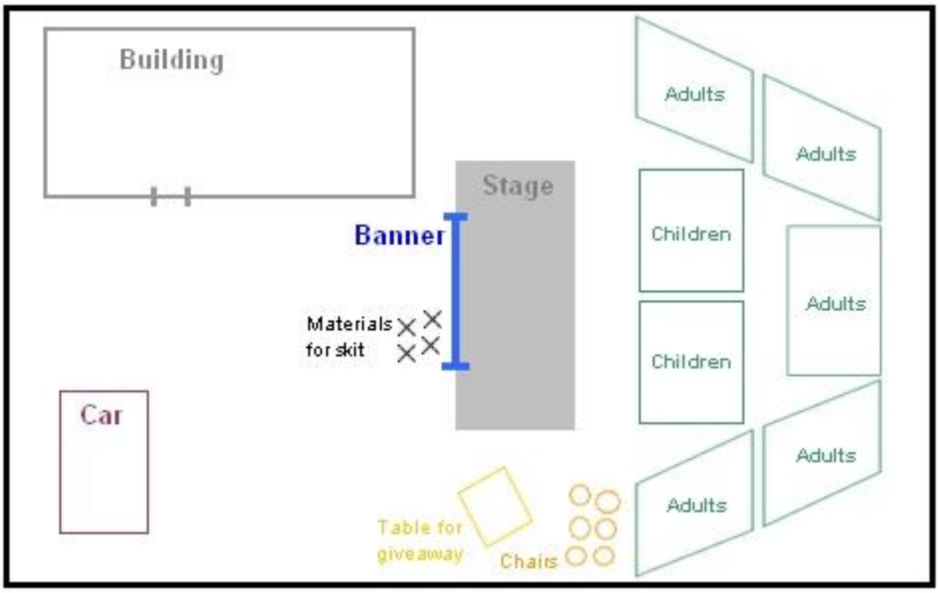

Materials

General

- COVID-19 preventive materials: cleaning solution in sprays (x4), facemasks (all support responsible parties, supporting individuals, and people needed transportation arrangements) sealable plastic bag to dispose masks
- Megaphone
- Logo banner
- Microphones + Speakers + Power bank
- Plastic stools for HEW/HV/Fieldworker/Influential speakers/any participants who cannot sit on the ground (borrowed from Shashamane office or in the cluster)
- A bell (used in the drama)

Drama

- Clothing for actors
  Actress: dress, scarf, jewels, shoes
  Actor: scarf, shoes
  Puppet actress or actor: neutral / black clothing)
- Caltu's puppet
- Plastic stools (x2)
- Coffee tray + jebena + 6 coffee cups
- Jug + collector + soap dish + soap
- Jerry can of 20L filled with water
- Wash station

***Faces of Dignity* Pledge**

- Wash station + soap dish + soap (reuse from the drama)
- Palm cards for actors
- *Faces of Dignity* banner
- Permanent markers for outlining hands on the banner
- Rope/Nail to put up the banner at the end of the event

Testimonials recording

- A phone or device with a microphone

Set up

Day of the community event

- Do you have all materials required for all activities?

- Is equipment working?
  - Megaphone, microphones and speakers are charged/have full batteries.
  - Quality of sound has been checked from all angles.
- Have chairs / stools been set out?
- Are the 'Influential Role Models' present? Have they been briefed?
- Have the Health Volunteers been briefed on their role?
- Have additional volunteers been identified to help with the set up and running of the event if needed? *NB: the need for this will be assessed during piloting.*

Activities

Activity 1: Megaphone announcement & Music Playing

Purpose – To remind the population that the community event is about to commence (i.e. to increase attendance).

What to do

In the community

1. The HVs should announce the event by megaphone on foot or using a motorcycle/car, depending on the terrain. The announcement should tell everyone to make their way to the chosen location and explain that the whole family should attend.
2. Upon arrival at the event location, supporting individuals (HVs, activators, etc.) should advice community members to sit with their household members and respect a physical distance of at least 2m with other households.
3. HVs should liaise with community leaders to ensure any community members needing assistance (e.g. people with disability) are supported to get to the event e.g. transportation is provided to and from the event and they are guided to a comfortable seat. Community members who are transported to the event location should be given facemasks before entering the vehicle.

At the event

4. Actors play the Dignity Song on the event speakers as people gather.
5. Actors ensure Community Leaders / Influential Role Models are present and briefed on what to say during the introduction and after the drama.

Activity 2: Influential Role Model Introduction

Purpose
- To increase the credibility of the intervention and increase willingness of participants to be part of the programme.
- To provide role modelling and create the impression that important others in the community are already washing faces with soap 3x a day.
- To trigger the feeling of ownership (of the programme) among the community leaders.

What to do

1. Stop the music and ring a bell to get the crowd's attention.
2. Invite the three chosen Influential Role Models to take the stage with you (the Actors) and the Campaign Health Volunteers. Get everyone's attention. Do not begin until the crowd is quiet.
3. Ask the leader of the chosen Influential Role Models to welcome the community to the event and to introduce you and the HVs dedicated to working on this Campaign.
4. Ask the following question to the three Influential Role Models:

> Dignity: **"Do you value your dignity and why?"** Let the Influential Role Models answer.
> Children's dignity: **"Do you value your children's dignity and why?"** Let the Influential Role Models answer.
> Community's dignity: **"Do you believe that the dignity of everyone in the community, i.e. the community's dignity is important too? Why?"** Let the Influential Role Models answer.

5. Thank them for caring so much about their community and tell them that you are here today to tell them how they can ensure they all have *Faces of Dignity*. Invite the Leaders to leave the stage so you can begin.

Activity 3: *Faces of Dignity* Drama

Purpose

- Improve understanding that trachoma is spread by flies and on hands and that washing faces with soap can remove discharge and prevent trachoma transmission
- Give an overview of the key face washing messages of the campaign in a humorous and memorable way, including the importance of soap for face washing
- Cause people to revalue face washing and link the behaviour to the *Attract*, *Affiliation* and *Status* motives (the latter two via dignity)
- Create the impression that family members and neighbours expect you to wash your face and the faces (and hands) of your children 3x a day with soap

NB. **Refer to Appendix E** – *Script for the Faces of Dignity Drama.*

Activity 4: *Faces of Dignity* Pledge

Purpose

- Perceive soap as important to use each time faces are washed
- Perceive effectively washing face at least 3x a day as important, all year around
- Accept responsibility for hygiene of young children
- Perceive an expectation from neighbours & husbands to maintain clean faces of self & family
- Perceive an expectation from parents to maintain clean faces

What to do

Influential Role Models' testimonials and face washing demonstration

1. Invite the Influential Role Models to come back on stage and bring forward the wash station, soap dish and soap used during the drama.
2. Ask the Influential Role Models if they would like their family to have *Faces of Dignity* and why.
    a. Each Influential Role Model should quickly tell one's own story having and using a wash station and making sure soap is always available for body washing. **Refer to Appendix C** for guidance on topics which can be covered by the Influential Role Models.
    b. Ask each Influential Role Model to come forward and demonstrate how to wash their hands and faces with soap using the station. They should be mindful of closing the tap while rubbing their faces and hands.
    c. Thanks the Influential Role Models for being *Faces of Dignity* in their community.
3. Ask participants to raise their hands if they too, like Caltu's family and the Influential Role Models, would like to have *Faces of Dignity*.

Faces of Dignity banner

1. Uncurl the *Faces of Dignity* banner and hold it at each end.
Say that we can do some activities to help us all ensure our families have *Faces of Dignity*. Explain that each family committing to have *Faces of Dignity* will also contribute to enhancing their community's dignity. All faces should be dignified for the community to be dignified.
Invite the leader of the Influential Role Models to read out the activities on the banner.

> **I wash my face and hands with soap when I wake up, before lunch and before my evening meal.**
>
> **I help my pre-school children wash their faces and hands with soap when they wake up, before lunch and before their evening meal.**
>
> **I help my family maintain their *Faces of Dignity* and contribute to enhancing my community's dignity.**

2. Ask participants to raise their hands if they agree that someone doing these activities would have a *Face of Dignity* and contribute to enhancing the dignity of their community
3. Ask the Influential Role Models if they intend to ensure their family does these activities to have *Faces of Dignity* and contribute to enhancing their community's dignity
4. Ask the crowd if they want to do these activities to have *Faces of Dignity*.
5. Ask the crowd if they want to become *Faces of Dignity* to contribute to enhancing their community's dignity.

Collective Pledge

1. Tell the crowd that we are going to pledge to do these activities together as a community.
2. Ask the leader of the Influential Role Models to read out the pledge slowly and ask the audience to stand up with family members and repeat after him/her:

> **I pledge to maintain my *Face of Dignity*, ensure my Family all have *Faces of Dignity*, and contribute to enhancing my community's dignity.**

3. Congratulate all participants on their pledge. Inform them that they will be supported and monitored on their progress to become *Faces of Dignity* in the coming weeks. In a few weeks' time, if all members of the community have *Faces of Dignity*, the Influential Role Models and community leaders will be able to declare their community to be a Dignified Community and amend the banner.
4. Conclude the event by informing the community that:
    a. In each household, **the household head or the female primary caregiver can go to [the chosen place] on the [chosen days]** to pick up their wash station, soap dish and soaps. **Refer to Appendix D** for guidance on how to organise the wash station distribution.
    NB. Exact logistics on how and when the wash stations will be given to the community will be decided on a cluster-to-cluster basis and might be amended during pilot-testing of the intervention.
    b. Say that some ongoing activities and small group meetings will continue in the community, but it will not be possible to visit all households.

Signing the *Faces of Dignity* banner

1. Stretch the banner out on the ground and put out the permanent markers.
2. Invite the Influential Role Models to come forward and "sign" the banner by outlining their hand with a marker.
3. Invite each household to send one child (accompanied by the caregiver if possible) to "sign" the banner. This activity is supervised by one Actor, one Health Volunteer and one Influential Leader.
    a. Actor and HV should wear a facemask during this activity as they will be in close contact with children and caregivers.

b. Between each child, the Actor or the HV should disinfect the marker using a cleaning spray.
c. After the child has signed the banner, the accompanying adult should be directed to Giveaway 'stand' to receive their 2 soaps.

4. Play the Dignity Song on the loud speakers (if it does not interfere with the activities).

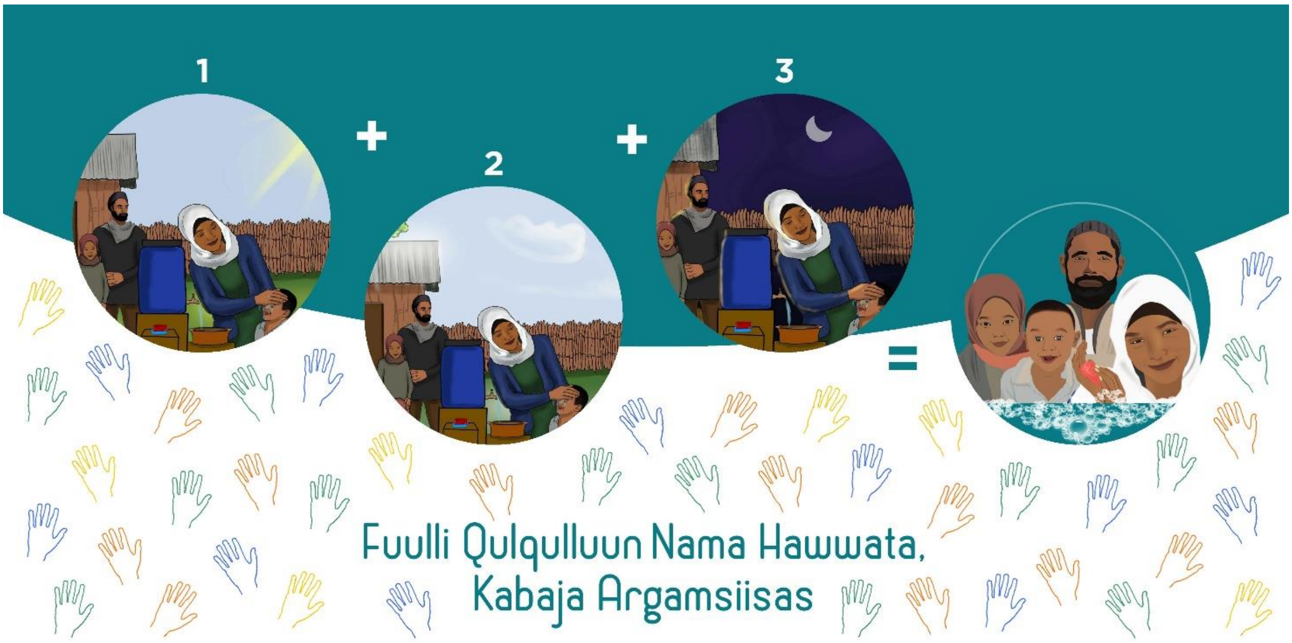

ACTIVITY 5: *FACES OF DIGNITY* BANNER & RECORD TESTIMONIALS

Purpose

- Give the community a permanent reminder of the key messages of the campaign and their pledge
- Create the impression that everyone in the community washes faces with soap x3 a day

What to do

1. Put the *Faces of Dignity* banner up at the designated location chosen by the Community Leaders during sensitisation, with the help of the HVs and any volunteers.
2. Before they leave, voice-record testimonials with Influential Role Models using a smartphone or a voice-recorded. They should basically repeat and elaborate on what they said after the drama. **Refer to Appendix C** for guidance on topics which can be covered by the Influential Role Models. *NB. To be adapted during pilot-testing to see whether it is feasible. Might be done with the Lead Influential Role Model only.*

End of Community Event.

Appendices

Appendix A. “Guidance on Community Sensitisation for the *Faces of Dignity* Campaign”

Purpose

- Remind or inform community leaders about their allocation to an intervention arm.
- Remind or inform community leaders about the interventions their community will receive.
- Answer any question related to the trial or intervention design.

Responsible parties

- Berhan Supervisors are responsible for ensuring the Community Sensitisation is appropriately done in each cluster prior to the delivery of the Campaign. In case Berhan Supervisors cannot attend the meeting, Berhan Actors or Activators should cover the Community Sensitisation content.

Procedures

NB. If possible, hold the meeting in an outdoor location. Ensure you are wearing a facemask during the entire meeting. Wash your hands with soap or alcohol-based sanitizer before and after the meeting. Ensure you are safely disposing your facemasks in a sealable plastic bag.

1. Ask the community leaders if they have already been informed about their allocation to an intervention arm.
    a. If they have already been informed (e.g. at start of MDA), ask them to summarize what interventions they are expecting to receive and correct any misunderstandings. Describe intervention packages the community will receive according to their allocation arm (details provided in the boxes below).
2. Ask the community leaders if they have any questions on their allocation to the intervention arm or on the interventions their community will receive.
3. Introduce the *Faces of Dignity* Campaign:

- Intensive and inter-personal intervention targeting around 90 (to be adapted to each cluster size) households in some garees of the zone.
- Communication is at 3 levels: community (1 meeting with a community drama), small group (2 forums with groups of 5HHs) and household (2 household visits). Only 50 households will receive the small groups and household visits.
- Campaign (i.e. 5 visits) will take place over 1 month and be followed by further “reinforcement” group meetings will take place periodically throughout the year, timed to coincide with key seasonal barriers to face washing.
- The intervention involves provision of the following hardware and props: branded water containers with faucets, branded soap dishes, bars of body soap.

4. Introduce the Community Event:

- Large scale event delivered by 3 trained actors and involving Influential Role Models from the community as well as Health Volunteers.
- **Purpose:** raise awareness and credibility of the Campaign among the community and begin to build wash-related knowledge and motivation.
- About 90 households (to be adapted to each cluster size) will be invited as well as all community leaders (including those present at this meeting)

5. Ask the community leaders if they have any questions about the Campaign.

Allocation arm: "**Standard** A intervention and **enhanced** F&E interventions"

1. Remind community leaders about trachoma, i.e. an eye disease caused by a germ called *Chlamydia* that many people catch in Ethiopia. It can cause people to go blind.
2. Remind them about the Stronger-SAFE trial, i.e. aiming to study the best ways to remove trachoma from your community.
3. Explicitly announce to the community leaders which intervention arm their community has been allocated to, i.e. your community will receive the current antibiotic treatment (single-dose treatment) and some households will receive hygiene messages or new fly control interventions (which households receive these tools will be decided randomly (like tossing a coin)).
4. Quickly remind them about the trial's demands on the participants:
    a. Test for trachoma (eye and face examinations), up to eight times over the next three years
    b. Fly control measures, including fly trap and anti-fly headwear for children
    c. Some households will receive hardware to aid hygiene behaviour (not everyone in the zone)
    d. A WASH intervention, covered in more details after, including a community event, two small-group visits, two household visits and additional reinforcement visits periodically
    e. Voluntary participation to extra studies on fly control or hygiene interventions

Allocation arm: "**Enhanced** A intervention and **enhanced** F&E interventions"

1. Remind community leaders about trachoma, i.e. an eye disease caused by a germ called *Chlamydia* that many people catch in Ethiopia. It can cause people to go blind.
2. Remind community leaders about the Stronger-SAFE trial, i.e. aiming to study the best ways to remove trachoma from your community.
3. Explicitly announce to the community leaders which intervention arm their community has been allocated to, i.e. your community will be offered two doses of antibiotic (given 2 weeks apart) and some households will receive hygiene messages or new fly control interventions (which households receive these tools will be decided randomly (like tossing a coin)).
4. Quickly remind about the trial's demands on the participants:
    a. Test for trachoma (eye and face examinations), up to eight times over the next three years
    b. Fly control measures, including fly trap and anti-fly headwear for children
    c. Some households will receive hardware to aid hygiene behaviour (not everyone in the zone)
    d. A WASH intervention, covered in more details after, including a community event, two small-group visits, two household visits and additional reinforcement visits periodically
    e. Voluntary participation to extra studies on fly control or hygiene interventions

Appendix B. "Identifying Influential Role Models"

Purpose of the Influential Role Models

- Provide role modelling of the target behaviour within their local area.
- Introduce the *Faces of Dignity* Campaign during the Community Event.
- Record testimonials which will form part of the intervention content in each local area.

Specific roles

- The involvement of Influential Role Models in the *Faces of Dignity* Campaign will be introduced to the community formally during the Community Event. The lead Influential Role Model will introduce the programme and Actors and explain why it is important.
- They will be asked to spend few hours providing a voice-recorded testimonial about using the wash station and practicing face washing with soap 3x a day, including how they have overcome any obstacles associated with these behaviours. The Testimonials will be used during Family Forum 1, House Call 2 and the Reinforcement events (if possible).
  NB. This might be revised after pilot-testing in the field according to feasibility.

Selection

- Three Influential Role Models will be selected to voice-record a testimonial for Family Forum 1 (3 testimonials). The "Lead" Influential Role Model will be asked to provide an additional testimonial for House Call 2 to congratulate participants on their efforts. Voice-recording will take place at the end of the Community Event.
- Influential Role Models will be selected based on the following:
  1. Influential and respected community member suggested by HEWs, e.g. clan or religious leader, or by community members themselves in baseline surveys.
  2. Self-reported capacity and interest to undertake the required work (participation in the community event and recording of testimonials).
  3. Positive attitude to face washing and hand hygiene.
  4. Confirmation that they are influential and viewed positively in the community by key community members e.g. teachers and HEWs/HVs.
  5. Phone ownership.

Timing of selection

- Influential Role Models will be identified during early contact with the cluster (ideally during baseline data collection) by the Stronger-SAFE field team. If that is not possible, they will be identified around sensitization for the *Faces of Dignity* Campaign by the Stronger-SAFE team in conjunction with Berhan.

Training

- No specific training is foreseen to engage the Influential Role Models. They will be briefed on their roles and what is expected from them for the Community Event and testimonials at the sensitisation meeting, the day before the Community Event and on the day of the event/testimonials recording.

Motivation

Influential Role Models will receive:

- All intervention materials (wash station container, soap dish, soap, etc.)

- A cap with the campaign logo they should wear during the community event and testimonials recording. *NB. Incentives might be revised according to budget and feasibility.*

Appendix C. "Recording Influential Role Model Testimonials"

Purpose of the Testimonials

- To role model use of a wash station to practice face washing with soap 3x a day and suggestions of how to overcome barriers associated with this practice.
- To increase the legitimacy of the *Faces of Dignity* Campaign.

Testimonials to record

- Four testimonials per intervention cluster should be voice-recorded at the end of the Community Event. Each of the Influential Role Models will record one testimonial for the Family Forum 1. The lead Influential Role Model (designed among the three Role Models) will also record the *"Congratulating participants"* for House Call 2.
    a. Family Forum 1: (1) *"Building a wash station"*, (2) *"Maintaining a wash station"*, (3) *"Routine use of a wash station"*.
    b. House Call 2: (4) *"Congratulating participants".*

  NB. Full content of each testimonial can be found at the end of this guide as well as in the relevant Family Forum and House Call guides.

  Process for recording testimonials (voice recording or video recording) will be finalised during the pilot-testing of the intervention.
- In the event that it is not possible to record all four testimonials in a cluster, testimonials should be recorded in the following order of priority:
    a. *"Congratulating participants"* for House Call 2.
    b. *"Maintaining a wash station"* for Family Forum 1.
    c. *"Routine use of a wash station"* for Family Forum 1.
    d. *"Building a wash station"* for Family Forum 1.

  NB. If the lead Influential Role Model is not available to record any testimonial, the "Congratulating participants" should be recorded with either of the other two Influential Role Models.

Personnel

- Testimonial recording will be supervised by the Stronger-SAFE field team in conjunction with Berhan's trained Actors/Activators.

Timing

- Recording of the Testimonials will take place at the end of the Community Event.

Materials

- A smartphone or a voice-recorder.

Procedures

1. Remind Influential Role Models about the purpose of the Testimonials and what they will be used for in the intervention (videos shown during Family Forum 1 and/or House Call 2).
2. Confirm their willingness to record the testimonials, based on what they said earlier during the Community Event vent.
3. Brief the Influential Role Model about the content of the testimonial you want to record with her/him (e.g. *"Building a washing station"*). Explain that you will ask them questions related to their washing station to which she/he should answer based on their own experience. Use the "model" testimonials below to probe Influential Role Models.

4. Inform the Influential Role Model that before recording the testimonial, you would like to do a practice round.
5. Do a practice round with the Influential Role Model and provide feedback about the content if needed. Repeat Step 5 if necessary.
   NB. The Influential Role Model does not have to cover the exact content provided in the "model" testimonials but should cover relevant points and should share their own experience in line with the topic as naturally as possible. Guide them using the "model" testimonial if they have difficulties deciding what to say.
6. Test the sound by recording a brief conversation and playing it back. Delete the recording once you are satisfied with the quality.
7. Tell the Influential Role Model that you will now record the testimonial which will be played to their community.
8. Before starting the recording, check that: the environment is quiet.
9. Voice-record the testimonial using the smartphone / voice-recorder.
10. Playback the testimonial and check the quality. Check the content of the testimonial is aligned with expectations and that the Influential Role Model is happy with the content.
11. Repeat the recording of the testimonial if the quality and content are not in line with expectations.
12. When you are satisfied with the recorded testimonial, thank the Influential Role Model and end the visit.

Content of the Testimonials – "Model" testimonials

For Family Forum 1

- Testimonial: ***"Building a washing station"*** | Interviewee: Male Influential Role Model
  Question: Why did you decide to build a wash station stand?
  Answer: I built a wash station stand for my wash station so my family and I could wash our faces with soap easily so we have *Faces of Dignity* in the community and.
  Question: How did you decide on the location and the height of your station?
  Answer: I built my wash station stand near to the entrance of our house so the wash station is protected from the sun and easily moveable at night, when I bring the wash station container, the soap dish and the soap inside. I built the stand like this so my older children can use it easily, but my younger children, who are supported by my wife or myself to wash, cannot play with the soap and make it dirty, or with the tap and waste the water.
  Question: Was it hard to build a wash station stand?
  Answer: Not at all! *They should explain what they did. E.g.* It was easy and quick to build the stand with some wood I collected. I made a strong structure and then put the container on top of it. I dedicated a baldy to collect the wastewater, and added a dedicated body soap which I put in the soap dish near the container. It was done in less than 2 hours.

- Testimonial: ***"Maintaining a washing station"*** | Interviewees: Influential Role Model with her husband/his wife
  Question: Have you set some roles for taking care of the washing station in your house?
  Answer (male): Yes, we have. *They should explain what they have done. E.g.* At first it was not easy to get used to the station, moving the wash station container, filling up the water, always having a dedicated soap. That's why my wife and I decided to set some roles so we don't forget anything. I take care of the materials, bring the station in and out in the morning and at night. My wife is responsible for water and soap and making sure the station is always secured during the day. For instance, that the children do not play with the tap.
  Question: Is your wash station always staying outside?

Answer (male): *They should explain what they have done. E.g.* At first, we thought about leaving the washing station outside at all time, but we had nothing good enough to secure it from wild animals or to make sure we do not lose everything overnight. So, our wash station is brought inside our home at night. Because I am often the one waking up and going outside first in the morning, I am the one responsible for putting the wash station container and the soap and soap dish on the washing station stand every morning. At night, after the evening wash, I am also the one responsible for bringing the wash station container and the soap dish inside the house when I close the door. I never forget to put the station outside in the morning or inside at night because the wash station stand is visible from the door of our house.
Question: How do you make sure water is always available at the station?
Answer (female): *They should explain what they have done. E.g.* I am usually the one responsible to fetch water every morning. Since we have the station, I have dedicated the water of one of our jerry cans to fill the washing station. Like this, that is really easy. I don't have to worry about having enough water for washing 3x a day. Often, there is even some water left inside the washing station container at the end of the day for the day after.
Question: How do you make sure a dedicated body soap is always available at the station?
Answer (female): *They should explain what they have done. E.g.* Each time I notice that the soap is going to run out soon, I ask my husband for money to buy a dedicated soap for face and body washing. He always gives me money or buys the soap himself. When possible, I buy two soaps at the same time to make sure we always have a spare soap. We thought it would be difficult to keep soap outside because we are not used to do this, but it works and helps us remember to use soap when we wash. That is how we are maintaining our washing station to make sure our family has *Faces of Dignity*.

- Testimonial: ***"Routine use of a washing station"*** | Interviewee: Influential Role Model
  Question: How do you remember to wash your family's faces three times a day?
  Answer: In the morning, we used to wash our faces before having the station, so that is easy. But now we have the station, I never forget about using the soap and supporting my younger children so I can wash their eyes and noses thoroughly. I supervise the older ones too. Before lunch, no one has ever forgotten about face washing with soap, as we are also washing our hands before eating at the wash station. We have associated washing hands and washing faces. That is a good reminder. In the evening, we all wash our faces and hands before dinner, our children even remind us about it. In our family, everyone knows about face washing 3x a day with soap, morning, before lunch and before dinner.
  Question: What do you like about using the washing station?
  Answer: The washing station has made our lives so much easier and we have saved a lot of water. Now we have it, it is so much simpler to maintain our family's *Face of Dignity* by washing our children's faces three times a day with soap. With our wash station and soap always nearby, it is so simple. In my family, we have *Faces of Dignity*, do you?"

For House Call 2

- Testimonial: ***"Congratulating participants"*** | Interviewee: Lead Influential Role Model
- "Congratulations for working so hard to become a dignified family and all having *Faces of Dignity*. I personally want to thank you all for being dignified community members. Your community is proud of you as everyone needs to work together to ensure our community all have *Faces of Dignity*. Thanks to all of you, our community is a Dignified Community! The certification sticker which is given to you now acknowledges your efforts over the last month to wash your faces x3 with soap a day to become *Faces of*

Dignity. Like me, stick it on your wash station container to always remember your progress. Congratulations again and keep up the good work!"

Appendix D. "Wash station distribution for all households following the Community Event"

Purpose To distribute the wash station container, soap dish, soaps and wash station flyer to all households ('yolk' + 'white').

Setting Central location in the cluster (e.g. health post, garee office, etc.) – According to the outcome of the discussion with community leaders during the Campaign sensitisation. This should be the place where all the wash stations are stored. Distribution can take over 2 to 3 days according to HVs' and community leaders' availability.

Personnel At least 2 people. One or 2 intervention Health Volunteers (HVs) and/or community leaders supervised by a trained Activator from Berhan.

Timing Just after the Community Event for 2 to 3 days according to personnel's availability.

Materials

- Wash station and giveaways distribution form (based on census)
- 2x pens
- 1 table + 2x plastic stools
- Wash station containers (1 per HH), soap dishes (1 per HH), soaps (2 per HH), wash station flyers (1 per HH) for all households ('yolk' + 'white')
- Facemasks for HVs and community leaders, alcohol-based sanitizers, cleaning solution in a spray (x1), sealable plastic bag for mask disposal

Preparation

Day before the wash station distribution or Morning before the distribution

- All information about time and venue for the wash station distribution should be given at the Community Event.

Day of the distribution

- HVs put their facemask on and wash their hands with water and soap or alcohol-based sanitizer before and after the distribution.
- HVs set up a table and two plastic stools in the shade at the chosen place.
- HVs check they have enough wash stations, soap dishes, soaps and wash station flyers for all households ('yolk' + 'white')
- HVs check they have the "wash station and giveaways distribution form" to be completed.

Distribution

1. HVs sit at the distribution point and wait for households to come and pick up their wash stations and giveaways.
2. Each time a household arrives at the collection point, the HVs find the name of the HH on the "Wash stations distribution form" and give: **1 wash station container, 1 soap dish, 2 soaps and 1 Wash station flyer**.
3. The HVs also inform the household they would like to quickly discuss how to construct a stand for their wash station and how the station should be used.
   NB. If several households arrive at the same time, HVs can provide information regarding the construction of the wash station stand at the same time to a maximum of 3 households.
4. The HVs use the Wash station flyer to describe how to build and use the wash station. The following aspects should be covered:

a. **Location and Structure:** the station should be outside, close to the home and in the shade. The wash station and soap dish are on a built, sturdy wooden structure that is secured and cannot be knocked over by children or animals.
b. **Height:** if the household has children, very young children cannot reach the tap and soap without help, but the station should be accessible to all other family members.
c. **Drainage:** a bowl or stones/sand are placed under the tap to prevent the ground from becoming muddy.
d. **Water:** someone is responsible for filling the wash station with water so there is always water available for use. The wash station container should not be taken to the water, the tap is fragile. The tap should be closed whilst lathering the hands or rubbing soap on faces to avoid wastage.
e. **Soap:** a dedicated soap is kept in a soap dish at the wash station. The soap is always clean and available for body washing when it is needed.
f. **Night:** at night, if there is no fence around the station, it might be preferable to take the wash station container and the soap and the soap dish inside the house.

5. HV asks whether they have any question or concerns about how to set up their wash station.
6. HV encourages them to use the wash station to wash their family's faces with soap 3x a day (morning after waking, before lunch and before the evening meal) to maintain their *Faces of Dignity*.
7. HVs should **complete the "wash station distribution form"** for each HH who comes and receives the intervention materials.
8. At the end of the distribution day, households who did not come to receive their wash stations or did not attend the Community Event are visited by the HVs and inform that they can come to pick their wash station at [the chosen location] the day after. HVs should specify whether that this will be the last opportunity for them to receive their materials.
9. Similar procedures are followed on the second day of distribution.
10. Wash station distribution forms should be returned to the Berhan representative along with any remaining wash stations, so Berhan can complete the distribution of all remaining wash stations.
11. HVs should safely dispose each used facemask in a sealable plastic bag.

Appendix E. "Script for the *Faces of Dignity* Drama"

Update and Changes to the current Drama

It is expected that the Berhan Actors, in conjunction with the supervising team, will create the sections currently highlighted in 'yellow' in the drama, i.e. the **Dignity Stories**, during the intervention training (in a collaborative and iterative process). Specific instructions for these sections will be given separately ahead of the training.
Final version of these sections will need to be agreed upon by all supervision parties before rollout in the communities.

Other changes and tweaking of the current version of the drama during training are expected to happen. Yet, ALL changes made should be run through the supervision team for approval before final agreement.

Synopsis

The drama focuses on a young girl called Caltu (played by an Actor with a puppet) and the interactions between her parents (Hadha Caltu & Abba Caltu) and their neighbour (Hadha Marga) as the parents learn that to be truly attractive and dignified their family needs to wash their faces (and hands) with soap 3x a day and prioritise the cleanliness of Caltu. Hadha Caltu first tells the story of her encounter with Hadha Marga who told her that unclean faces are not attractive or dignified, and that faces and hands need to be washed with soap to maintain their dignity. In the next scene, Hadha Caltu discusses her conversation with Hadha Marga with her husband and they decide to wash Caltu's face with soap and water. They later realise that her face is dirty again and, following Hadha Marga's advice once more, they come to the conclusion that they should wash Caltu's face and hands 3x a day. At the end of the drama, Hadha Caltu and Abba Caltu are recognised as doing their bit to represent their community now they are washing their own and Caltu's face and hands three times a day with soap. Helping ensure their community is dignified makes them feel good!

Key messages

The *Faces of Dignity* campaign is about face (and hand) washing with soap. People are encouraged to <u>wash faces and hands with soap x3 a day</u> (after waking in the morning, before lunch and before the evening meal). Faces should be washed <u>thoroughly, removing all discharge.</u> This means that caregivers should support pre-school children during face washing. If a face is <u>wiped</u> to remove discharge, hands should be used instead of clothing or rags, and hands should be immediately washed with soap after the face is wiped. We are promoting washing faces together as a family to try to encourage habit formation and model the ideal behaviour, but pre-school children are the priority.

The drama aims to motivate people to adopt these behaviours by attaching new motives (dignity i.e. the root motives of *affiliation / status*, and to a lesser extent beauty) to face washing as opposed to promoting face washing with soap as a health behaviour. Face washing, and the use of soap, should come across to the audience as a really positive behaviour that makes them feel "good" inside and out.

NB. Hand washing is promoted alongside face washing, but of course there are many other times when hands should also be washed. We are not focussed on all the other handwashing messages, and just encourage hands to be washed whenever faces are (and after wiping a child's face to remove discharge). This is why the main messaging is about faces, but hands should not be forgotten.

Characters

Hadha Caltu (Caltu's mother): Female caregiver, Abba Caltu's wife – Played by the female activator

Outfit: a long dress, woman scarf, few cultural jewelleries (including bracelet, ring, necklace?), local plastic ballerina shoes). Outfit could be made from fabric with the logo of the campaign.

Abba Caltu (Caltu's father): Household head, Hadha Caltu's husband – Played by the male activator

Outfit: jeans, a long sleeve shirt (sleeves rolled up on the elbows), formal footwear, man scarf (we could add some tools to go to the fields to materialize this?) Outfit could be made from fabric with the logo of the campaign.

Caltu: Daughter of Hadha Caltu and Abba Caltu - The "dirty" puppet, aged 5 or 6 years old

Material: Baby female puppet

Start of Drama

Scene one: Dignity

Caltu's mother (Hadha Caltu) monologue to the audience. She tells the story of her encounter with her neighbour, Marga's mother (Hadha Marga) who told her unclean faces are not attractive or dignified and that faces and hands need to be washed with soap to maintain their dignity .

Hadha Caltu:

To the audience. Actress carrying the puppet, Caltu

Look at my child, doesn't she look pretty and sweet? She is, isn't she? **Pause**

But do you know what our neighbour, Hadha Marga told me yesterday? "Caltu does not have a Face of Dignity, and therefore none of your family do either." I was shocked: "Pardon, what are you talking about? What are you saying about her face and our family's dignity?" And she told me: "I mean your child's face is dirty so she doesn't have dignity or beauty and you do not have dignity because she reflects the whole family." I disagreed, my Caltu is clean, beautiful and dignified! Please, look at her… She only has a tiny bit of dirt on her face, this little dry discharge by her eyes and nose, but that is normal for a child. What is wrong with that? What's a face got to do with beauty and dignity anyway? Isn't that weird?

Caltu: **To the audience, focussing on the children**

No mummy! It's not like that, even this little bit of dirt makes me feel bad inside. I am too small to look after my dignity. As I am too little and don't know how to help myself, please teach and help me so that I learn to take care of my dignity.

Hadha Caltu:

Hmmm… wait… wait… wait… What is my baby saying? How does a tiny bit of dirt on her face make her feel bad? I don't understand.

Hadha Marga told me: "This tiny dirt, as you called it, is not good for your child and reflects badly on your whole family. Dignity is the starting point of everything. How can your family be dignified and respected in our community if your child is not clean? How can anyone look their most attractive without first addressing their hygiene?"

Of course, I know dignity is important, who of us would disagree with that?! But I told Hadha Marga that I just couldn't see how my family's dignity could be jeopardised by a tiny bit of dirt on Caltu's face.

Hadha Marga told me to close my eyes while she helped me to understand. Come, close your eyes with me for a brief moment. I will share with you what she told me.

Encourage audience to close eyes as you speak.

Dignity is a feeling.

[INSERT HERE SOMETHING THAT TELLS A STORY OF SOMETHING THAT HAS HAPPENED THAT WOULD MAKE THE AUDIENCE – including children (if possible) - FEEL UNDIGNIFIED].
How do you feel knowing that this has happened to you? Do you feel embarrassed, unimportant and ashamed?

Now, imagine the exact opposite story.
[INSERT THE OPPOSITE STORY, WHERE THE PERSON (or child) ENDS UP WITH THEIR DIGNITY ENTACT].
Do you feel the difference?

When we act with dignity, we feel peaceful, satisfied and good inside. This is displayed in our faces and how we walk and talk.
Even if we do not have hundreds of cattle or a huge arable land, when we have our dignity we feel rich inside.
If our faces are clean, they look their most attractive and are dignified. We can be at peace. We value ourselves and know we are important and worth caring about.

Male actor rings a bell quietly off stage after she says this.

So, that's it. It's about clean faces, beauty and dignity.
Hadha Marga made me feel so upset when she said my Caltu did not have a dignified face, but now I understand why Hadha Marga talked so much about dignity. It feels so good!

Hadha Caltu changes her position and looks to be talking to herself, thinking over something that makes her worry. Then she turns towards the audience, after a small break:

To the audience
My people, I was awake the whole night. Here I am without closing my eyes for a single minute. I was worrying about how I maintain my Caltu's dignity, how I ensure our family is seen to be dignified in the community and that we contribute to making our community dignified. What if my other neighbours think we do not care about ourselves or our dignity?
Can you believe that a simple thing like a tiny bit of dirt you can hardly see affects our children's dignity and reflects badly on our family and our community?

I wanted to make it clear in my mind! I went straight to Hadha Marga this morning to ask for advice. She told me not to worry, that there is a way to give our children Faces of Dignity and ensure the whole family lives a dignified life. What do you think she said?

Wait for audience to answer while Caltu acts out face washing
That's right, she said we all need to wash our faces and hands 3x a day, especially those of our pre-school children. She also told me that we should make sure we use soap each time we wash.

Scene 2: Face and hands washing removes discharge

Abba Caltu appears on the other side of the stage, coming back to the house. Caltu's mother (Hadha Caltu) runs towards him to tell him about her discussion with Marga's mother and they take the decision to wash Caltu's face with water and soap. Happy with the result, they sit to drink coffee.

Hadha Caltu:

The mother calls her husband loudly Abba Caltu…., Abba Caltu…?
Actor arrives on stage. Mother and father greet each other
Look! Look! Are you seeing anything which strikes you on Caltu's face

Abba Caltu:
Not really paying attention at first
Sorry? What? What are you talking about, she is perfectly fine, no?

Hadha Caltu:
Really? You do not see anything…?
Would you say she is a beautiful and dignified little girl?

Abba Caltu:
Taking time to think about it and looking at his daughter There is some dirt next to her eyes and her nose… let me wipe this dirt with my shirt.

Hadha Caltu:
Oh no! Hadha Marga told me that we shouldn't use cloth or rags to clean our children's face. She said we should wash ourselves and our children's face and hands three times a day. But if we have to wipe dirt from a child's face we should use our hands and wash them with soap right after.

Abba Caltu:
Oh, so we should avoid wiping her face and always wash our hands afterwards… Fine. Oh, look one fly keeps bothering her as well!

Hadha Caltu:
Chasing the fly from her daughter's face and shouting out. Jumps dramatically and tries to remove flies from her child's face. Should be funny.
I didn't notice the flies landing on my Caltu's face. Disgusting flies, ugh, I hate them! Ugh! It is disgusting to think that the flies that land on faeces are then coming to my child's eyes… I hate that! Bouuuh! I see now why Hadha Marga says Caltu can't be dignified with a dirty face… How can someone be dignified if they have faeces on their face?
She pauses
As if the idea of faeces on her face was not enough, Hadha Marga said that flies can also carry diseases like trachoma, an eye disease that can lead to a lot of eye pain and blindness. The good news is she said that the actions we take as parents and as a family can protect us all from this.

Caltu: *To the audience, focussing on interacting with the children*
I hate it so much when the flies are on me, don't you? And I can't do anything to get rid of them. Oh no! I don't want trachoma either that sounds horrible.

Hadha Caltu:
My husband, this is serious. I met Hadha Marga yesterday and she told me that our Caltu does not have a Face of Dignity and as a result our whole family is not dignified and is letting down the community.

Abba Caltu:
Looking really astonished. Oh! So what I have heard around the garee is about this then?

Hadha Caltu:
Alarmed What? What are you talking about? What have you heard around the garee?

Abba Caltu:
The Faces of Dignity! Every child is asking his friends about this! "Do you have a Face of Dignity?" Everyone is talking about that. Abba Marga told me about it as well… I was not sure what all this was about. He told me something about washing faces and removing dirt and beauty, dignity and respect in the community… Doesn't he have something better to think and talk about as a leader? He was expected to raise issues like our agriculture, building strong community through our religion and so on.

Hadha Caltu:
Cutting her husband talking Yes, yes, yes, it is all about this. How do you expect to have a strong community without starting first with dignity?
Hadha Marga told me we should wash our faces and hands with water and soap 3 times a day. And now, you are telling me that everyone in the community is already doing it and knows about it. Oh,

Abba Caltu, if we want our family to have Faces of Dignity we need to do the same! I usually use just water.

Caltu: **To the audience, focussing on interacting with the children**
I want a Face of Dignity, don't you all want a Face of Dignity?

Abba Caltu:
Pausing to consider his wife's plea. Looking to her and Caltu and agreeing
Yes, you are right. Dignity is most important. Abba Marga also explained to me that washing faces and hands with soap 3 times a day was important to prevent sickness. Washing with soap can prevent trachoma, diarrhoea and other illnesses too.

Hadha Caltu
To Abba Caltu
It is early morning still and Hadha Marga said the first time we need to wash is when we wake up. Let us wash and let me wash my Caltu, please hold her for me while I get the water.
Actress gives the puppet to the actor and goes to take water. Leaves stage

Abba Caltu:
Alone on stage with Caltu Caltu's nose is really stuffy… Oh! And all those flies… Having a Face of Dignity, is it all about this…?

Hadha Caltu:
**Back with jerry can, jug and collector. Starts to wash Caltu with water only. Abba Caltu uses the prop to highlight the face washing [TBD].*
To the audience Washing Caltu's face is an easy way to make her dignified and it makes her feel fresh and clean as well. I have done the right thing and that makes me feel good too!
Actress sings [INSERT THE DIGNITY SONG]

Caltu: **To the audience, focussing on interacting with the children**
Great! Mummy forgot to use the soap. Phew! I don't like soap when it gets in my eyes.
But wait… I don't feel clean and dignified like mummy said… I still feel dirty as if she didn't wash my face at all. Why did she bother to get the water and make me wet if I feel just the same? Maybe it isn't so great that mummy forgot the soap after all?

Hadha Caltu:
Ask to the audience
I don't feel as good as I thought. I have the impression I am forgetting something to help my Caltu be a dignified little girl… What am I forgetting? **Audience answers** Soap! Soap! Of course.

Abba Caltu:
I don't understand the whole story here Hadha Caltu, we always wash our faces every morning when we wake up from our sleep. So why are we expected to wash our face and hands with soap let alone the three times?

Hadha Caltu:
Yes, you are right my husband. What I am talking about is adding soap whenever we wash our face and hands so that our face keeps clean longer.
Abba Caltu, now come here and watch Caltu while I fetch the soap. I told you Abba Caltu, soap is essential! We need to remind each other until we get used to washing our face and hands with soap three times a day.
Gives Caltu to the actor and run comically to look for the soap

Abba Caltu:
Right, right, washing with water and soap… But the soap … what is the purpose exactly? You know I am not really keen on wasting soap, if…

Hadha Caltu:
Cutting her husband talking Wasting soap, wasting soap you say? What are you worried about huh? Is it about the money you spend to buy soap? How can you put a price on our children's dignity? Hadha Marga told me that it is the only way to get rid of all discharge, even the tiny ones, and to be truly attractive and dignified. If we do not use soap whenever we can, it is like not washing at all. And anyway, do you want our children not to be dignified when all the other children are washed with water and soap? And what about trachoma? If we forget to wash Caltu's face with soap the flies will come.

Caltu: **To the audience, focussing on the children**

I really want to be clean and dignified and not get sick. I wonder if it is worth not resisting the soap?

Abba Caltu:

Hadha Caltu! Are you mad? Are you really going to use soap and burn Caltu's eyes?

Hadha Caltu:

Enough, Abba Caltu, first you do not want me to use soap at all and now this! Do you want her and our family to be dignified? To get rid of the dirt, discharge, trachoma and the flies? Do you want us to be respected in the community…? Do you want us to stay healthy?

Abba Caltu:

Sheepish, hesitating Yes… Of course, yes… I want to be dignified and respected, and our family and… Caltu as well.

Hadha Caltu:

Good, so let me use soap. And do not worry, I am not stupid! This is a dedicated soap for body washing, it is not harsh. It will not hurt Caltu or make her skin dry. I will only use the right amount, and will not waste it.

Actress takes the puppet again and start to wash, describing in detail what she is doing. Actor makes some comments

Hadha Marga said I need to rub and wash well around the eyes and nose, let me try this. With the body soap and Caltu closing her eyes tightly, soap will not go into her eyes. And if Caltu is crying despite everything, I have another method to calm her down.

Actress sings a song to placate Caltu. Actor and actress do the actions for the song

[INSERT THE DIGNITY SONG]

Caltu: **To the audience, focussing on the children**

Actually, that wasn't too bad! I closed my eyes tightly the whole time mummy was washing my face and it was over quickly!

Now I feel fresh, clean and dignified, and I smell good too! I didn't feel like that without the soap. Don't you want to feel this good too?

Abba Caltu:

Great, you are done, let's drink coffee.

Hadha Caltu:

Not so fast Abba Caltu! We haven't finished yet. Look…

Actress turns to audience and says there is one more important thing that Hadha Marga taught her

Can you please turn to your family members and shake their hands for a minute? Don't let go of their hand until I tell you.

Actress insists until people in the audience shake their family members' hands. The actress continues to speak as people shake:

Abba Caltu, come, shake hands with me.

Abba Caltu should mime disgust as she talks very dramatically Imagine all the things this person you are shaking hands with could have been doing before shaking hands with you and imagine if you keep shaking hands with other people outside your family members. They didn't wash their hand before they shake yours so everything they have touched so far today is still on their hand. They might have cleaned up their child's faeces, cleaned snot from their child's face, cleaned the compound and thrown rotten food away.

Actress tells them to stop shaking How do you feel now? It's disgusting right?! Hands can look clean but they are not always! The dirt and discharge from their child's eyes are one of the many disgusting things that might have transferred to your hands when you were shaking hands. It is not just flies that can carry trachoma from eye to eye. Hands can do this too. In fact, a lot of diseases can be spread by dirty hands, like COVID-19 and diarrhoea. Giving each other diseases is not a very dignified way to behave and is not how we look after one another in the community. We will give you soap before you go home today so you can wash faces and hands directly when you get home.

So you see, I should not forget to wash Caltu's hands whenever I wash her face!

Actress mimes washing the puppets hands with soap and the couple talk about how it is easy for them to wash their hands when they wash their faces because the soap is on their hands when they wash their face, but they need to wash Caltu's hands for her as they do not otherwise get soap on them.

Caltu: **To the audience, focussing on the children**
I am so relieved that mummy has washed my hands properly, I am touching so many hands everyday! I don't want to faeces, diseases or anything from the other people!

Abba Caltu:
Waouh! Look Caltu is quiet now and a beautiful and dignified little girl. We know she is dignified and that is what is most important. Now I see what the "Faces of Dignity" Campaign is all about.

Hadha Caltu:
Yes, it is what Hadha Marga told me, from the "Faces of Dignity" Campaign. It must be important because [INSERT NAMES OF LOCAL COMMUNITY, RELIGIOUS AND CLAN LEADERS] as well as the Health Volunteers and Health Extension Workers are supporting this.
[If any of these people are in the audience, ask them to stand briefly and agree]

Abba Caltu:
That is really impressive. Previously, the reason why I was complaining about using soap was trying to reduce our financial expenses… But I get it now, it is all about having Faces of Dignity, being a dignified family and being a dignified community. Between our family's dignity, our Caltu's dignity and the money for soap, the 10ETB for the soap is no hard choice. Dignity should always come first. It is the most important thing we can do for our children. I see now that our children's dignity is also important. They are representing our family in the community all the time, as soon as they can run out to our neighbours' homes. I would hate to think people are thinking badly about our family or that we do not represent our community well. Hadha Caltu, we will buy soap and wash as a family. We will wash Caltu's face and hands with water and soap as she is too young to take care of her own dignity.

Hadha Caltu:
You are right, that is a good decision. I am going to prepare coffee to celebrate our dignified family.
**Actress goes to look for the coffee material while the actor is staying with Caltu, humming a song and playing with her, joking about her Face of Dignity*.*

Abba Caltu:
To the puppet Who has a nice *Face of Dignity*? My Caltu, yes, my Caltu!

Caltu: **To the audience, focussing on the children**
At first, I hated the idea of soap and cold water on my face, but now I know that it makes me feel so good, so refreshed, clean and dignified. I am so lucky my mummy and daddy love me so much to take such good care of me and to make sure we always have soap for washing.

Hadha Caltu comes back and prepare coffee. They drink coffee together, Caltu playing on her father's arms

---

Scene 3: Washing three times a day with soap

Hadha Caltu and Abba Caltu are satisfied with how they have washed Caltu's face. But, they suddenly realise that Caltu's face is dirty again. Thanks to Hadha Marga's advice, they come to the conclusion that they should wash Caltu's face 3x a day!

Caltu: **To the audience, focussing on the children**
I have been running around and I don't feel clean and dignified anymore. My parents don't seem to be aware or doing anything about it. How do I get their attention? I know, I must cry…

Abba Caltu:
While drinking coffee, Abba Caltu realises that his daughter is starting to cry and feel discomfort again
Hadha Caltu, look, it has only been a few hours but our daughter does not seem good again… She is crying.

Hadha Caltu:

Oh! She has a stuffy nose. Look at her eyes, there is discharge as well. I didn't think she could get dirty again this quickly!

Abba Caltu:

Oh! Again! Already?

Hadha Caltu:

Shall I keep washing her all day? Isn't once enough? Let me go to Hadha Marga, maybe she has heard a solution for that from the Faces of Dignity Campaign or the Health Volunteer.

**Actress is leaving the stage to get some advice and leaves Caltu with the actor*.*

Abba Caltu:

**Alone. Actor is complaining about his daughter crying. He tries to calm her down, humming and saying nice words. Shortly, Hadha Caltu comes back*.*

Caltu: **To the audience, focussing on the children**

I don't understand. Mummy washed my face this morning, it is only lunch time and I feel dirty and have sticky eyes again.

Hadha Caltu:

I have told the whole story to Hadha Marga. I said that we washed Caltu's face and hands with soap this morning but it seems like her face needs washing again. She reminded me, that washing only once a day in the morning is not enough because dirt and discharge comes back again and the face loses her beauty and dignity. She said that to keep the dirt away and prevent trachoma we should wash three times each day - in the morning when we wake up, but also before lunch and before dinner when we wash our hands, and always with soap. Hands need to be washed whenever faces are washed as we touch our faces so much! She said that this is important so we all have Faces of Dignity and live dignified lives.

Abba Caltu:

In disbelief How many times a day, have you just said?

Hadha Caltu:

Three times a day, throughout the day, and with soap! Let me go and get the water and soap. We will eat lunch soon and we will wash our hands, it will be easy to wash our faces then too.

Actress leaves the stage to get the wash material, including soap

**Starts*

Abba Caltu:

**Actor alone, holding Caltu, asking his child where her Face of Dignity has gone*.*

Oh my Caltu, it seems unnecessary to wash this much, what can be the benefit?

Caltu: **To the audience, focussing on the children**

Oh Daddy, don't say that! I feel so fresh and good inside and out after my face is washed with soap but that feeling doesn't last the whole day!

Hadha Caltu:

**Actress comes back and starts washing Caltu's face and hands again*.*

[INSERT THE DIGNITY SONG]

Here we go again, what are you thinking Abba Caltu? Clean is always beautiful and dignified. To get truly clean we need to rub very well around the eyes and nose.

So we need to remember, if sometimes I am not at home, in the morning, before lunch or before dinner, please remember to wash Caltu's face and hands, on time, with water and soap. Even though her sister is in school we should not forget to remind her as well. In fact, we need to do it together as a family so we can help each other and set a good example for the children.

Abba Caltu:

Aha! Now you put it like that I understand fully. Caltu's beauty comes from her dignity, and dignity starts with a clean face. I will also make sure there is always soap for her. She is the face of our family. If we want to be dignified she has to be dignified first. She is going to be the priority now.

Hadha Caltu:

Good. I am sure she will get used to face washing if we do it three times a day. Soon she will not cry anymore. Even if she cries, I will continue because I am her mother and I know what is best for her. I will remind her to keep her eyes tightly closed so the soap does not sting her eyes. We must all wash, the whole family! We must also wash hands whenever we wash faces. These behaviours will set a good example for her and will make sure our family is dignified.

Abba Caltu:

You are right and I will buy her a dedicated soap for face washing as well so it is always at home when it is needed. Great! Let me go to the fields now.

Actor leaves the stages. Actress alone with Caltu

Scene 4: Becoming Faces of Dignity role models in the community

---

Hadha Caltu and Abba Caltu are recognised as role models in the community now they are washing Caltu's face (and hands) three times a day with soap. It makes them feel good!

Hadha Caltu:

Alone on stage. Hugging Caltu and repeating for herself

I wash my Caltu's face. I use soap. I rub well around her eyes and nose so I remove dirt, discharge, flies and disease. Three times a day, in the morning before breakfast, before lunch and before dinner. I will do that for my Caltu so everyone wants to play with her and she has a *Face of Dignity* in the community and is protected from trachoma….

Hadha Caltu is interrupted by her husband coming back from the field… he is carrying a wash station and soap in a soap dish which he puts down on a table as he enters

Abba Caltu:

Interrupting Hadha Caltu's monologue, putting his arm to the sky and screaming loudly and happily

Bravo! Bravo! *(To be translated: moral)*

Hadha Caltu:

What is that?

Abba Caltu:

Bravo! Bravo! Oh, Hadha Caltu! I was outside, working in the fields, when Abba Marga and Abba Kedir came to congratulate me. I was so surprised, so I asked the reason for such nice words. Then, they told me: "Your wife has become a role model for this community since she is washing Caltu's face three times a day with soap. Your family's Faces of Dignity are known to everyone." My wife, bravo! Bravo!

Hadha Caltu:

Actress now smiling and looking happy Oh my dear husband, there is always something which keeps telling me that you love me. That is true, I know that you love me my husband. But, don't you think you are bragging everywhere?

Abba Caltu:

Yes of course my wife, I am bragging! I was so happy when my friends told me that we are considered as role models in our community now. I brag because they told me that now we have Faces of Dignity, we are respected by the whole community. I will continue to do the same because they told me that our name is being given as an example of a dignified family. People are talking about us, saying good things about how you are maintaining our children's dignity and also protecting them from getting trachoma. It is great that we prioritise little Caltu and wash her face first as she is running everywhere representing our family. And they said to me: Bravo! Bravo! That's why I am also saying Bravo! Bravo!

Hadha Caltu:

To the audience I have changed and feel peaceful and better inside because I know that my family leads a dignified life. Dignity is so important don't you think? It is priceless. I expect that you will be telling your neighbours who could not attend this event all about how they can make sure their family has Faces of Dignity, maybe at your next coffee ceremony or next garee meeting? You see, Hadha Marga taught me about this, so I am telling you. In my opinion you should now do the same and tell others. Let's show everyone that we live a dignified life here. It's so simple: just wash your children's faces with soap three times each day! Wash their hands when you wash their faces!

Abba Caltu:

Talking to his wife Don't forget the 3 times! They should wash in the morning before breakfast, before lunch and before dinner. The whole family, but always children first! They will all have Faces of Dignity and feel good inside as well as look good outside.
Goes over to the wash station Look here what I have brought for our family. A wash station! We can make sure we continue as we have started by using this wash station and keeping soap clean in this dish so it is always available for face and hand washing. Using this wash station will make face washing so much easier. There will always be water available and we can use the tap to control how much water we are using. We will make sure we always fills it with water when we come back from collecting water and keep the soap in the soap dish next to it. Just seeing this wash station will help us always remember!
To the audience Raise your hand if you would like one of these to help you remember to wash faces and hands? Then your family can also show that they live a dignified life!

Oh, it's already getting dark so let's wash our face and hands before we get to our dinner. *Picking Caltu in his hands* Come on my precious child, you have no idea how you made me feel proud and dignified. Who would make me think about all these excellent ideas of protecting personal hygiene if you were not here?
Talking to his wife
And my wonderful wife it is your effort that made our family dignified, clean and healthy. Come join me and our Caltu to wash our face and hands.
The father helps Caltu to wash face and hands using the station, then wash his own face and hands. The mother washes her face and hands as well.

Caltu: *To the audience, focussing on the children*
I am used to washing my face and hands with soap 3 times a day, morning, before lunch and before my evening meal at the nice, new wash station. It is normal for my family and we all wash together whenever we can. The rest of our community is doing it too! When my face is washed with soap, I feel good inside and so proud to be a member of such a dignified family! Now this is our habit we will always do it!

End of the Drama
