# Supplementary material for: Cluster randomised controlled trial of double-dose azithromycin mass drug administration, facial cleanliness and fly control measures for trachoma control in Oromia, Ethiopia: the stronger SAFE trial protocol
Source: BMJ Open. 2024 Dec 23;14(12):e084478. doi: 10.1136/bmjopen-2024-084478 (PMC11751794; doi:10.1136/bmjopen-2024-084478)
Supplement: online supplemental file 3 [file bmjopen-14-12-s003.pdf]

EVENT 2 – FAMILY FORUM 1
ACTIVATOR MANUAL

**Purpose**	This event is designed to build washing related knowledge, skills, and motivation, and empower households to construct wash stations to aid habit formation.
**Responsible parties**	1 trained Activator + 1 trained Health Volunteer (2 HVs will assist the Activator in each cluster to spread the work load)
**Participants**	All household members living within the 'yolk' of an intervention cluster who attended the Community Event – Split into groups of 5 households.
**Location**	A HH compound (selected in advance when HHs are informed the time and date for the forum)
**Duration**	1h30
**Timing**	Workdays (except Friday morning), 9am to 1pm and after 3pm

Preparation

Day before the event

- Mobilise HVs to recruit 5 neighbouring households living in the 'yolk' of the intervention cluster according to the census list. If 1-to-5 Groups ('ijaarsa olla') exist in the cluster, they should serve as a basis to form groups of 5 neighbouring households. Otherwise, grouping of households will be done according to geographic location. As much as possible, households will be grouped considering any local sensitivities. Groups can range from 4 to 6 HHs to adapt to local circumstances.
- HV to ask one household to host the event. If 1-to-5 Groups exist in the community, select the house of the 1-to-5 Group Leader (if the compound has space to hold the event).
- Communicate time and location of the event to each participant household.
- Inform each household that the whole family should participate and that they will receive some gifts if they stay for the whole event.

Setting

The sketch below illustrates the ideal setting for the forum. Activators are free to amend the set-up according to each HH setting. Any setting should respect the following criteria as much as possible:
- Participants should be able to see each activity and the flipchart easily.
- Participants should be sat in the shade of a tree or of the house, under a canopy whenever possible.
- Materials for further activities should be well-organised and kept out of the way but easily accessible.
- Participants should sit with their household members and observe a physical distance of at least 2m with other households.

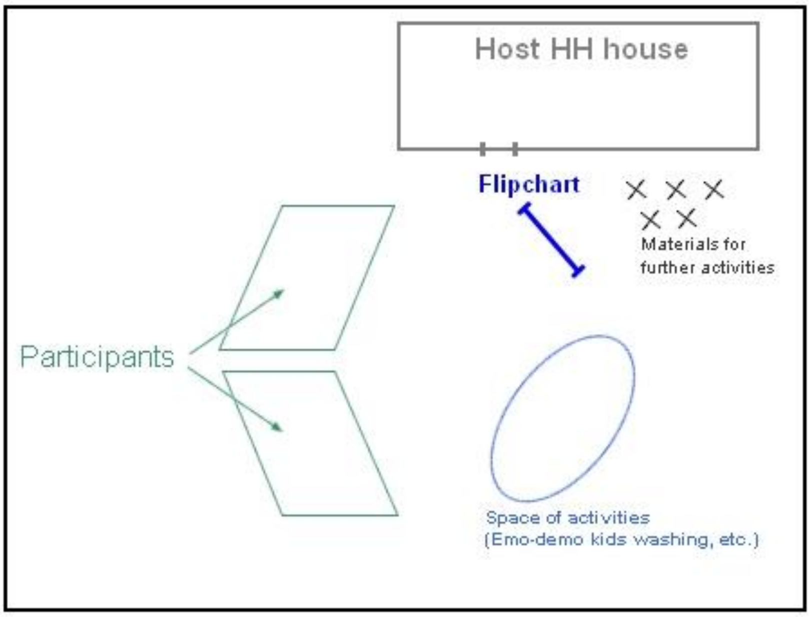

Materials

General

- COVID-19 preventive materials: cleaning solution in a spray (x1), alcohol-based sanitiser, facemasks (for activator and HV), sealable plastic bag to dispose masks
- Flipchart
- Caltu's puppet in its cover
- Pen
- Notebook
- Cell phone or device to play the Dignity Song and the recorded Testimonials
- Ask the hosting household to borrow a stool or a chair for installing the wash station for the emo-demo

Trachoma Transmission Routes

- Transmission board (magnetic board and laminated sheet)
- Paper clips (x4)
- 9 magnets: fly magnets (x2), discharge magnets (x4), hand magnets (x2), scarf magnet (x1)

Face Wipe Emo-Demo

- Moisturizing cream + Foundation powder with a brush
- Green clay for face
- Face wipes (x16)
- Branded wash station borrowed from the HV (if possible, filled with water) + if possible, branded wash station borrowed from the host HH
- 20L jerrycan full of water
- Water collector
- 14 paper clips
- 2 laminated sheets of white paper to present the wipes
- 2 'Washing protocol sheets: Water only and Water and Soap' to present the wipes

Wash stations

- 15 drawings of the puppet to be coloured in
- Wax crayons (x15) *(NB. Crayons will be taken back from the children at the end of Family Forum 1. Children will keep their wax crayon at the end of Family Forum 2)*

Testimonials

- Activator's phone with the recorded testimonials on it

Dignified Day Pledge

- 5 Dignified Day posters
- 10 small nails

Set up

Day of the family forum

- Do you have all materials required for all activities?
- Is equipment working?
  Tablets have full batteries with testimonials for that cluster on the tablets.
- Has the Activator put cream and foundation powder on his/her face (and washed their hands afterwards)?
- Has the flipchart been placed in a suitable location?

Activities

Activity 1: Introduction

Purpose
- To provide introduction to the forum.
- To address any concerns or questions arising from the Community Event.

What to do

1. Install the flipchart – Family Forum 1 Cover image. Install Caltu's puppet next to the flipchart on its cover.
2. Play the Dignity Song on a cell phone or any other device while participants are arriving.
3. Advice community members to sit with their household members and respect a physical distance of at least 2m with other households.
4. Greet participants and welcome them to the forum.
5. Complete attendance sheet.
6. Introduce yourselves and remind the group that they saw you or your colleagues at the Community Event.
7. Ask participants to raise their hands if they went to the Community Event.
8. Choose one participant who came to the Community Event to summarise its content and key messages for participants who were not present.
9. Ask if anyone has any questions about anything they heard at the Community Event. Answer their questions before proceeding.
10. Say that they have already started to learn how to have *Faces of Dignity*, and that we will continue this learning today.
11. Tell participants that the event should not take more than 1h30. Say that they will be given some small gifts for the entire family, so it is very important they stay until the end if they can.

Activity 2: Trachoma Transmission Routes

Purpose
- To understand how trachoma is transmitted, i.e. flies, fingers and fomites.
- To understand regular and thorough hand and face washing with soap reduces the risk of disease.
- To perceive discharge as disgusting and dangerous to health.

What to do

1. Turn the flipchart – Trachoma Transmission Routes page.
2. Ask participants what they learnt about how trachoma can get from one eye to another eye at the Community Event. Correct participants if you need to and congratulate them on their answers (they should mention flies and hands).
3. Set up the Transmission Board (i.e. fixed the laminated sheet on the magnetic board using the paper clips) and explain that we are going to talk a little more about transmission so that everyone understands why it is so important to get rid of discharge.

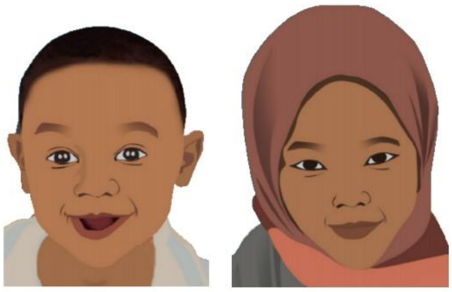

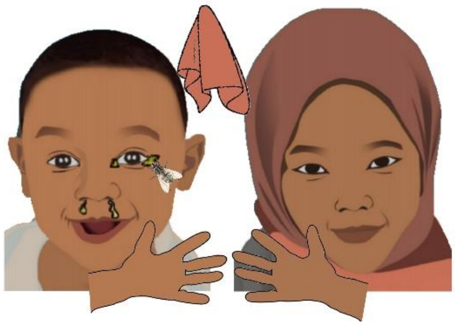

Trachoma is carried in discharge

1. Tell participants that trachoma is a small germ and is carried in discharge that we can often see on people's faces.
2. Ask some children to come and show on the Transmission Board where they usually see discharge on someone's face. They should show discharge below the eyes and nose. Help them if you need to. Give them 4 discharge magnets to put under the boy's eyes and nose.
3. Ask participants whether they agree that discharge carrying trachoma can be found under the eyes and the nose of children.

> **Demonstrate using the board**
> 1. Put/Move the magnetic discharge under the eyes and the nose of the boy's face and explain what you are doing.

First transmission route: Flies

1. Introduce the first route of transmission, flies.
2. Ask some children to come and put the 2 fly magnets on the boy's face on the Transmission Board. They should show flies under the eyes and nose. Help them if they need it. *Invite the children (or their caregivers if the children are too young) to assist with the rest of the demonstrations by moving the magnets when you ask them to. Make sure the rest of the participants can see the board.*
3. Ask participants whether they agree that flies land under children's eyes and noses.

> **Demonstrate using the board**
> 1. **"A fly lands on this discharge under the boy's eyes because it likes to eat it."** – Make magnetic fly travel to the boy's eye.
> 2. **"Discharge from the eye gets on the fly."** – Pick up the discharge magnet using the fly magnet.
> 3. **"The fly then lands on another child's eyes."** – Make the magnetic fly travel towards the girl's face and put the fly next to the clean eye.
> 4. **"Guess what happens?"** – Let people answer. **"Yes, when the fly lands on the girl's face the discharge rubs off the fly onto the girl's face. Her eye will become dirty as well." –** Take the discharge off the fly and put it under the girl's eye.

4. Ask participants whether they agree that flies can transmit discharge carrying trachoma.

Second transmission route: Hands

1. Move the discharge from the girl back to the boy.

2. Inform participants that we are now going to look at a different way discharge can spread from the boy's eye to the girl's eye: hands.

> **Demonstrate using the board and with the help of any children in the group.**
> 1. **"Now imagine those two children are playing together. One has discharge and the other one is clean. While playing, the boy touches his dirty eyes or nose."** – Lift the magnetic hand and place it on the boy's face so it picks up the discharge magnet.
> 2. **"His hands become dirty and now carry the discharge with trachoma."** – Show the dirty hand (with the discharge magnet) to the crowd.
> 3. **"While playing, the boy will certainly touch the hand, or even the face of the girl."** – The two activators put the hands in contact and make sure that the discharge magnet moves onto the second hand.
> 4. **"Then, what's going to happen?"** **Let people answer** **"The discharge will move from the boy to the girl, who is likely to touch her face at some point."** – Show the discharge on the hand of the girl to the crowd (show that the magnet has changed hand).
> 5. **"Eventually, she will put the discharge with trachoma on her own face and get dirty as well."** – Make the girl touch her face and move the magnetic discharge to under the eyes.

3. Ask participants whether they agree that hands touching dirty faces can also transmit trachoma.
4. Mention that caregivers can also easily transmit trachoma between their children if they touch them one after another, notably when they are wiping their children's faces to remove discharge.
    a. Advise caregivers to always wash their hands with soap directly after wiping a child's face to remove discharge from their hands.

Third transmission route: Clothing

1. Move the discharge from the girl back to the boy.
2. Inform participants that we are now going to look at one more way that discharge can spread from the boy's eye to the girl's eye: our clothes.

> **Demonstrate using the board**
> 1. **"Now imagine that these two children are siblings. One has discharge and the other one is clean. Their mother comes and sees discharge on her boy's face. She uses her scarf to remove his nasal discharge."** – Use the piece of cloth/a scarf magnet to "wipe" the discharge under the boy's nose. Attach the magnetic nasal discharge to the scarf.
> 2. **"The scarf of the mother becomes dirty and now carry the discharge with trachoma."** – Show the dirty scarf (with the nasal discharge magnet) to the crowd.
> 3. **"Later on, the mother will certainly use her scarf to touch the hand or the face of her daughter."** – The activator puts the scarf next to the eye of the girl.
> 4. **"Then, what's going to happen?"** – Let people answer. **"The discharge will move from the scarf to the girl's face."** – Put the nasal discharge magnet from the scarf under the girl's eye.

3. Ask participants whether they agree that cleaning discharge using clothing can also transmit trachoma.
    a. Advise caregivers to always use their hands to wipe discharge on their children's face and avoid using a cloth or a scarf. Remind caregivers that they should always wash their hands with soap directly after wiping their children's faces to remove discharge from their hands.
4. Ask participants to summarise the 3 ways they have learnt about how trachoma can be transmitted. – *Let people answer. Point to the props (fly, hand and scarf) as each transmission route is mentioned.*
5. Congratulate the participants on their understanding.

Face washing to limit transmission

1. Ask participants what they can do to get rid of the trachoma-carrying discharge and maintain their children's dignity. They should answer face (and hand) washing. If not, probe for what was done in the community event.
2. Ask participants to raise their hands if they agree that they can wash their children's faces to get rid of disgusting discharge and maintain dignity.
3. Tell participants that faces and hands need to be thoroughly washed to get rid of discharge, especially around the eyes and nose. Pre-school children need to be helped so they can wash well. Caregivers should wash their hands with water and soap when supporting their children and each time they are wiping their children's faces.
4. "Wash the children's faces" on the Transmission Board using water and soap by removing the magnetic discharge.
5. Spray the Transmission board and all magnets with cleaning solution.

Introduce the Face Wipe Emo-Demo

1. Ask participants if they think that they are always able to see all the dirt and discharge on a person's face.
2. Tell the group that this will be the topic of the next activity, and that some discharge is not easy to see but it can still carry trachoma.

ACTIVITY 3: FACE WIPE EMO-DEMO

Purpose

- To perceive discharge as disgusting and dangerous to health.
- To understand germs on hands and faces (particularly of children) are invisible and cause trachoma.
- To understand that to achieve truly clean hands and faces, soap must be used.
- To understand regular and thorough hand and face washing with soap reduces the risk of disease.
- To perceive soap as important to use each time faces are washed.
- To perceive effectively washing face at least 3x per day as important, all year around.

What to do

1. Turn the flipchart – FACE WIPE EMO-DEMO page.

Note for the activators

The Activator should put foundation on their face before the forum.
The HV should put some dirt on his/her face at the start of this activity.
Both should have washed their hands with water and soap before starting.

Demonstration 1: A clean face is not a clean face!

Activators' demonstration

1. Ask participants/children if they see any difference between the face of the Activator and the face of the HV.
2. Ask children to point towards the person with the dirty face.
3. Explain to participants that you are going to use wipes to confirm what they have just said. Explain that the wipe is just a cloth that is a bit wet.
4. <u>Wash your hands with soap before starting</u>.
5. Ask participants which wipe they expect to be dirty.
6. The activator and HV should <u>wipe the</u> <u>RIGHT side of their faces</u> and pin the wipes onto a laminated piece of white A4 paper
7. Ask the audience what they see: both wipes are dirty. Is this a surprise?
8. Say that faces can look clean even when they are not.
9. Ask the audience if they agree with this.

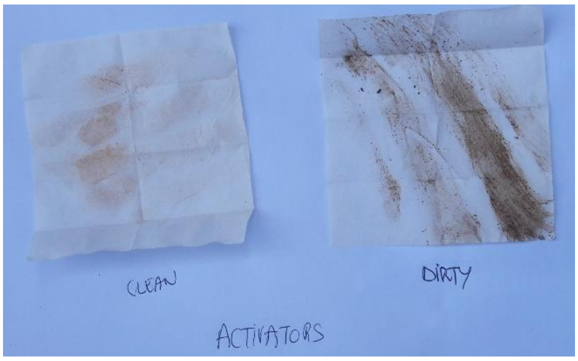

Children's demonstration

1. Explain to participants that this is true in their community as well, even for children who seem to have clean faces.
2. Put facemasks on and explain to participants why you are doing this because you will get near to their children.
3. <u>Select up to 4 to 6 children</u> aged approximately <u>6 to 9 years</u> old.
4. Explain that you will repeat the activity to see if there is any invisible dirt or discharge on them as well.
5. <u>Turn away from the crowd</u> (so they cannot see which child is the dirtiest) and use a clean wipe to <u>wipe the RIGHT side of each child's face</u>.
6. Remember which wipe comes from which child, but do not share this with the group.
7. Clip each used wipe onto the laminated piece of white paper and show the dirty wipes to the group.
8. Ask them again if they agree that a face can look clean but it is not necessarily clean and therefore is not a face of dignity and may also carry trachoma.

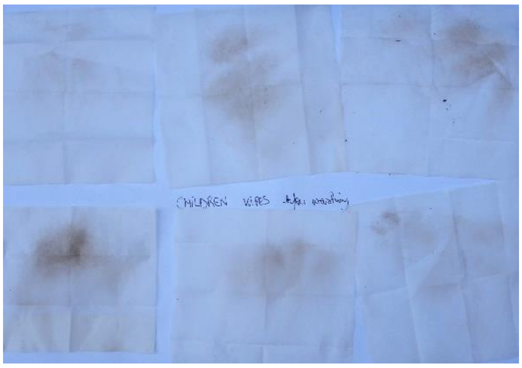

NB. It is unlikely but possible that all children's wipes will be really clean/white. If this happens congratulate the children and parents on their Faces of Dignity and do Demonstration 2 on yourselves and not the children (the Activator with foundation should wash with water only, while the HV with obvious dirt washes with water and soap).

Demonstration 2: A face is clean and dignified only if we use soap!

1. Ask participants what they could do to make sure their children's faces are never dirty and undignified when they appear clean.
2. Tell participants you will do another experiment to show them how children's faces should be washed. Say that half the children will be washed with water and the other half with water and soap, because these are the different ways that we wash our faces.
3. Set the wash station on a stool borrowed to the hosting household and place a water collector on the ground under the tap. Place the soap in the soap dish on top of the station. (If possible, ask the hosting household to borrow their wash station to speed up the face washing process).
4. Ask the children with the dirtiest face wipes from the previous exercise to wash their faces with water, while the other children wash with water and soap. Encourage them to turn the tap of whilst they lather the soap.
5. Wait for the children's faces to dry (wait a minute or two).
6. Turn away from the crowd with the children who washed with water and wipe the LEFT side of each child's face thoroughly (including near the ear / side of neck) with a clean wipe.
7. Clip the wipes onto the laminated "Water only washing protocol" paper.
8. Repeat with the children who washed with water and soap. This time take care not to wipe the face as thoroughly, the wipes should appear clean!
9. Present the wipes to the group on the laminated "Water and soap washing protocol" paper.
10. Ask participants to explain what they see. They should conclude that faces are not clean unless they are washed with soap.
11. Remind participants that soap is required for true cleanliness and dignity.
12. Ask participants to raise their hands if they
    a. Agree that soap is the only way to achieve *Faces of Dignity*.
    b. Value their dignity and the dignity of their children.
13. Ask the group if they want to wash faces with soap regularly so their whole family have *Faces of Dignity*.
14. Conclude this activity by reminding participants that:
    a. A face might look clean but can be dirty anyway (point at appropriate wipes).
    b. Dirt and discharge, as demonstrated in the transmission activity, visible or not, can carry trachoma.
    c. Only a face washed with water and soap is a true "face of dignity", i.e. a face which is really clean (point at appropriate wipes).
15. Safely dispose your facemasks in a sealable plastic bag.

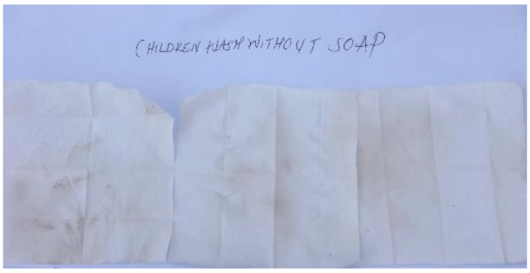

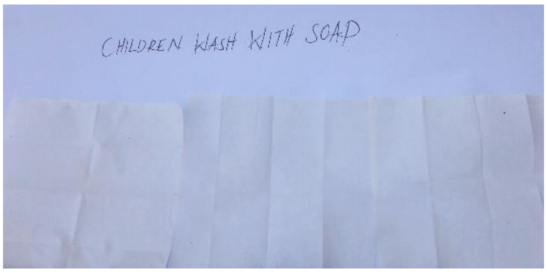

ACTIVITY 4: WASH STATIONS

Purpose

- Can construct a functional wash station.
- Functioning wash station is consistently available and accessible.
- Soap is consistently available and accessible for washing.
- Water storage, soap storage and wash equipment are consistently available and accessible.

What to do

1. Turn the flipchart – WASH STATIONS page.
2. Before starting the Wash Station activity, propose to children (aged 2 to 7 or 8) to colour in a drawing of the puppet explaining about face washing. Tell the children they will be able to keep their drawing to remind them about when they should wash their faces to become *Faces of Dignity*. Put the puppet in a visible place. Give one drawing to each child and a few wax crayons disinfected with the cleaning solution.
3. Introduce the wash station activity:

> **To keep our *Faces of Dignity*, we have just seen that we need to wash our faces, especially our eyes and noses, with water and soap. In the community event, we saw that it is not always easy to gather all the materials to wash – sometimes it can be hard to find soap when we need it. It is hard to keep the soap clean while we wash. It is also easy to forget to wash or to use soap. Here (point to it) you can see a wash station like the one that you got just after the Community Event.**

4. Bring forward the wash station and the soap in a soap dish used in the Face Wipe Emo-Demo (keep it on the stool if possible, so everyone can see the station properly).
5. Ask participants what they like about using a wash station (e.g. when they use them in markets and restaurants) and discuss whether a wash station makes it easier to wash faces and hands with soap. Mention that keeping the soap in the soap dish at the wash station means that a dedicated soap is clean and available for body washing when it is needed.
6. Say that we will now discuss how to construct a stand for the wash station they received at the Community Event and how the station should be used.

Location and Structure

1. Turn the flipchart – WASH STATION 'LOCATION' images.
2. Ask the group to describe the images.
3. Turn the flipchart -– WASH STATION 'STRUCTURE' images.
4. Ask the group to describe the images
5. **Summarise: a 'good' location to keep the station is outside, close to the home but in the shade. The wash station and soap dish are on a built, sturdy wooden structure that is secured and cannot be knocked over by children or animals.**
6. Ask participants if they have any suggestions or concerns about the location or structure. Reassure them that building a wooden structure does not take that long. In neighbouring communities, people have on average spent two hours collecting wood and building the wash station stand.
7. **Guide the discussion** to cover the following potential concerns:
    a. Outside location: because most face washing takes place outside.
    b. Shady location: to protect the wash station container from damage. Could build a roof if area near the house is exposed.

Height

1. Turn the flipchart – WASH STATION 'HEIGHT' images.
2. Ask the group to describe the images.
3. **Summarise: very young children cannot reach the tap and soap without help, but the station should be accessible to all other family members**.
4. Ask participants if they have any suggestions or concerns about the height of the structure for the wash station.
5. **Guide the discussion** to cover the following potential concerns:
    a. Accessibility: which age children should be able to access the station on their own? Who should be prevented from accessing the tap or soap? Who will help them wash?
        i. Is there anyone else in the family who needs to be thought of? e.g. disabled.

Drainage

1. Turn the flipchart – WASH STATION 'DRAINAGE' images.
2. Ask the group to describe the images.
3. **Summarise: a bowl or stones/sand are placed under the tap to prevent the ground from becoming muddy.**
4. Ask participants if they have any suggestions or concerns about drainage for the wash station.
5. **Guide the discussion** to cover the following potential concerns:
    - Drainage vs allocating a dedicated water collector.
    - Avoiding 'splash back' from a collector.

Water

1. Turn the flipchart – WASH STATION 'WATER' images.
2. Ask the group to describe the images.
3. **Summarise: someone is responsible for filling the wash station container with water so there is always water available for use.**
4. Ask participants if they have any suggestions or concerns about keeping water ready for use at the wash station.
5. **Guide the discussion** to cover the following potential concerns:
    - Filling the wash station container: how can they make sure they always have water for washing? Who will be responsible for filling the wash station container? When will they do this during the day? Suggest collecting more water and dedicating some water to the wash station each time water is collected.
    - Not taking the wash station container to the water point: carrying it can break the tap.
    - Avoiding wastage: close the tap whilst lathering hands or whilst rubbing soap on faces to avoid wasting water.

Soap

1. Turn the flipchart – WASH STATION 'SOAP' images.
2. Ask the group to describe the images.
3. **Summarise: a dedicated soap is kept in a soap dish at the wash station.**
4. Ask participants if they have any suggestions or concerns about keeping soap at the wash station.
5. **Guide the discussion** to cover the following potential concerns:
    a. Ability to dedicate soap and keep it at the wash station: put the soap we gave them at the community event in the soap dish as it protects the soap from rain

and getting dirty. Could bring soap inside at night along with the wash station container and take it out again in the morning.

  b. Lack of soap: Could also make soapy water and keep it at the wash station.
  c. Children wasting soap: young children could be supervised?

Night

1. Turn the flipchart – WASH STATION 'NIGHT' image.
2. Ask the group to describe the image.
3. **Summarise: at night, if there is no fence around the station, it might be preferable to take the wash station container and the soap and the soap dish inside the hous**e. **Someone should be responsible for bringing the station, soap dish and soap outside every morning.**
4. Ask participants if they have any suggestions or concerns about bringing their materials inside during the night and outside during the day.
5. Guide the discussion to cover the following potential concerns:
   a. Security: The wash station container could be secured outside or brought inside at the end of the day.
   b. Bringing it back outside in the morning: If people want to bring it inside, who will be responsible for moving it each morning and evening? E.g. the person who wakes first in the morning could bring the materials outside when they wash.

Complete station

1. Turn the flipchart – WASH STATION 'COMPLETE' image.
2. Say that this image summarises all the points you have just discussed about wash stations. Go through each point.
3. Ask participants whether they have any other questions or concerns about how to set up their wash station when they get it home.
4. Remind participants that all this information is summarised on the wash station flyer they received at the distribution. Suggest that they use the flyer to remember the important features of the wash station stand. Suggest that neighbours help each other.

Introduce the Testimonials

1. Tell participants that we will now show them some ways members of their own community have built their stations and have overcome barriers to use their wash station to help their family maintain *Faces of Dignity.*

ACTIVITY 5: TESTIMONIALS

Purpose

- Learn from neighbours' and role models' experiences about building, using and maintaining a face washing station.
- Functioning wash station is consistently available and accessible.
- Perceive the wash station to be useful.
- Create the impression that important community members are already using wash stations to practice the target behaviour.

What to do

1. Turn the flipchart – TESTIMONIALS page.
2. Start playing the voice-recorded Testimonials on a tablet or cell phone. Ensure everything can hear what is being said. If requested by the participants, play the Testimonials again. For content of the Testimonials, **refer to Appendix A**.
3. Discuss the testimonials briefly with the participants.
4. Ask if anyone has any comments on anything they have heard. Answer any questions.

5. Ask participants, especially fathers, if they now feel they have all the support and the information they need to set up a wash station to make it easier for their family to wash their faces 3x a day with soap and to maintain their *Faces of Dignity*.
6. Specify that:
    a. We advise them to put the body soap they got at the Community Event inside the soap dish they will be given at the end of this event.
    b. This is a dedicated soap for face washing which should be used at the station. If it has already run out, suggest that they buy more soap when they can.
    c. Next time we meet we will discuss how to make soapy water so that soap lasts even longer.
7. Conclude the activity informing participants that they will be asked to give their own testimonials at our next forum a week from now.

NB. This activity will be refined during pilot-testing. Testimonials might be video or audio-recorded according to what is feasible. If recording Testimonials is not feasible, this activity might also be dropped from Family Forum 1.

ACTIVITY 6: DIGNIFIED DAY PLEDGE

Purpose

- Engaging, accessible, appropriate, strategically placed washing prompts/cues are visible.
- Perceive face washing to be important for maintaining dignity of self and family.
- Perceive soap as important to use each time faces are washed.
- Perceive effectively washing face at least three times per day as important, all year around.
- Accept responsibility for hygiene of young children.
- Perceive an expectation from husbands & neighbours to maintain clean faces of self and family.
- Perceive an expectation from parents to maintain clean face.

What to do

1. Turn the flipchart – DIGNIFIED DAY PLEDGE page.
2. Before starting the Dignified Day activity, ask children to join back the forum. Congratulate children on their drawings and take back the wax crayons. Explain children that they will receive their wax crayon at the end of the event the week after.
3. Take Caltu's puppet in your hand and animate the activity with the puppet.

A Dignified Day

1. Ask participants to raise their hands if they agree that being dignified and ensuring that our children are dignified is really important, that it is valued in the society, and gives them respect from the other members of their community.
2. Ask people to raise their hands if they:
    a. Value their dignity.
    b. Value the dignity of their children.
    c. Believe that children represent their family everywhere they go, at all time, i.e. their faces are the faces of the family.
3. Tell participants that we all know that the way we live each day is important for upholding our dignity and the dignity of our children.
4. Ask the group for examples of important behaviours that they do each day to maintain their *Faces of Dignity*.
5. Turn the flipchart – POSTER – **"DO YOU ENSURE YOUR FAMILY'S DAYS ARE DIGNIFIED?"**
6. Go through the poster.
7. Ask participants if they agree that these activities should be performed daily to maintain their family's *Faces of Dignity*.

8. Remind participants that becoming *Faces of Dignity* will also contribute to enhancing their Community's dignity. Ask participants if they agree that becoming *Faces of Dignity* will enhance their Community's dignity.

Pledge

1. Bring out the **Dignified Day poster**, one per family.
2. Ask each family to hold hands.
3. Ask the parents to repeat the following pledge after you (pause after each sentence). Tell people that they should not repeat any statements that are not relevant to them e.g. if they have no children:

> **I pledge to build a stand for this wash station.**
>
> **I pledge to ensure that there is always soap available at this station so that my family can maintain their *Faces of Dignity.***
>
> **I pledge to help my pre-school age children to wash their faces and hands with soap three times a day: first thing in the morning and before they eat lunch and dinner.**
>
> **I pledge to set a good example for my children by washing my own face and hands with soap three times a day.**
>
> **I pledge to help my family maintain *Faces of Dignity* and enhance my Community's dignity.**

4. Ask any school age children present to repeat the following pledge after "the puppet":

> **I pledge to always wash my face and hands with soap three times a day: first thing in the morning and before lunch and dinner.**
>
> **I will do this so that I always have a *Face of Dignity* and can proudly represent my family.**

5. Clap and congratulate everyone on their pledges and for being valued members of their community.
6. Encourage participants to decorate their wash stations to personalise them.
7. Advise participants to put up the poster inside the house somewhere they will be able to see it each day.
8. Ask whether anyone in the audience has a cell phone. If anyone has one, suggest this person than setting up an alarm three times a day can help them to remind about face washing. Propose your help to set up the alarm on the phone at agreed times. Suggest people with a phone informing their neighbours without a phone each time their alarm rings to remind them about face washing.

ACTIVITY 7: CONCLUSION

What to do

1. Turn the flipchart – CONCLUSION page.
2. Using the puppet, tell participants that we are now at the end of this family forum. Thank them for their participation.
3. Ask participants if they have any question or concerns.

4. Tell participants that you will visit them again in a week to see how they are doing, to answer any questions, and to check on their *Faces of Dignity*.
5. Remind participants that their Community cannot be dignified if all its members are not dignified. Their community's dignity will be publicly recognized and rewarded only if everyone in the community has a *Face of Dignity*.
6. Recommend that all family members attend the second family forum as there will be activities designed for everyone.
7. Check that every family leaves with: **a laminated "Dignified Day" poster and 2 small nails**.
8. Check that families who missed the Community Event receive all their materials (wash station, 2 soaps, soap dish and wash station flyer).
   NB. This will be organised locally by the HVs and/or Berhan enumerators.
9. Play the Dignity Song on a cell phone or any other device while participants are leaving the forum.
10. Put the puppet back into its cover.
11. Wash your hands with water and soap or alcohol-based sanitizer.

End of Family Forum 1.

Report Household Head Names of households who missed the forum on the Family Forum 1 Follow-up Visits Form before ending the event.
These households should be invited to join other Family Fora 1 held with other households in their cluster.
If they cannot join another session, individual follow-up with these families will be organised at their home.
Refer to Appendix B for details of the content to cover during this visit.

Appendices

Appendix A. "Content of Testimonials"

Testimonial 1: **"Building a wash station"** | Interviewee: ideally, a man

Question: Why did you decide to build a wash station stand?

Answer: I built a wash station stand for my wash station so my family and I could wash our faces with soap easily so we have *Faces of Dignity* in the community and.

Question: How did you decide on the location and the height of your station?

Answer: I built my wash station stand near to the entrance of our house so the wash station is protected from the sun and easily moveable at night, when I bring the wash station container, the soap dish and the soap inside. I built the stand like this so my older children can use it easily, but my younger children, who are supported by my wife or myself to wash, cannot play with the soap and make it dirty, or with the tap and waste the water.

Question: Was it hard to build a wash station stand?

Answer: Not at all! *They should explain what they did. E.g.* It was easy and quick to build the stand with some wood I collected. I made a strong structure and then put the container on top of it. I dedicated a baldy to collect the wastewater, and added a dedicated body soap which I put in the soap dish near the container. It was done in less than 2 hours.

Testimonial 2: **"Maintaining a wash station"** | Interviewee(s): a man or a couple

Question: Have you set some roles for taking care of the washing station in your house?

Answer (man): Yes, we have. *They should explain what they have done. E.g.* At first it was not easy to get used to the station, moving the wash station container, filling up the water, always having a dedicated soap. That's why my wife and I decided to set some roles so we don't forget anything. I take care of the materials, bring the station in and out in the morning and at night. My wife is responsible for water and soap and making sure the station is always secured during the day. For instance, that the children do not play with the tap.

Question: Is your wash station always staying outside?

Answer (man): *They should explain what they have done. E.g.* At first, we thought about leaving the washing station outside at all time, but we had nothing good enough to secure it from wild animals or to make sure we do not lose everything overnight. So, our wash station is brought inside our home at night. Because I am often the one waking up and going outside first in the morning, I am the one responsible for putting the wash station container and the soap and soap dish on the washing station stand every morning. At night, after the evening wash, I am also the one responsible for bringing the wash station container and the soap dish inside the house when I close the door. I never forget to put the station outside in the morning or inside at night because the wash station stand is visible from the door of our house.

Question: How do you make sure water is always available at the station?

Answer (woman): *They should explain what they have done. E.g.* I am usually the one responsible to fetch water every morning. Since we have the station, I have dedicated the water of one of our jerry cans to fill the washing station. Like this, that is really easy. I don't have to worry about having enough water for washing 3x a day. Often, there is even some water left inside the washing station container at the end of the day for the day after.

Question: How do you make sure a dedicated body soap is always available at the station?

Answer (woman): *They should explain what they have done. E.g.* Each time I notice that the soap is going to run out soon, I ask my husband for money to buy a dedicated soap for face and body washing. He always gives me money or buys the soap himself. When possible, I buy two soaps at the same time to make sure we always have a spare soap. We thought it would be difficult to keep soap outside because we are not used to do this, but it works and helps us remember to use soap when we wash. That is how we are maintaining our washing station to make sure our family has *Faces of Dignity*.

Testimonial 3: **"Routine use of a wash station"** | Interviewee: ideally, a female primary caregiver

Question: How do you remember to wash your family's faces three times a day?

Answer: In the morning, we used to wash our faces before having the station, so that is easy. But now we have the station, I never forget about using the soap and supporting my younger children so I can wash their eyes and noses thoroughly. I supervise the older ones too. Before lunch, no one has ever forgotten about face washing with soap, as we are also washing our hands before eating at the wash station. We have associated washing hands and washing faces. That is a good reminder. In the evening, we all wash our faces and hands before dinner, our children even remind us about it. In our family, everyone knows about face washing 3x a day with soap, morning, before lunch and before dinner.

Question: What do you like about using the washing station?

Answer: The washing station has made our lives so much easier and we have saved a lot of water. Now we have it, it is so much simpler to maintain our family's *Face of Dignity* by washing our children's faces three times a day with soap. With our wash station and soap always nearby, it is so simple. In my family, we have *Faces of Dignity*, do you?"

Appendix B. "Follow-up with any households that do not attend the Family Forum 1"

HVs should do their best to ensure all households attend Family Forum 1. If any families do not attend, they will need to be followed up at home before Family Forum 2 by the Berhan Activators.
If the household missed the Community Event as well, the wash station, soap dish, soaps (x2) and wash station flyer should be brought to the follow-up visit.
The main activities of the forum should be gone through with the family, with modifications detailed below. The poster giveaway should be provided.

Setting

- Sit with the female primary caregiver and any family member present at home. Make sure the participants are comfortable, i.e. they should be sat in the shade.
- All materials required for the visit are organised and ready to be used.

Materials

**General**	– A facemask, alcohol-based sanitizer, sealable plastic bag for mask disposal		
**Transmission Transmission Routes**	– Trachoma Transmission diagram	**Face wipe Emo-Demo**	– 2 wipes
**Dignified Day Pledge**	– Dignified Day poster<br>– 2 small nails		

Activities

Introduction

1. Wash your hands with soap or alcohol-based sanitiser and ensure you are wearing a facemask before entering the compound. Explain why you are wearing a facemask. If possible, stay outside to do the visit.
2. Greet the female primary caregiver / female adult. If they are not home **do not proceed: wait or return later.**
3. Say that the visit is short (15 mins) and ask her to gather any family members who are close by.
4. Tell the household you have come because they missed the first Family Forum and you would like to share with them what was discussed at the forum. Ask why they didn't attend.
5. Ask if they have any questions before you start your visit.

Trachoma Transmission Routes

1. Ask what they learnt about how trachoma can get from one eye to another eye at the Community Event (should mention flies and hands). Correct them if needed and congratulate them on their answers.
2. Show the **Trachoma Transmission Diagram** and explain that you are going to talk about transmission so that everyone understands why it is so important to get rid of discharge. Say that trachoma is a small germ and is carried in discharge that we can often see on people's faces.
3. Describe the three routes of trachoma transmission, e.g. flies, hands, clothes. Advise the caregiver to wipe her children's discharge with her hand only and to directly wash her hands with soap to remove the discharge from her hands.
4. Ask them to explain what they have learnt about how trachoma can be transmitted and congratulate them on their understanding.
5. Ask what they can do to get rid of the discharge carrying trachoma and maintain their children's dignity / their own dignity. They should answer face and hand washing. If not, probe for what was done in the community event.

6. Say that faces need to be thoroughly washed to get rid of discharge, especially around the eyes and nose. For families with children, add that pre-school children need to be helped so they can wash well.

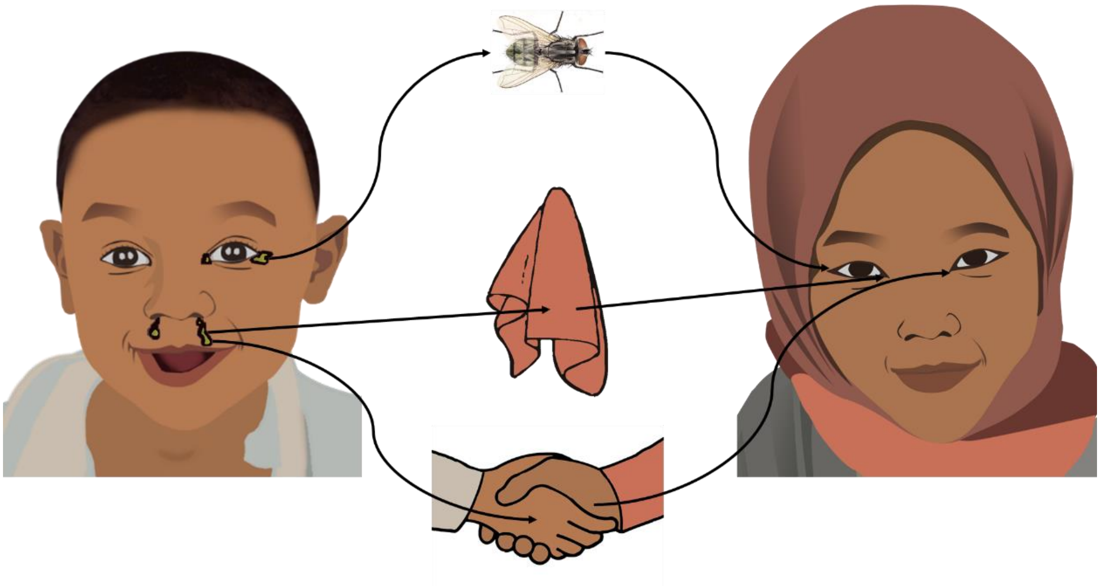

Face Wipe Emo-Demo

NB: The activator should have put foundation on their face before the visit.

1. Ask if they think that they are always able to see all the dirt and discharge on a person's face.
2. Ask if they think that "you" (the activator) have a clean face.
3. Explain that you are going to use a wipe to confirm what they have just said. Explain that the wipe is just a cloth that is a bit wet.
4. The activator should wipe one side of his/her face and show the dirty wipe to the family. Is this a surprise?
5. Say that faces can look clean even when they are not. That is the case for all of us, but especially for children who are spending a lot of time outside, playing with friends, etc.
6. Ask what they could do to make sure their faces/ children's faces are never dirty and undignified when they appear clean (i.e. wash face with water and soap). **Let people answer*.* Probe the family for what the family did in the community event.
7. Remind the household that face washing with soap is important for true cleanliness and dignity.
8. Ask if they agree that soap is the only way to achieve *Faces of Dignity*, that they value their dignity and the dignity of their children, and that they want to wash faces with soap regularly so their whole family have *Faces of Dignity.*

Wash station

1. Tell the household that to keep their *Faces of Dignity*, face washing with water and soap is key, but that it is not always easy to gather all the materials and the soap to wash.
2. Ask the female primary caregiver to bring forward the wash station, the soap dish with the soap and the wash station flyer they received after the community event. Ask if they have already started using their wash station (or used one before, e.g. in town) and what they like about it.
3. Say that you would like to quickly discuss how to construct a stand for their wash station and how the station should be used.
4. Use the **wash station flyer** to describe how to build and use the station. Focus on a male household head if he is present:

a. **Location and Structure:** a 'good' location is outside, close to the home and in the shade. The wash station and soap dish are on a built, sturdy wooden structure that is secured and cannot be knocked over by children or animals.
b. **Height:** if the household has children, very young children cannot reach the tap and soap without help, but the station should be accessible to all other family members.
c. **Drainage:** a bowl or stones/sand are placed under the tap to prevent the ground from becoming muddy.
d. **Water:** someone is responsible for filling the wash station container with water so there is always water available for use. The wash station container should not be taken to the water, the tap is fragile. The tap should be closed whilst lathering the hands or rubbing soap on faces.
e. **Soap:** a dedicated soap is kept in a soap dish at the wash station. The soap is always clean and available for body washing when it is needed.
f. **Night:** at night, if there is no fence around the station, it might be preferable to take the wash station container and the soap and the soap dish inside the house. Someone should be responsible for bringing the station, soap dish and soap outside every morning.

5. Ask whether they have any question or concerns about how to set up their wash station.

Dignified Day Pledge

1. Ask the household if they value their dignity, the dignity of their children (if they have any), and if they believe that children represent their family everywhere they go, at all time, i.e. their faces are the faces of the family.
2. Ask the household if they also value their community and would like to contribute to their community's dignity.
3. Say that we all know that the way we live each day is important for upholding our dignity and the dignity of our children.
4. Show the poster "Do you ensure your family's Days are Dignified?" and go through the poster.
5. Ask the household if they agree that these activities should be performed daily to maintain their family's *Faces of Dignity* and enhance their community's dignity
6. Ask the family members to repeat the following pledge after you (pause after each sentence). Do not read out statements referring to children if there are no children in the household:
    a. I promise to build a stand for this wash station and make sure there is always water and soap there so that my family can maintain their *Faces of Dignity* and we can contribute to enhancing our community's dignity.
    b. I pledge to help my pre-school age children to wash their faces and hands with soap three times a day: first thing in the morning and before they eat lunch and dinner.
7. Congratulate the household and encourage them to decorate their wash station to personalise it.
8. Advise them to put up the poster inside the house somewhere they will be able to see it each day.

Conclusion and Giveaways

1. Tell the household that this is the end of the visit. Ask if they have any question or concerns. Thank them for their time.
2. Tell the household that the second Family Forum will take place in a week and that you recommend that all family members should do their best to attend as there will be activities for everyone. Discuss if they think it will be difficult to attend.
3. Check that the household has received: **a laminated "Dignified Day" poster, and 2 small nails.**

4. Wash your hands with water and soap or alcohol-based sanitize after leaving the compound. Ensure you are safely disposing your facemask in a sealable plastic bag at the end of the morning visits or at the end of the day.
