# Supplementary material for: Cluster randomised controlled trial of double-dose azithromycin mass drug administration, facial cleanliness and fly control measures for trachoma control in Oromia, Ethiopia: the stronger SAFE trial protocol
Source: BMJ Open. 2024 Dec 23;14(12):e084478. doi: 10.1136/bmjopen-2024-084478 (PMC11751794; doi:10.1136/bmjopen-2024-084478)
Supplement: online supplemental file 6 [file bmjopen-14-12-s006.pdf]

EVENT 5 – HOUSE CALL 2
ACTIVATOR MANUAL

**Purpose**	To provide ongoing support to motivate families to wash faces with soap 3x a day throughout the year. Provide support to help families maintain their wash stations.
**Responsible parties**	1 trained Activator
**Participants**	All members of a household present at the time of this unannounced visit. Households living within the 'yolk' of an intervention cluster who received House Call 1.
**Location**	Each participant's home
**Duration**	30 to 45 mins
**Timing**	Workdays (except Friday morning), 9am to 5pm

Setting

- Sit in a comfortable place chosen by the household.
- Ask permission to move around their home with them during the visit.
- Seek shade or move inside when using the tablet so that everyone can see the screen clearly.

Materials

General

- COVID-19 prevention materials: 1 facemask, alcohol-based sanitizer, sealable plastic bag to dispose the mask
- House Call 2 – Checklist
- Caltu's puppet
- A pen
- A tablet with full battery – *NB. To amend if not feasible.*
- Notebook
- A cell phone or any other device to play the Dignity Song

Video-Demo

- Trachoma Transmission Routes diagram

Certification

- Certification sticker

Activities

ACTIVITY 1: INTRODUCTION

Purpose

- To provide introduction to the household visit.
- To address any concerns or questions arising from the first House Call or the Campaign.

What to do

1. Wash your hands with soap or alcohol-based sanitiser and ensure you are wearing a facemask before entering the compound. Explain why you are wearing a facemask. If possible, stay outside to do the visit.
2. Greet the female (or male) primary caregiver and thank them for welcoming you into their home.
   NB: if the female primary caregiver is not home **do not proceed with the visit until they can be found**. Either wait for them to return home, or return later that day or the following day.
3. Conduct the visit with the Household Head and any other household members if the household does not have a female primary caregiver.
4. Say that the visit will take no more than 45 mins.
5. Ask for any family members not present who are close by to be sent for.
6. Say that you are visiting to see how they are getting on with using their wash station.

ACTIVITY 2: VIDEO DEMO

Purpose

- Can consistently wash young children's hands and face with soap using an effective and efficient technique.
- Self-efficacy relating to effective and efficient washing technique for self and pre-school children.
- Perceive soap as important to use each time faces are washed.
- Accept responsibility for hygiene of young children.

What to do

1. Ask a child (aged between 1 and 6yo) to wash their face as they normally would. If no young children are in the home, select an older child or another household member. Do not probe or give any recommendations.
2. Video the person washing from the time when you ask them to wash until the time they finish washing and play the Dignity Song during the demonstration.
   NB. This will be amended after pilot-testing in the field.
3. **Complete the checklist.**
4. Play the video back to the household.
5. Congratulate the volunteer / household on the parts of the wash they did "correctly" and say that it is clear they are working hard to maintain their *Faces of Dignity*
6. Discuss:
   a. **Usual practice:** Is this how the child/person usually washes? Why / why not?
   b. **Wash station use:** Who uses the wash station and when? Discuss any barriers to use and attempt to find solutions.
   c. **Soap use**: Soap should be used for all face washes, especially small children who represent the family in the community and need help to maintain their *Faces of Dignity*. Discuss any barriers raised and attempt to find solutions.
   d. **Support (if the person washing was a pre-school child):** Ask whether the family believes young children can wash their own faces thoroughly enough to remove discharge and remind them that they need help every time they wash.

e. **Thorough washing of the eyes and nose:** We need to rub around the eyes and nose every time we wash our faces. Encourage children to close eyes tightly.
f. **Closing the tap to avoid wasting water**: During every wash, the tap should be closed whilst the person washing (or being washed) is scrubbing his/her face with soap.
g. **Hand washing:** Show the Trachoma Transmission Routes diagram and remind family that our hands are naturally washed when we wash our faces, but any children we wash need their hands washed too. Both hands and faces should be clean to prevent eye and other infections. Remind caregiver that wiping should be done with the hand and hands should be washed with soap immediately to remove discharge.

7. Remind the household that everybody needs to wash faces with soap x3 a day to maintain their dignity and set a good example, but that young children are especially important and should be supported until they go to school.

Activity 3: Wash station review

Purpose

- Can maintain the functioning of a constructed wash station.
- Functioning wash station is consistently available and accessible.
- Self-efficacy relating to maintenance of wash station.

What to do

1. Ask to view the wash station and **complete the checklist**.
   If the household **does not have a wash station,** ask what they have done with it and why. Discuss how they can make sure soap and water are always readily available for face and hand washing.
   If the household **has a wash station** or **has a wash station without a wash station stand** discuss:
   a. **Water availability:** Are they able to ensure water is always available at the wash station? Discuss potential solutions e.g. filling the wash station immediately after water is collected / at a particular time of day. Dedicating someone to fill the station.
   b. **Soap availability:** Are they able to ensure soap is always kept at the wash station? Discuss potential solutions e.g. use of soapy water, asking household head for money to replace soap when it starts to run low.
   c. **Location:** Encourage the family to protect the wash station from the sun to avoid sun damage.

Activity 4: Wash Station Maintenance

Purpose

- Can repair a dysfunctional wash station.
- Self-efficacy relating to repair of wash station when it becomes dysfunctional.
- Functioning wash station is consistently available and accessible.

What to do

1. Play the Wash Station Maintenance video on the tablet.
2. Repeat the video if the household requests it, or if the household is large and cannot all view the screen. Aim to get the attention of the Household Head as a priority.
3. Discuss the video and answer any questions about replacing broken or leaky taps.

NB. This activity will be piloted and amended according to feasibility. If this can't be set up, key elements regarding wash station maintenance should be communicated to Activators during the training, so they can facilitate a discussion with the households instead of showing a video.

Key elements include: habits and behaviours to adopt to avoid damage to the wash station container (esp. making sure the tap does not break by not moving the station around, go collect water with it, etc.), what to do if the tap leaks (e.g. use glu around the tap, where to get it, how much it costs), what to do if the tap breaks (e.g. where to get a new tap, price, how to fix the tap, etc.)

ACTIVITY 5: POSTER AND DANGLER REVIEW

Purpose

- Perceive face washing to be important for maintaining dignity of self and family.
- Perceive effectively washing face at least three times per day as important, all year around.
- Accept responsibility for hygiene of young children.

What to do

1. Ask participants to see their "Dignified Day" Poster and **complete the checklist.**
2. Ask participants to see their Dangler and **complete the checklist.**
3. If poster and dangler are present, congratulate the HH on having their poster and dangler. Remind that these are cues to remind them about face washing 3 times a day and that, if they have phones, setting an alarm 3 times a day can also help them.

ACTIVITY 6: WASH STATION CERTIFICATION

Purpose

- Engaging, accessible, appropriate, strategically placed washing prompts/cues are visible.
- Perceive effectively washing face at least 3x a day as important, all year around
- Perceive face washing to be important for maintaining dignity of self and family.
- Perceive soap as important to use each time faces are washed
- Perceive an expectation from neighbours & husbands to maintain clean faces of self & family
- Create the impression that everyone in the community washes faces with soap x3 a day

What to do

1. Take Caltu's puppet in your hand and animate the activity with the puppet (only if some children are present).
2. Congratulate the family again on working hard to maintain their *Faces of Dignity* and on enhancing their Community's dignity.
3. Show the video of the Influential Role Model congratulating the family on their *Faces of Dignity*, thanking them for being dignified community members and explaining that the a certification sticker acknowledges their efforts at washing faces with soap x3 a day and should be placed on the wash station. **Refer to Appendix A** for content.
   NB. This is likely to be an audio-recorded message from the Influential Role Model. To be amended after piloting.
4. If the family has a wash station and a wash station stand:
   a. **Give** the Household Head (or nearest representative) **the Certification sticker** and ask them to place it on the wash station.
5. If the family has a wash station but has still not built a wash station stand:
   a. Provide personalised suggestions to troubleshoot the issues with building a stand and remind participants of the benefits of using one (e.g. easiness of use, etc.)
   b. **Give** the Household Head (or nearest representative) **the Certification sticker** and ask them to place it on the wash station.
6. If the family does not have a wash station any longer:
   a. Provide advice on dedicating other materials (e.g. a specific jug or bowl) for face and hands washing.

   b. **<u>Give</u>** the Household Head (or nearest representative) **<u>the Certification sticker</u>** and ask them to place it on the chosen dedicated material.

7. **Complete the attached checklist.**

ACTIVITY 7: CONCLUSION

What to do

1. Lead this activity using the puppet (only if some children are present).
2. Say that we are now at the end of this HH visit.
3. Ask if there are any final questions about any aspect of this visit or the *Faces of Dignity* campaign.
4. Say that they will be visited again [tell them the likely month/season of this visit].
5. Congratulate the household on their hard work to create new habits to maintain *Faces of Dignity* for their whole family, especially the youngest children. Encourage them to keep washing faces x3 a day so they represent themselves well in the community.
6. Say that a little gathering will take place at *[location where the Dignity Banner is up in the community]* after all households of their community have been visited. If everyone has become a *Face of Dignity*, their community will be publicly recognized as a Dignified Community and rewarded in presence of all the community leaders. HVs will inform them about the gathering and they are welcome to join the celebration.
7. Thank the family for their time.
8. Put the puppet back into its cover.
9. Wash your hands with water and soap or alcohol-based sanitize after leaving the compound. Ensure you are safely disposing your facemask in a sealable plastic bag at the end of the morning visits or at the end of the day.

End of House Call 2.

NB. At the end of House Call 2 delivery, community leaders will be gathered at the location where the Dignity Banner is up to publicly declare the community a Dignified Community. The Dignity Banner will be amended at the occasion. Consecutively, community leaders will be rewarded for their contribution to the intervention. This section will be amended after discussing options with the field team and Berhan.

Appendices

Appendix A. "Content of Influential Role Model Testimonials – Wash station certification"

Testimonial 4: **"Congratulating participants"** | Interviewee: Lead Influential Role Model

"Congratulations for working so hard to become a dignified family and all having *Faces of Dignity*. I personally want to thank you all for being dignified community members. Your community is proud of you as everyone needs to work together to ensure our community all have *Faces of Dignity*. Thanks to all of you, our community is a Dignified Community! The certification sticker which is given to you now acknowledges your efforts over the last month to wash your faces x3 with soap a day to become *Faces of Dignity*. Like me, stick it on your wash station container to always remember your progress. Congratulations again and keep up the good work!"
