# Supplementary material for: Cluster randomised controlled trial of double-dose azithromycin mass drug administration, facial cleanliness and fly control measures for trachoma control in Oromia, Ethiopia: the stronger SAFE trial protocol
Source: BMJ Open. 2024 Dec 23;14(12):e084478. doi: 10.1136/bmjopen-2024-084478 (PMC11751794; doi:10.1136/bmjopen-2024-084478)
Supplement: online supplemental file 7 [file bmjopen-14-12-s007.pdf]

EVENT 5: HOUSE CALL 2 – DATA FORM

Household ID: ☐☐☐☐☐☐ Date: ☐☐/☐☐/☐☐ Time: ☐☐:☐☐
Ethiopian calendar *Ethiopian time*

Household Head Name: ____________________________ Activator Name: _________________________

VIDEO DEMO

Age: ◯◯ years (enter 01 for age=1, 02 for age=2, etc.) Gender: ◯ Male ◯ Female

Put an "X" in the box if the person: *(tick all that apply)*

- ◯ Uses the wash station with tap
- ◯ Uses soap. If yes, which soap was used: ◯ Laundry soap ◯ Body soap ◯ Soapy water
- ◯ Is supported by someone (e.g. mother, father, sibling)
- ◯ Washes clearly around the eyes and nose
- ◯ Closes the tap whilst scrubbing his/her face
- ◯ Has hands washed (if face wash is performed by someone other than the child)

WASH STATION REVIEW

Water container with tap is present:

◯ Yes, inside ◯ Yes, outside ◯ Cannot be seen → Ask where the water container is
Record where it is: ____________________________

Family has built a station for their wash station water container:

- ◯ Yes, suitable height.
- ◯ Yes, too high → Discuss changing the height so it can be used by more family members.
- ◯ No → Discuss how they are using the material and how the station could help them.

There is water in the wash station container: ◯ No ◯ Yes, but no water flows when the tap is open ◯ Yes, 1/4 full ◯ Yes, half full or more

The wash station container is functional: ◯ Yes ◯ No, why: ___________________________________

There is bar soap at the station: ◯ Yes, in soap dish ◯ Yes, not in soap dish ◯ No

There is a soapy water bottle at the station: ◯ Yes, attached to the station ◯ Yes, not attached ◯ No

POSTER AND DANGLER REVIEW

Location of the poster:
- ◯ On wall, suitable height
- ◯ On wall, too high → Suggest changing location and explain why
- ◯ Present, but not visible → Suggest nailing to wall
- ◯ Not seen in home ◯ Other: ______________________________

Location of the dangler:
- ◯ Hung. If yes, where: ◯ In the kitchen ◯ Place people take meals
- ◯ Present in the house, but not visible → Suggest hanging the dangler
- ◯ Not seen in home ◯ Other: ______________________________

WASH STATION CERTIFICATION

Certification sticker has been given to household: ◯ Yes ◯ No
Certification sticker has been stuck on the wash station container or a dedicated material: ◯ Yes ◯ No

Please keep this checklist safe and give it to responsible person at Berhan when you see them.
