# Supplementary material for: Cluster randomised controlled trial of double-dose azithromycin mass drug administration, facial cleanliness and fly control measures for trachoma control in Oromia, Ethiopia: the stronger SAFE trial protocol
Source: BMJ Open. 2024 Dec 23;14(12):e084478. doi: 10.1136/bmjopen-2024-084478 (PMC11751794; doi:10.1136/bmjopen-2024-084478)
Supplement: online supplemental file 8 [file bmjopen-14-12-s008.pdf]

Supplementary 2 Stronger SAFE Cluster Allocation Map

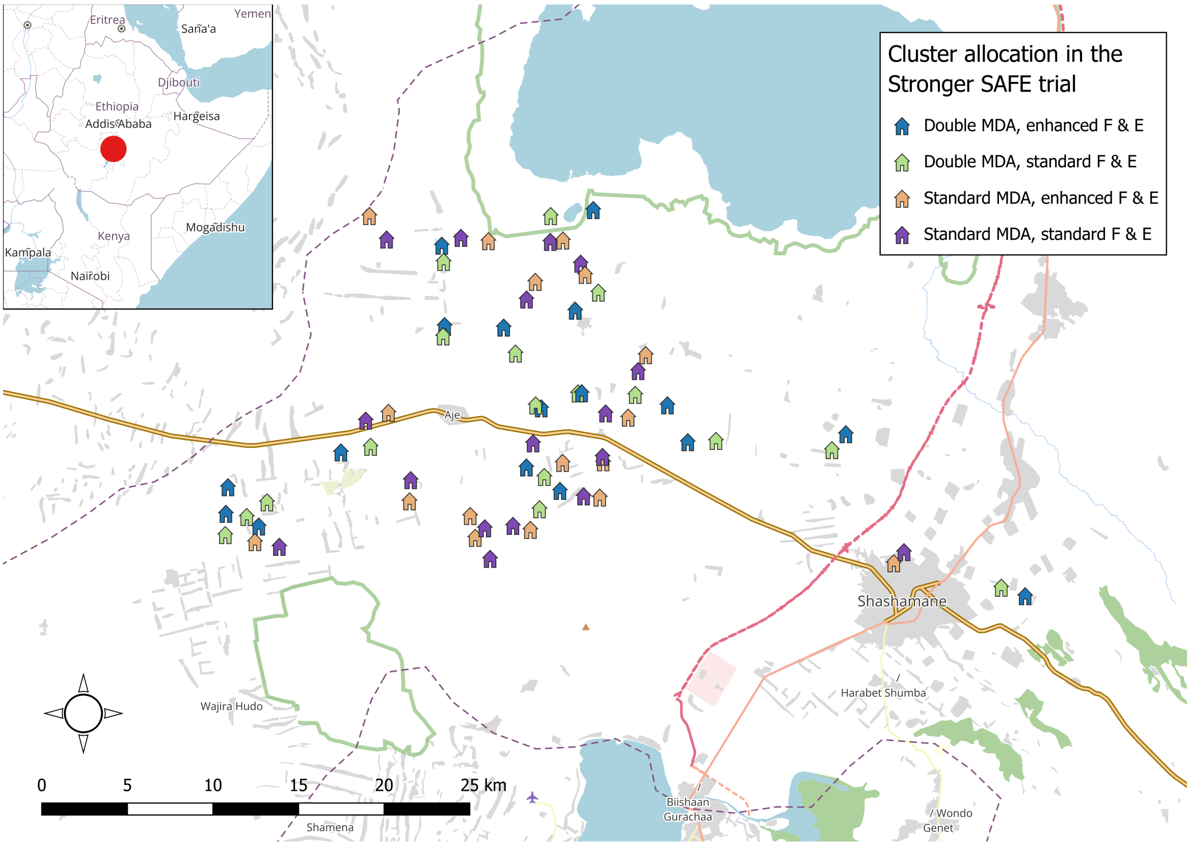

Map showing cluster allocation by arm. The red circle denotes the study area in West Arsi, Oromia. A house icon indicates a single cluster, coloured by arm. Each cluster is comprised of 90 households (approximately three administrative garee units). The 'yolk' includes 50 households and the 'white' the remaining 40 households. The 'white' provides a buffer zone for the household-level enhanced F&E interventions. Azithromycin MDA is distributed to the kebele zone, and therefore clusters are matched pairs according to the randomisation procedure, with an ample buffer zone between clusters receiving single and double MDA. Standard MDA indicates single dose azithromycin. Double MDA indicates two doses of azithromycin. Standard F&E refers to programmatic F&E measures. Enhanced F&E includes the Stronger SAFE hygiene behaviour change and fly control intervention packages.
