# Supplementary material for: Cluster randomised controlled trial of double-dose azithromycin mass drug administration, facial cleanliness and fly control measures for trachoma control in Oromia, Ethiopia: the stronger SAFE trial protocol
Source: BMJ Open. 2024 Dec 23;14(12):e084478. doi: 10.1136/bmjopen-2024-084478 (PMC11751794; doi:10.1136/bmjopen-2024-084478)
Supplement: online supplemental file 9 [file bmjopen-14-12-s009.pdf]

Supplementary 3

Sample Size for Entomological Observations

Fly-eye contact is the most important entomological outcome measure because it is the most relevant to transmission of Ct. We have previously demonstrated that the mean fly-eye contact rate ranged from 30-50 contacts in ten minutes of filmed fly-eye contact in children, with a standard deviation of 40.[18] Six households per cluster will be sampled, as recommended by WHO[24], based on an assumption of a cluster mean of 40 fly-eye contacts (in ten minutes) and a range of cluster means between 10-70, yielding an approximate estimate of 0.375 for the ICC. Therefore, six households per cluster (across 27 clusters) gives 80% power to detect a reduction of 50% in the mean number of contacts. To include this number of clusters, we will treat both enhanced F&E clusters as a single arm to be compared with the two (combined) standard F&E arms.

Durability of fly control interventions are assessed in line with WHO guidance for Phase 3 vector control trials.[24]

To determine durability of PTH, physical integrity, biological efficacy and permethrin content of PTH are assessed every six to eight weeks. Twenty-seven clusters are randomly selected from the 34 Enhanced F&E intervention clusters. From each selected cluster, we randomly select one household, from which one child aged 3-9 years is randomly selected, and their PTH (hat or scarf) is evaluated. Biological efficacy is determined using WHO cone testing in our insectary in Shashemane, Oromia. Permethrin content is determined using high performance liquid chromatography (HPLC) from a square section of PTH (either hat or scarf), performed at LSHTM in the UK.[21] Measuring the permethrin content in 27 items of PTH allows an accuracy of +/- 4 mg, assuming that the SD of permethrin content between scarves is 10.[21]

From the 27 selected clusters, a qualitative survey designed to understand adherence and acceptability of entomology (E) interventions is conducted in 15 randomly selected durability households every six to eight weeks. The durability of OBT is also measured during this survey alongside data collected during entomology reinforcement visits by health volunteers, to estimate the physical integrity, trap functionality, quality assurance of trap components, trap attrition and household retention. This provides an estimate for the proportion of traps that have been lost with a margin of error of no more than ± 7%.

Sample Size for Hygiene (Face washing) Observations

Three key behavioural outcomes will be directly observed during a three-hour structured observation session: the proportion of individuals who washed their face; the proportion of individuals who washed their face with soap; and the proportion of observed face washes that included soap. The sample size calculation is based on the proportion of face washes that include soap during a three-hour observation period, as this outcome required the largest sample size. Assuming a prevalence of 25% in the control arm and a design effect of 1.33, a sample of 204 individuals per arm will provide 90% power to detect an increase in the prevalence to 40%. For practical reasons we opted to sample all individuals in three households in the 34 clusters in each arm (n=204 households). As with the entomological outcomes, for this analysis all the enhanced F&E clusters will be treated as a single trial arm, and the standard F&E arms combined to act as the control arm.

Secondary Outcomes

Ocular Outcomes

- The prevalence of ocular *Ct* in children aged 1-9 years determined by *Ct*-specific PCR estimated from samples taken at 12 and 24 months, by trial arm.
- Prevalence of TF and TI in children (1-9 years) at 12, 24 and 36 months, by trial arm.

WASH Outcomes

- Proportion of children (1-9 years) with clean faces determined by visual inspection and moist cotton wipe (quantitative personal hygiene assessment tool (qPHAT))[33], at three and 36 months post-delivery of the F intervention, by trial arm.
- Face washing behavioural outcomes: Mean daily frequency of good quality face washing among pre-school children, school-age children and primary caregivers, proxies of behaviour (presence of soap and water at wash stations) and behavioural determinants assessed at three and 36 months post-delivery of the F intervention, by trial arm.

Entomology Outcomes

- Fly-control indicator: Density and species composition of flies captured by arm determined every six to eight weeks by odour-baited trapping for 24 hours in 324 sentinel households.
- Fly-control indicator: Risk of fly-eye, fly-nose and fly-mouth contact from eye-seeking flies including *M. sorbens* in children aged 2-9 years, by arm (1+3 [standard F&E] vs 2+4 [enhanced F&E]), determined by fly-face observation and videography every six to eight weeks in 324 sentinel households.
- Fly-control indicator: Estimated physical and chemical durability of the insect control products/interventions, determined every six to eight weeks in 27 households (one newly selected household per sentinel enhanced F&E cluster)

Process Indicators

- Process indicators to measure estimated coverage (exposure, adherence, recall) of the interventions (antibiotic and F&E).

General Health

- Prevalence of malnutrition by trial arm (height for age and height for weight Z scores). Measurement of height, weight, mid-upper arm circumference (MUAC), knee-heel length and head circumference in children (1-60 months) will be done at 36 months.
- Prevalence of clinic and hospital visits (specific and all-cause) for children (1-60 months) at 36 months.
- Prevalence of diarrhoeal and respiratory illness in children (1-60 months) at 36 months.

Clinical Follow-up Assessments

After the first baseline assessment, 6 follow-up clinical outcome assessments will be conducted in the same clusters at 2 month, 12 months, 14 months, 24 months, 26 months, and 36 months.

The census will be updated and new residents in the cluster will be invited to participate, after agreeing to the informed consent process.

The follow-up assessment will be conducted on a random selection of at least 60 children in each cluster aged 1-9 years. Since it is a random selection, children assessed in the previous round may or may not be assessed again.

Each follow-up assessment will include the following components:

1. Ocular Clinical Examination
The randomly selected children will be examined for signs of trachoma. Briefly, the trachoma grader (nurse) will examine the upper tarsal conjunctiva of each eye with a 2.5x magnifying loupe with a torch or in adequate sunlight and will grade the conjunctiva using the detailed WHO trachoma grading system.[27,28]

2. Photography
Digital photographs will be taken of each eyelid examined, for independent detailed grading. During or immediately after ocular examination is completed a Nikon D500 camera with macro lens will be used to take conjunctival photographs. Digital photographs will be taken of the faces and hand for independent grading. These photographs will be used to assess correlations between visual cleanliness, fly-eye contact rates and active trachoma. It will also be possible to use these photographs to investigate inter-rater agreement about the cleanliness of faces and hands.

3. Conjunctival Swab Sample Collection
One swab sample will be collected from the left upper tarsal conjunctiva by the nurse. Initially a drop of preservative free topical anaesthetic, Proxymetacaine Eye Drops (Minims, Bausch & Lomb, U.K.) into the conjunctival inferior fornix, so that the child does not experience any discomfort. The upper eyelid will then be everted. The conjunctival swab sample will be collected from the upper tarsal conjunctival surface (posterior surface of the upper eyelid) by passing the swab across the surface.

The sample will be secured directly in a dry tube and the tube placed immediately in a cool box in the field and then transferred to a -20^{0}C freezer for storage later the same day in Shashemane. The swabs will be transferred in regular batches to the regional Health Bureau Laboratory, Adama where they will be stored in a -80^{0}C freezer. In Adama, the DNA from these swabs will be extracted and tested for *Chlamydia trachomatis* by PCR.

Air control swabs will be collected, by holding a swab in the air for 20 seconds, in the same location as clinical samples were collected. One control swab will be collected at random for each 50 sample swabs. The swab will be handled, stored and processed in the same manner as the ocular swabs.

4. Anthropometry
Trained nurses will record tympanic temperature, height, weight, mid-upper arm circumference (MUAC), knee-heel length and head circumference of *all* 1-60 month-old children and of all children enrolled into the trial at baseline. Measurement will be taken in triplicate, with the median measurement used for analyses. Training using this methodology in Ethiopia demonstrated reproducibility of anthropometric measurements among trained anthropometrists from rural communities. Measured at 36 months.

- Height will be assessed with a ShorrBoard (ShorrBoard®, Shorr Productions, Olney, MD, USA)
- Weight with an electronic scale (Seca 874/878 flat floor scale, Seca, Hamburg, Germany)
- MUAC will be measured to the nearest millimetre using non-stretch MUAC tapes.
- Knee-heel length will be measured using a knee height calliper (Shorr Productions, Olney, MD, USA)
- Head circumference will be measured with a ShorrTape (Shorr Productions, Olney, MD, USA)

Entomological Outcomes Measurement

Sentinel households

Vector population parameters are highly variable, seasonal, and fluctuate according to local conditions. For this reason, entomological outcomes must be measured in a representative sample of locations, and repeated measures taken over time. This allows estimation of how individuals and communities are exposed to vectors and describes the seasonality of this exposure. To measure the efficacy of a vector control intervention that impacts vector population dynamics, differences in vector population age structure, sex ratio, parity and density should be measured in both intervention and control arms.[24]

Entomological data will be collected every six to eight weeks over the 36-month trial in a standardized manner across all four study arms using a sample of six randomly selected households per cluster in 54 clusters (324 households in total). These include households selected from 27 clusters receiving 'Standard F&E' and 27 clusters receiving 'Enhanced F&E', including the fly-control measures. Both clusters and households within clusters will be selected randomly according to WHO protocol, although households for selection will be restricted to include those with at least one child aged between two and nine years old.[24] In addition to the entomological outcome measures specified below, environmental conditions (ambient temperature and humidity, rainfall presence/absence/history, wind speed) will be measured at each sentinel household visit.[24] In line with WHO guidelines for Phase III trials, the physical and chemical durability of the intervention products will also be monitored.[24]

Fly-face contact

Fly-face contact will be observed, and recorded by videography, for up to two children per household between the ages of two and nine years, between 10:00 and 15:00. Specifically, the participant will sit comfortably outside their house, if the house is in an 'enhanced F&E' cluster (i.e. in arms 2 or 4) the participant will wear their PTS around their neck and in the manner in which they usually use it. If the house is in a 'standard F&E' cluster the participant will wear a placebo PTS supplied by the investigators. The participant's face will be videoed for ten minutes by the entomological field worker. During this time period, the number (frequency) of fly contacts will be manually recorded and scored.

Fly population density and diversity

At sentinel households in the enhanced F&E clusters, the odour-baited traps (OBT) already deployed for population suppression will be additionally used for monitoring purposes, in addition sticky paper traps will be used to capture flies. For 48 hours, the 5-litre water bottle that serves to collect and kill flies will be replaced; in the standard F&E arm sentinel households, OBT will be deployed for the 48-hour monitoring period only. After collection, trap catch will be counted, calyptrate Dipterans identified to family level using a dissecting microscope and taxonomic keys, further, *M. sorbens* to species-complex level. Female *M. sorbens* will be dissected to determine gravidity and parity. Example specimens will be pinned. Trap catch of other non-target, but beneficial, insects (e.g. bees) will be recorded.

Durability of 'Push': Permethrin treated scarves (PTS)

The durability of the 'push' intervention product, PTS, will be tested every six to eight weeks in 27 randomly selected households (one per enhanced F&E cluster). Selection is semi-randomised, ensuring that a child aged 3-9 (who will have received a PTS) lives there and durability has not previously been tested there. If households do not meet these criteria, or if there are no children in the household who still have the PTS, another household will be randomly selected. By sampling from new households each time, the sampled PTS will reflect the age of the PTS in the trial.

Durability of the PTS will be tested according to four parameters: attrition (scarf loss), biological efficacy (fly-face/-eye contact inhibition in the field and laboratory bioassays), chemical content (amount of active ingredient) and physical integrity (physical condition of the scarf). When loss of protection from fly-eye content is observed in the biological efficacy trials, alongside a decrease in permethrin content, or after one year (whichever occurs earliest) replacement PTS will be distributed across all enhanced F&E study arms. The acceptability of the PTS to participants and communities will be assessed as part of the 'participant exposure interviews'

Attrition

A questionnaire will be conducted with the parents or guardians of the children who received PTS, asking how many PTS the household received, if any were lost or given away and what the reasons were for losing or giving them away. Actual attrition will be calculated according to the known number distributed to those households.

Biological Efficacy

Fly-face/-eye contact will be measured for each child in the household who received a PTS. Fly-face/-eye contact will be observed, and recorded by videography, as follows: the participant will sit comfortably outside their house wearing their PTS, and their face will be videoed for five minutes by the entomological field worker. During this time period, the number (frequency) of fly contacts will be manually recorded and scored. The same scarf will then be removed for permethrin content assays (below, 'chemical content') and further laboratory bioassays, and a new, replacement PTS will be immediately distributed to that household. Laboratory bioassays will incorporate either arm-in-cage bioassays that determine the protective efficacy of the fabric against contact from colonised *M. sorbens* and relative to an unused product, or a modified WHO cone test or both test types.

Chemical Content

Samples of PTS will be individually packed in foil, labelled and stored at 4 °C for analysis of permethrin content by high pressure liquid chromatography (HPLC).

Physical Integrity

Physical integrity will be measured by scoring for wear and tear according to a standardised scoring system.

Durability of 'Pull': odour-baited traps (OBT)

The physical integrity, trap attrition and household retention will be determined using a standard sampling questionnaire and direct observation. Trap attrition will be defined as recording if traps were moved, lost, modified, or not maintained the traps as per study protocol. Household retention will record those who did not accept to participate, withdrawal and coverage rates. Further questions will explore adverse effects and events due to use of OBTs and general acceptance by participants.

Trap functionality will be assessed by measuring the trap catch, and quality assurance of trap components conducted by scoring each component for wear and tear according to a standardised scoring system. Questions that explore whether participating households have correctly deployed the lure (i.e. replacing the lure after four weeks) will be included in the standard sampling questionnaire, for further quality assurance the volume of lure remaining in the trap, and its physical condition, will be recorded.

WASH Outcome Measurement

WASH Behaviour change outcomes will be primarily assessed through direct observation, and complemented by script-based pictorial recall and the use of proxy indicators of face washing practice. The main outcome will be observed face-washing behaviour. Data will be collected at baseline, three and 36 months post-delivery of the F intervention to give an indication of both initial behaviour change and the sustainability of behaviour change. We will also assess facial cleanliness qualitatively and quantitatively at each of these time points. Repeatedly visiting the same households for observations is associated with considerable reactivity in the control arm, as well as over-reporting in the intervention arm. Therefore, at each visit, we will collect this data in three randomly selected households in each of the 17 clusters per study arm (204 households in total), with both trial standard F&E arms grouped as one (standard F&E) and both enhanced trial F&E arms grouped as one (enhanced F&E) for analysis. Clusters and households within clusters will be selected randomly at each time point, although households for selection will be restricted to include those with at least one child aged between one and six years old due to our interest in observing outcomes amongst pre-school age children and will not overlap with the sentinel households selected for the entomological studies to avoid bias.

Data collection methods to be applied in the 204 randomly selected households will include:

- Direct, structured observation of actual hand washing and face washing practices (including soap use) in selected households. Observation will take place for around three hours, at different points in the day when hand and face washing is promoted. The precise timing of observation will be piloted ahead of the trial to ascertain when the most face washing events are recorded.
- Household survey to collect data on WASH infrastructure and facilities, self-reported behaviour and face washing determinants and an adapted script-based diary interview to collect self-reported data on face washing practices.
- Observation of proxy indicators of face washing including presence of soap and water at the face washing location and demonstration of face washing practices.
- Assessment of facial cleanliness in all pre-school and school-age children plus primary caregivers. This will be measured qualitatively by observed presence of ocular and nasal discharge and flies around the eyes and quantitatively using the quantitative personal hygiene assessment tool (qPHAT). The ‘dirtiness’ of the child’s face is assessed using a cotton pad, moistened with water, which is wiped gently on the skin of the child’s face in a standardized manner. The colour of the darkest point of the pad after wiping is then compared to a standardised colour scale.[33]

We will pilot test promising outcome measures during baseline data collection to validate behavioural outcome measures and assess levels of agreement between different measures. Outcome measures tested will be; directly observed washing practice during early mornings and in the middle of the day, observation of proxy indicators (such as presence and convenience of soap and water, facial cleanliness), face washing demonstrations, and self-report through questionnaire, and structured script-based recall interviews of daily activities with soap and water use noted. We will pilot test these methods for feasibility, acceptability and reliability and will assess criterion validity of self-report and proxy measures against directly observed practice. In intervention households we will also assess the reactivity of these indicators to interventions.

Process evaluation of F&E

A mixed-methods process evaluation will be conducted to explore how intervention delivery and participants’ responses to the intervention affect uptake of the face washing and fly control interventions in the context in which the intervention is implemented. We will assess four aspects of intervention implementation (the fidelity, delivered dose, received dose and reach of the intervention) as well as recall, comprehension, acceptability and psychosocial responses among participants and implementers. We will examine factors associated with face washing and behaviour change (‘F’ intervention) and PTS use (‘E’ intervention). Contextual factors influencing intervention delivery and receipt will also be recorded.

Data collection methods will include:

- General photographs of intervention delivery
- Log sheets completed by implementers with details of numbers reached (e.g. head counts at meetings) and any implementation problems (to be recorded by all implementers for all activities, but likely to be discontinued after initial implementation problems have been ironed out).
- Unannounced spot check observations of activity implementation to measure adherence to the intervention in terms of quality of delivery and content (fidelity), and to assess engagement and attentiveness of participants at visits/meetings (reception, acceptability).
- Interviews with implementers (e.g. Health Extension Workers, Stronger SAFE team) to explore ease of delivery and the experience and acceptability of implementing.
- Interviews with intervention recipients to explore reception and comprehension of F&E messages and materials. We will conduct exit interviews immediately following interventions. We will also conduct follow up interviews three months after interventions, with 1 male head of household and 1 female caregiver from one randomly selected household in each intervention cluster, and with prominent members of the community.
- Household survey to collect data on recall and exposure to different elements of the intervention, psychosocial mediators of the intervention i.e. intermediate processes which explain subsequent changes in behaviour change outcomes (most likely Likert-scale ranking of statements around psychological motivations, attitudes, beliefs and norms), spot check observation of presence of intervention collaterals (e.g. posters, soap dishes, fly traps, PTS) and connectedness of participants in each cluster, (e.g. family/social ties). This will be conducted in the same intervention and control households that are visited to measure behavioural outcomes. These data will be collected at baseline, three and 36 months post-intervention.
- To further assess potential for contamination of behaviour change messages and materials between study arms we will record whether there are close family members living in different clusters, and ask *kebele*, *garee*, and 1 to 5 leaders whether the interventions have been discussed at meetings. This data will be collected as part of the F&E behavioural survey.
- Capturing 'context' indicators for each cluster at baseline, including average socioeconomic status, household density, road infrastructure, water sources and functionality, health systems and health extension worker presence and availability, and physical setting for group meetings. Understanding these contextual parameters is important because they can shape what is implemented in an intervention and therefore the effectiveness of interventions. This data will be collected at baseline and updated if necessary.
- Tracking costs of implementation. This will be collected throughout the life of the study.
