# Supplementary material for: Cluster randomised controlled trial of double-dose azithromycin mass drug administration, facial cleanliness and fly control measures for trachoma control in Oromia, Ethiopia: the stronger SAFE trial protocol
Source: BMJ Open. 2024 Dec 23;14(12):e084478. doi: 10.1136/bmjopen-2024-084478 (PMC11751794; doi:10.1136/bmjopen-2024-084478)
Supplement: online supplemental file 10 [file bmjopen-14-12-s010.pdf]

Supplementary 4 Stronger SAFE Trial Timeline

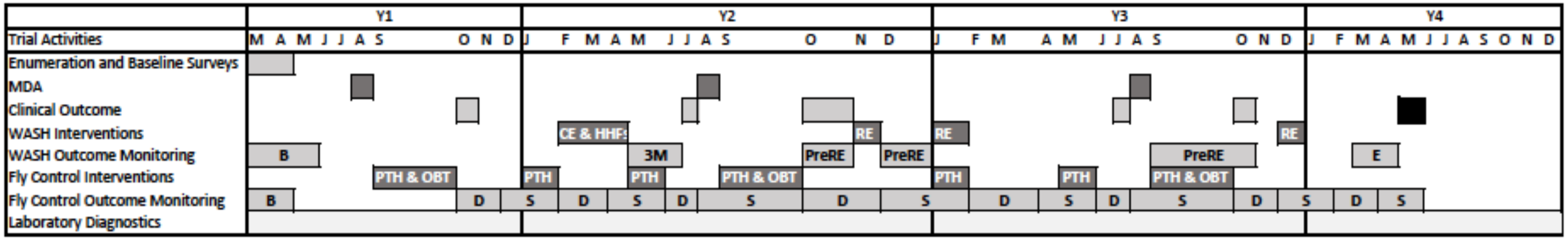

Trial Primary Endpoint Measurement (Cross-sectional prevalence survey in 1-9 year olds)

MDA=Mass Drug Administration; consists of one or two doses (2 weeks apart) of azithromycin (20mg/kg)
Clinical Outcome Surveys are cross-sectional and include trachoma grading and conjunctival swabs
WASH interventions include an initial community event (CE), household forums (HHF) and seasonal reinforcement events (RE)
WASH outcome measures include baseline (B), 3 month (3M), pre-RE spot checks (PreRE) and 36 month endpoint (E) observations
Fly control interventions include distribution of permethrin-treated headwear (PTH) and odour-baited traps (OBT)
Fly control outcome measures include baseline measurements (B) and a rolling schedule every two months of durability (D) and sentinel (S) monitoring
Laboratory diagnostics occur throughout
Y1=2021, Y2=2022, Y3=2023, Y4=2024
