# Supplementary figures and images for: Cluster randomised controlled trial of double-dose azithromycin mass drug administration, facial cleanliness and fly control measures for trachoma control in Oromia, Ethiopia: the stronger SAFE trial protocol
Source: BMJ Open. 2024 Dec 23;14(12):e084478. doi: 10.1136/bmjopen-2024-084478 (PMC11751794; doi:10.1136/bmjopen-2024-084478)

## Supplementary 5 Adverse Event Reporting Flowchart

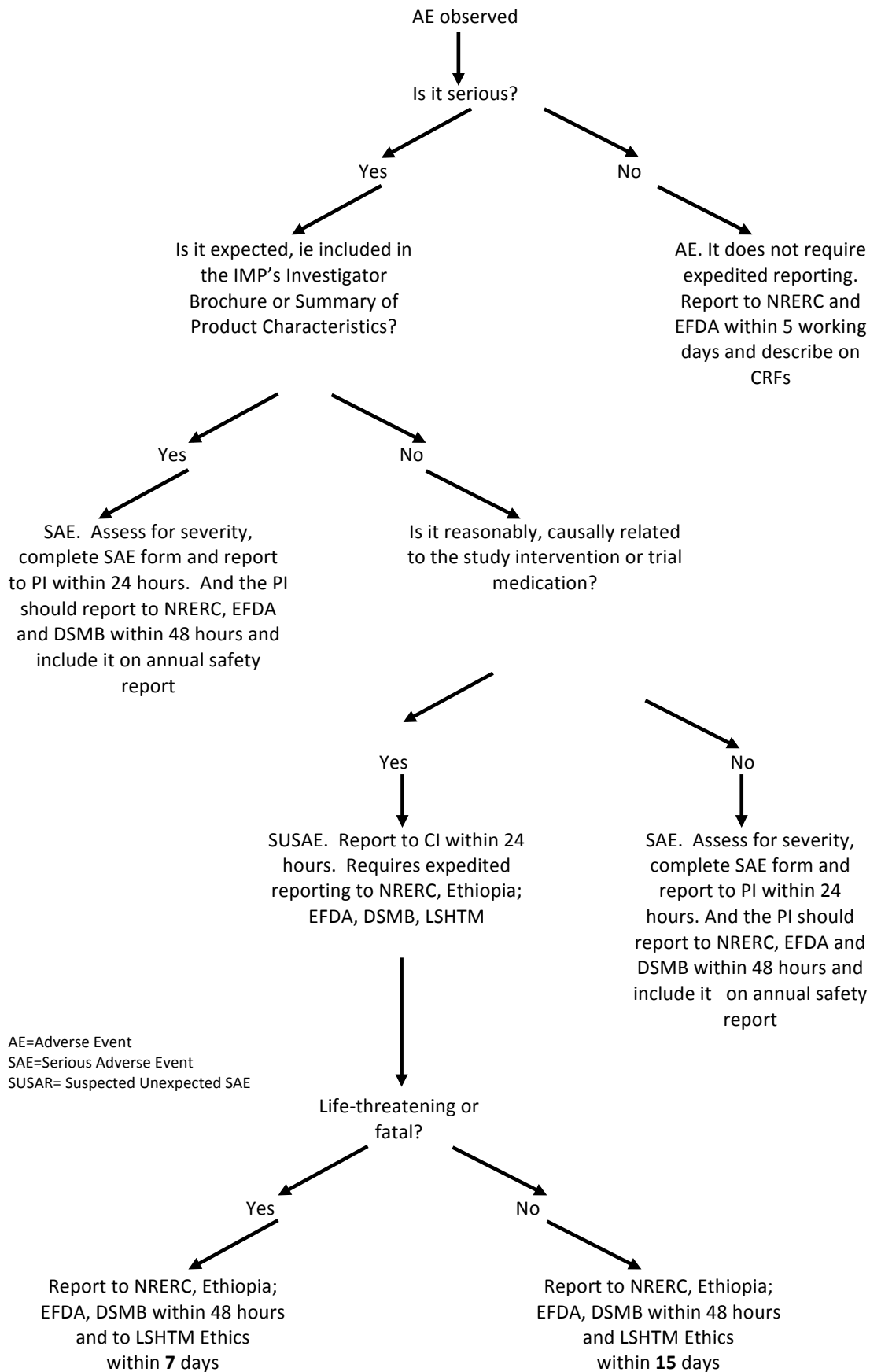

Supplement: online supplemental file 11 [file bmjopen-14-12-s011.pdf]
