# Supplementary material for: Cluster randomised controlled trial of double-dose azithromycin mass drug administration, facial cleanliness and fly control measures for trachoma control in Oromia, Ethiopia: the stronger SAFE trial protocol
Source: BMJ Open. 2024 Dec 23;14(12):e084478. doi: 10.1136/bmjopen-2024-084478 (PMC11751794; doi:10.1136/bmjopen-2024-084478)
Supplement: online supplemental file 12 [file bmjopen-14-12-s012.pdf]

Supplementary 6 DSMB Charter

Full study title: **Stronger Safe: Phase 3 - Cluster-Randomised Trial of Double-dose Oral Azithromycin (A) Combined with Targeted Transmission-Interrupting Strategies (Facial Cleanliness & Environmental Improvement (F&E)) For Trachoma Elimination**

Independent Data Safety Monitoring Board Charter
Version_0.1 Dated: 11/06/2019
(developed using MRC Clinical Trials Unit template DSMB Charter version 2.01, 13-Mar-2006; from DAMOCLES DSMB Charter template v1, Feb 2005)

Authorised by:

Name:	Matthew Burton	Role:	LSHTM Chief Investigator
Signature:	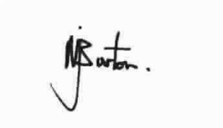	Date:	11/06/2019

Prepared by

Name:	Oumer Shafi	Role:	Ethiopia Principal Investigator
Signature:		Date:	

Abbreviations and glossary

AE	Adverse event
AR	Adverse reaction
CF	Consent form
CHX	Chlorhexidine
CRF	Case Report Form
CTA	Clinical Trials Authorisation
DMC	Data Monitoring Committee
DSMB	Data Safety Monitoring Board
NATA	Natamycin
PI	Principal Investigator
SAE	Serious adverse event
SAR	Serious adverse reaction
SOP	Standard operating procedures
SUSAR	Suspected unexpected serious adverse reaction

DSMB CHARTER FOR STRONGER SAFE- PHASE 3- CLUSTER RCT

Content Guidance	Charter Details
**1. Introduction**	
Name (& Sponsor's ID) of trial	Cluster-Randomised Trial of Double-dose Oral Azithromycin (A) Combined With Targeted Transmission-Interrupting Strategies (Facial Cleanliness & Environmental Improvement (F&E)) For Trachoma Elimination<br><br>***Registration:*** The trial protocol will be registered with the Pan-African Clinical Trials Registry<br>***Sponsor:*** LSHTM<br><br>***Funded by:*** The Wellcome Trust
Ethical Review Boards	Ethiopian National Research Ethics Review Committee<br>Ethiopia Food, Medicine and Health Care Administration and Control Authority<br>London School of Hygiene and Tropical Medicine
Objectives of trial, including interventions being investigated	**Background:** Trachoma, a Neglected Tropical Disease (NTD), is the commonest infectious cause of blindness globally, affecting some of the world's poorest communities. Trachoma is caused by repeated ocular infection with the bacterium *Chlamydia trachomatis* (*Ct*). Active trachoma begins in childhood with recurrent episodes of follicular conjunctivitis (TF). Chronic inflammation results in immunologically mediated conjunctival scarring and in-turned eyelashes scratching the eye: trichiasis. Eventually sight is lost from irreversible corneal opacification.<br><br>Trachoma control requires community-wide measures. The World Health Organization (WHO) Alliance for the Global Elimination of Trachoma by 2020 (GET2020) recommends the SAFE Strategy: **S**urgery for trichiasis, **A**ntibiotic to treat *Ct* infection, **F**acial cleanliness and **E**nvironmental improvements to suppress transmission. Many endemic countries are implementing SAFE, and there has been a major effort to scale up activities, aiming to eliminate trachoma by 2020.<br><br>Currently, the antibiotic component involves mass drug administration (MDA) with oral azithromycin to all community members older than six months. This is given as a single, annual dose, initially for 1-5 years, before reassessing the district-level TF prevalence in 1-9 year olds and deciding whether MDA can be discontinued. The F&E components are much more variable in content and application. If F&E are implemented at all, it usually involves improving water access, sanitation and hygiene (WASH) and fly-control.<br><br>Unfortunately, there is now growing evidence, particularly from hyperendemic regions (>20% TF), that current approaches are not having the anticipated impact on infection and disease. This is a significant threat to the timely elimination of trachoma. Over 44 million live in districts with >30% TF (GTMP data). In hyperendemic areas, current antibiotic schedules appear insufficient to reliably achieve long-term control after treatment completion. In some hyper-endemic areas, more than 18 rounds of single-dose Azithromycin MDA, coupled to standard F & E interventions, have been implemented. However, despite such efforts, the response of trachoma to these control measures has been insufficient to discontinue interventions as the prevalence of TF remains well above threshold suggesting that reliable long-term control is not consistently achieved, with re-emergence of infection being typical.<br><br>It is unknown which, if any, F&E measures, as applied programmatically, suppress *Ct* transmission. There are few randomized-controlled trials in this area, which have demonstrated limited or no effect. Recent Cochrane Reviews of F&E intervention trials concluded there is currently little or no evidence that the tested interventions significantly impacted on trachoma.<br><br>In Stronger SAFE Phase 1, we have been investigating where *Ct* can be detected in addition to eyes infected households. We have found that the skin of the face and hands are the non-ocular locations most frequently found to be positive for *Ct*. This supports the concept that the first step in transmission is on skin. We also found detectable *Ct* on some clothing. These results provide a rational basis for the targeting of interventions to suppress transmission. We also systematically collected, using nets, ten flies leaving the faces of children and tested the presence of Ct in the flies. The data from this study strongly suggest *M. sorbens* is a vector of trachoma.

Content Guidance	Charter Details
	In Stronger-SAFE Phase 2, we tested both commercially available fly traps, and some designed using locally sourced and cheaply available materials. From a number of trap types/designs, we found one commercially available trap (the 'Buzz®') to be successful in catching *M. sorbens*. We have conducted a laboratory-based clinical trial investigating the protection afforded by insect repellent products against *M. sorbens* contact. We exploited the attraction of *M. sorbens* to sweat and measured how this was affected by the presence of repellents. We found that permethrin-treated scarfs (concentrations 102mg or 204 mg active ingredient) may be successful repellent to *M. sorbens.*<br><br>In Stronger-SAFE Phase 3, we would like to investigate whether an enhanced Azithromycin MDA schedule coupled with enhanced F&E interventions can more rapidly and sustainability control ocular *Chlamydia trachomatis* infection transmission.<br><br>**Aim:** To test, in a four-arm cluster randomized trial, the hypothesis that **Stronger SAFE**, comprising of enhanced azithromycin treatment (two single doses of azithromycin two weeks apart) combined with targeted transmission-interrupting strategies, can more effectively control trachoma, determined by measuring the presence of ocular *Chlamydia trachomatis (Ct)* by PCR, than current standard approaches, in a trachoma-hyperendemic region in Ethiopia.<br><br>**Study Design:** Prospective, single-masked, parallel group, four-arm cluster randomised controlled trial.<br><br>**Population:** The trial will be conducted in Oromia, Ethiopia, a region where there is a high prevalence of active follicular trachoma in 1-9-year olds.<br><br>**Participants:** All residents of the 88 community clusters will be offered the antibiotic intervention. The WASH and fly control interventions will apply to certain age groups. The primary outcome measure will be assessed in 8800 children aged 1 to 9 years.<br><br>***Eligibility:***<br>*Inclusion criteria:*<br>• Adults and children<br>• Informed consent and agreement to be randomly allocated to one of the four study arms<br>• Informed ascent for children aged 10+ years being examined for clinical signs of trachoma, conjunctival photos and conjunctival swab collection.<br>*Exclusion criteria:*<br>• Children under six months of age<br>• Illness or incapacity<br>• Inability to communicate<br>• Inability to provide the samples required for the trial<br>• Known hypersensitivity to azithromycin<br>• Known to be pregnant<br>• Confirmed to be taking medications that may cause a serious drug interaction if taken with azithromycin<br><br>**Interventions:**<br>Eighty-eight clusters will be randomly allocated, in a 1:1:1:1 ratio, to one of four intervention arms, with different combinations of antibiotic and F&E:<br>1. Standard Antibiotic / Standard F&E (Control)<br>2. Standard Antibiotic / Enhanced F&E<br>3. Enhanced Antibiotic / Standard F&E<br>4. Enhanced Antibiotic / Enhanced F&E (Stronger SAFE)<br><br>***Antibiotic Interventions:***<br>Oral azithromycin (Pfizer) will be provided by the International Trachoma Initiative. The height-based dosage would follow the standard schedule, 20mg/kg up to a maximum dose of 1g.<br>• <u>*Standard Antibiotic:*</u> single dose, oral azithromycin to all individuals above six months, and topical tetracycline eye ointment twice daily for six weeks for

Content Guidance	Charter Details
	infants under six months. Given for three annual rounds.<br>• *Enhanced Antibiotic:* two doses of oral azithromycin to all individuals above six months, given two weeks apart, and topical tetracycline eye ointment for infants under six months. Given for three annual rounds.<br><br>***F&E Interventions:***<br>• *Standard F&E:* programmes currently promote latrine construction and facial hygiene through health-promotion messages. They also collaborate with the WASH sector to advocate for improved water supply.<br>• *Enhanced F&E Intervention Package:* The additional WASH (Water, Sanitation, Hygiene) interventions comprise an entomological control component and a behaviour change component.<br>The Entomological Control will consist of a 'push-pull' strategy. The "Push" is an insect repellent product permethrin-treated scarf, worn around the neck, that will provide personal protection from eye-seeking flies. The "Pull" involves odour-baited fly traps deployed at the household level, during the hot season only, to reduce local fly population density.<br>The Behaviour Change Package will seek to change facial hygiene practices. The intervention package is designed to be a series of low-cost, sustainable enhancements to the standard F&E package. i) Increasing the perceived value of face washing by adding to or amplifying the motivational drivers associated with face washing. ii) Providing cues in the environment to remind / trigger face washing. iii) Lowering the transaction costs associated with face washing by encouraging the provision of convenient soap and/or water and/or utensils for face washing.<br><br>**Follow-up and Duration:** Clinical assessments and ocular swabs will be collected on seven occasions: Baseline, 2, 12, 14, 24, 26 and 36 months. The anticipated overall project duration is about 3.5 years.<br><br>**Primary Outcome:** the prevalence of ocular *Ct* in children aged 1-9 years determined by *Ct*-specific PCR estimated from samples taken during the endpoint survey at 36 months.<br><br>**Secondary Outcomes:**<br>• Prevalence of TF and TI in children (1-9 years).<br>• Proportion of children (1-9 years) with clean faces.<br>• Fly-control indicators: local (household-level) fly population density and diversity, person-level fly-eye contact in children 2-9 years of age.<br>• Face washing behavioural outcomes: mean daily frequency of good quality face washing among pre-school, school-age children and primary caregivers.<br>• Process indicators to measure exposure to, adherence and recall of the interventions.<br>• Prevalence of malnutrition measured by anthropometry (height and weight for age z-scores) in children (1-60 months).<br>• Prevalence of clinic and hospital visits (specific and all-cause) for children (1-60 months)<br>• Prevalence of diarrhoeal and respiratory illness in children (1-60 months)<br><br>**Laboratory Assays:** One swab sample will be collected from the upper tarsal conjunctiva by the nurse. The DNA from these swabs will be extracted and tested for *Chlamydia trachomatis* by PCR microscopy.<br><br>Whole genome sequence (WGS) data will be obtained directly from clinical samples using DNA baits spanning the length of the *Ct* genome compiled by SureDesign and synthesized by SureSelect^{XT} (Agilent Technologies, UK). DNA will be sequenced at the Wellcome Trust Sanger Institute, UK, using Illumina paired-end technology (Illumina GAII or HiSeq 2000).<br><br>We will determine the nature of the fly gut microbiota using 16s sequencing at the Wellcome Trust Sanger Institute, U,K in a dual-index sequencing strategy to target the

Content Guidance	Charter Details
	bacterial V1V2 16S rRNA gene.<br><br>**Analysis:** CONSORT guidelines for analysing and reporting cluster randomised controlled trials will be followed. A flowchart will show all potentially eligible and eligible participants for the trial, and reasons for exclusion. The number of participants enrolled per cluster per arm will be shown, along with number with outcome data. The baseline characteristics of trial participants will be summarised by arm.<br><br>The primary analysis will be a comparison between the control arm and the arm receiving both enhanced F&E and enhanced MDA. Primary analyses will be by intention-to-treat, with participants analysed according to the arm to which they were randomised and adjustment for factors imbalanced between arms at baseline as appropriate. The intervention effect of Ct prevalence (and other secondary binary outcomes) will be estimated as an odds ratio and 95% confidence interval, estimated using a random effects logistic regression, including cluster as a random effect.<br><br>Primary analysis of behavioural outcomes will be conducted on an Intention-to-treat basis to assess whether the intervention was effective as delivered. Planned subgroup analysis will be done according to water access, level of education and socio-economic status.<br><br>If intervention reach is suboptimal or variable across clusters, per-protocol analyses will be performed to assess whether the intervention succeeded in changing behaviour among those directly exposed to it and subgroup analysis comparing intervention outcomes according to level of intervention exposure will also be performed.
Trial Outline	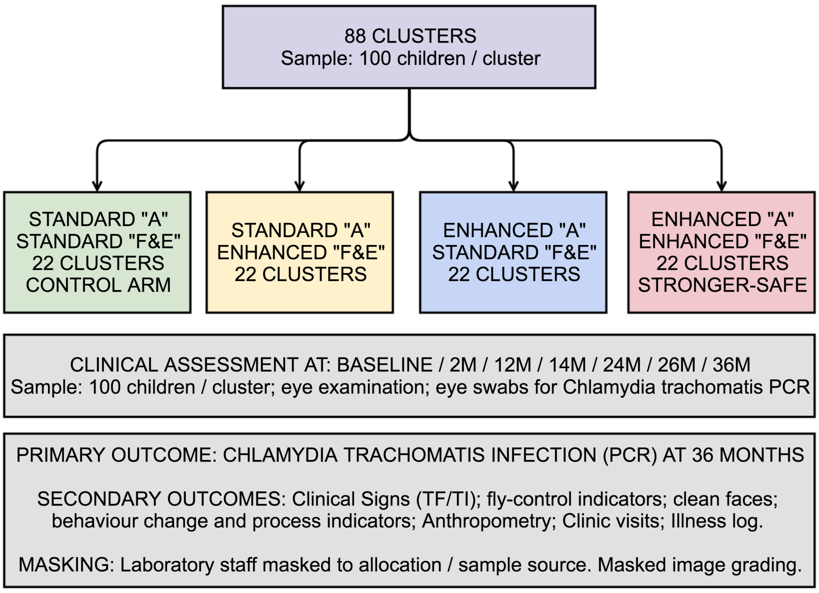
Outline of scope of charter	The purpose of this document is to describe the membership, terms of reference, roles, and responsibilities of the independent DSMB for the this trial.

Roles and responsibilities

A broad statement of the aims of the committee	To safeguard the interests of trial participants, monitor the main outcome measures including safety and efficacy, and monitor the overall conduct of the trial.
Terms of reference	**The DSMB should receive and review information on the progress and accruing data of this trial and provide advice on the conduct of the trial to the**

Content Guidance	Charter Details
	**Principle Investigator (PI), Prof Matthew Burton**
Specific roles of DSMB	Interim review of the trial's progress including updated figures on recruitment, data quality, adherence to protocol treatment and follow-up, and main outcomes and safety data. Specifically, these roles include to:<br>• monitor evidence for any differences in the outcome measures by treatment arm<br>• monitor evidence for treatment harm (e.g. SAEs and SARs, deaths)<br>• assess the impact and relevance of external evidence (eg results from other trials or studies if any)<br>• decide whether to recommend that the trial continues to recruit participants or whether recruitment should be terminated either for everyone or for some treatment groups and/or some participant subgroups<br>• decide whether trial follow-up should be stopped earlier<br>• assess data quality, including completeness (and by so doing encourage collection of high quality data)<br>• maintain confidentiality of all trial information that is not in the public domain<br>• monitor enrollment figures and losses to follow-up<br>• monitor compliance with the protocol, including adherence to interventions, by participants and investigators<br>• suggest additional data analyses if necessary<br>• advise on protocol modifications proposed by investigators or sponsors (e.g. to inclusion criteria, trial endpoints, or sample size)<br>• monitor compliance with previous DSMB recommendations
3. **Before or early in the trial**	
Whether the DSMB will have input into the protocol	All DSMB members have seen the protocol before agreeing to join the committee.<br>All ethical and relevant regulatory permissions by funder trial sponsor, local research ethics committee (REC) and drugs and pharmacy boards have been successfully approved before the start of recruitment.<br>Reservations by DSMB members about the trial should be raised with the PI, but while DSMB members should be independent and constructively critical of the ongoing trial, but also supportive of aims and methods of the trial.
Timing of the DSMB first meeting	The DSMB will first meet before, or soon after the start of the trial
DSMB contract	DSMB members should formally register their assent to join the group by signing Annex A, which shows that they agree to be on the DSMB and that they agree with the contents of this Charter.<br>Any competing interests should be declared in writing to the PI and the Chair of the DSMB.
**4. Composition**	
Membership of the DSMB	The members of the DSMB for this trial are:<br>Dr Teshome Gebrie, Consultant Health Sciences Specialist (Chair)<br>Dr John Kempen, Senior Ophthalmologist, Epidemiologist, and Clinical Trial Specialist<br>Dr Nyawira Mwangi, Senior Ophthalmologist & Researcher<br>Dr Stephen Gichuhi, Senior Ophthalmologist & Clinical Trial Specialist<br>Dr Alemayehu Teklu, Paediatrics and Child Health Specialist (Safety Officer)
DSMB Chairman: choice and role.	The Chair has been nominated by the PI, and has previous experience of serving as the chair of DSMB, and will be able to facilitate and summarise discussions.
DSMB Safety Officer: choice and	The Safety Officer has been nominated by the PI, and has the necessary clinical

Content Guidance	Charter Details
role.	experience, and will be able to particularly overlook AEs.
**5. Relationships**	
Relationships with PI and other trial investigators, sponsors and regulatory bodies.	The DSMB is independent of all other regulatory bodies, and of the trial personnel.<br>If required, the reports from the DMC will be sent to the ethics committees or other regulatory authorities.
Payments to DSMB members	Members will be reimbursed for reasonable travel costs and accommodation where required.<br>No other payments or rewards are given.
Need for DSMB members to disclose information about any competing interests	Competing interests should be disclosed. These disclosures are not restricted to financial matters – but could include involvement in other trials or intellectual investment in the research area.<br>DSMB members should not use interim results to inform trading in pharmaceutical shares, and careful consideration should be given to trading in stock of companies with competing products.
**6. Organisation of meetings**	
Expected frequency of DSMB meetings	The DSMB will meet every six months until completion of the study.<br>Meetings will be held by videoconference or face-to-face in Addis Ababa unless agreed beforehand
Organisation of the DSMB meetings	A mixture of open and closed sessions will be held.<br>Closed sessions will only be attended by DSMB members, but may invite, any expert in another field to advise them.<br>In open sessions, all those attending the closed session may be joined by the PI and other members of the trial, at the PIs discretion.
**7. Trial documentation and procedures to ensure confidentiality and proper communication**	
Content of material to be available in open sessions	Accumulating information relating to recruitment and data quality (e.g. data return rates, protocol and treatment compliance) will be presented.<br>Total numbers of events for the primary outcome measure and other secondary outcome measures will be presented (but not be summarised by treatment group)<br>SAE's and/or toxicity details based on pooled data will be presented and total numbers of events for the primary outcome measure and other outcome measures may be presented, at the discretion of the DSMB.
Content of material to be available in closed sessions	In addition to all the material available in the open session, the closed session material will include efficacy and safety data by treatment group.
Preparation and use of the reports	Materials for both the open session and the closed session of the DSMB will be prepared by the trial statistician, Dr David MacLeod, and circulated to the DSMB members 2 weeks prior to the DSMB meeting.<br>Any material showing accumulating data and analysis by randomised group will available only to the DSMB members, and will be kept confidential.<br>DSMB members do **not** have the right to share confidential information with anyone outside the DSMB, including the PI.
Responsibility for identifying and circulating external evidence	Identification and circulation of external evidence (e.g. from other trials/ systematic reviews) are not the responsibility of the DSMB members, although they will be able to bring in any such information should they come across it.<br>The PI and the trial management team will collate any such information for presentation in an open session
To whom the DSMB will report	The DSMB reports its recommendations in writing to the PI.
What will happen to the confidential papers after the	The DSMB members should destroy their reports after each meeting.

Content Guidance	Charter Details
meeting?	The DSMB members should store any of their own notes and papers safely to ensure information about the trial outcomes by treatment arm does not inadvertently come to the attention of trial personnel.
**8. Decision making**	
What decisions/recommendations will be open to the DMC?	Possible recommendations from the DSMB include:-<br>• No action needed, trial continues as planned<br>• Early stopping due, for example, to clear benefit, or clear lack of benefit or harm of a treatment, or due to external evidence.<br>• Extending recruitment (based on actual control arm response rates being different to predicted rather than on emerging differences)<br>• Extending follow-up<br>• Proposing or commenting on proposed protocol changes<br>• Commenting on Statistical Analysis Plan
How decisions or recommendations will be reached within the DSMB	The Chair is to summarise discussions and encourage consensus; it is usually best for the Chair to give their own opinion last.<br>Every effort should be made for the DSMB to reach a unanimous decision. If the DSMB cannot achieve this, a vote may be taken, although details of the vote should not be routinely included in the report.<br>It is important that the implications (e.g. ethical, statistical, practical, financial) for the trial be considered before any recommendation is made.
When the DSMB is quorate for decision-making	Efforts should be made to ensure that all members can attend.<br>**Quorate for decision- making:** If, any DSMB members cannot attend then the DSMB will be quorate providing three members are present.
Can DSMB members who cannot attend the meeting input	If the report is circulated before the meeting, DSMB members who will not be able to attend the meeting may pass comments to the DSMB Chair for consideration during the discussions.
**9. Reporting**	
To whom will the DSMB report their recommendations/decisions, and in what form.	The DSMB should report in writing to the PI, usually within 2 weeks after the meeting. The report should be careful not to include any information about the arms of the trial.
Minutes of DSMB meetings, by whom and where they will be kept	Minutes from the meeting need not be detailed. A summary of the main points discussed with a list of clearly marked action points should be sufficient.<br>The person who will take the minutes for the session should be agreed at the start of the meeting**.** All members of the DSMB should see and comment on the minutes before release. The DSMB Chair should sign off any minutes or notes.
**10. After the trial**	
Publication of results	At the end of the trial there may be a meeting of the DSMB to discuss the final data.<br>The DSMB may be given the opportunity to read and comment on publications before submission, if the PI considers this necessary.
The information about the DSMB	DSMB members will be acknowledged by name and their affiliations listed in the main report, unless they explicitly request otherwise.
Any constraints on DSMB members divulging information about their deliberations after the trial has been published	DSMB members should refrain from discussing confidential issues from the Closed meeting.

Annexe 1: Agreement and potential competing interests form

Stronger Safe: Phase 3 - Cluster-Randomised Trial of Double-dose Oral Azithromycin (A) Combined with Targeted Transmission-Interrupting Strategies (Facial Cleanliness & Environmental Improvement (F&E)) For Trachoma Elimination

Agreement to join the Independent Data Monitoring Committee and disclosure of potential competing interests

Please complete the following document and return to the PI.
(please initial box to agree)

☐	I have read and understood the DSMB Charter version 0.1 dated 11/06/2019
☐	I agree to join the Independent Data Monitoring Committee for this trial
☐	I agree to treat all sensitive trial data and discussions confidentially

The avoidance of any perception that members of a DSMB may be biased in some fashion is important for the credibility of the decisions made by the DSMB and for the integrity of the trial.

☐ **No,** I have no competing interests to declare
☐ **Yes,** I have competing interests to declare (please detail below)

Please provide details of any competing interests:

____

____

____

Name: ___________________________

Signed: __________________________ Date: ______________

Table 1: Potential competing interests

- Stock ownership in any commercial companies involved
- Frequent speaking engagements on behalf of the intervention
- Career tied up in a product or technique assessed by trial
- Hands-on participation in the trial
- Involvement in the running of the trial
- Emotional involvement in the trial
- Intellectual conflict e.g. strong prior belief in the trial's experimental arm
- Involvement in regulatory issues relevant to the trial procedures
- Investment (financial or intellectual) or career tied up in competing products
- Involvement in the publication in the form of authorship

DSMB Membership

Teshome Gebre Kanno (Chair)
Regional Director for Africa, International Trachoma Initiative
Task Force for Global Health, Addis Ababa, Ethiopia

Alemayehu Teklu Toni
Assistant Professor in Paediatrics
University of Gondar, Ethiopia

John Kempen
Professor in Clinical Epidemiology and Ophthalmology
Faculty of Medicine, Harvard University, Boston MA, USA

Stephen Gichuhi
Senior Lecturer in Ophthalmology
University of Nairobi, Kenya

Nyawira Mwangi
Principal Lecturer and Medical Specialist
Kenya Medical Training College, Kenya

Stronger SAFE

Standard Operating Procedure

Stronger SAFE Steering Committee Terms of Reference	
SOP Ref:	Stronger SAFE/SOP/01
Version:	1.0
Authors:	Anna Last / Matthew Burton
Effective Date:	
Review by:	Anna Last, Matthew Burton, Anthony Solomon.
Approved by:	Stronger SAFE Steering Committee
Approval Date:	
Signed by:	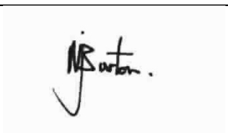 Matthew Burton

Version	Date	Reason for Change
1.0	16/06/2017	N/A
1.1	5/07/2021	Reviewed and updated ahead of first meeting

Stronger SAFE Steering Committee

Terms of Reference

1. Purpose:

The Stronger SAFE Steering Committee will provide formal oversight of the Cluster Randomised Controlled Trial, which is being conducted in Phase 3 of the Stronger SAFE programme of research. This complements the work of the Project Management Group, which meets weekly to coordinate scientific and logistic matters arising as the trial progresses.

2. Membership:

The Stronger SAFE Steering Committee members are people who are directly involved in the work and people who are independent. There are both "Voting Members" and "Observer Members".

**"Voting Members"**	
Matthew Burton	LSHTM (Principal Investigator (PI))
Fikre Seife	Federal Ministry of Health, Ethiopia
James Logan	LSHTM
Virginia Sarah	FHF-UK
Oumer Shafi	FHF-E / LSHTM
Anthony Solomon (Chair)	WHO
Helen Weiss	LSHTM
Esmael Habtamu	LSHTM and University of Bahir Dar
Steven Lindsay	University of Durham
Yael Velleman	Schistosomiasis Control Initiative
Anna Last	LSHTM
**"Observer Members"**	
Katie Greenland	LSHTM
David Macleod	LSHTM
Sarah O'Regan	LSHTM (Project administrator / Minute Secretary)
Ailie Robinson	LSHTM
Robert Butcher	LSHTM
Wellcome Trust	Wellcome Trust representative

Anthony Solomon is Chair of the Steering Committee. Should he be unable to perform the duties of Chair, the Chair or the members of the Steering Committee will appoint a temporary Chair.

The coordinators of the different sections will be invited to attend the meetings as Observer Members to be able to provide reports and participate in discussion relating to the section they coordinate. The project administrator will be responsible for recording the minutes.

3. Responsibilities:

Responsibilities and key areas of activity of the Stronger SAFE Steering Committee are to:

- Assess the progress of the research programme against primary objectives, timeline and budget
- Undertake periodic protocol review and propose and/or review amendments with the PI
- Review the biannual reports from the programme sections
- Provide advice to the PI / Co-investigators should there be a risk of significant variation from the proposed schedule
- Provide advice to the PI / Co-investigators on major scientific components and logistical matters

The London School of Hygiene & Tropical Medicine (LSHTM) holds the primary contractual responsibility for the delivery of the Stronger SAFE programme. This contract is governed by the Wellcome Trust Grant Terms and Conditions.

Collaboration Agreements have been set up between the LSHTM and partner organisations participating in the programme, which will also be governed by the Wellcome Trust Grant Terms and Conditions.

The overall management responsibility rests on the Principal Investigator, with delegated responsibilities through the co-investigators and the administration team encompassing all the sections of the research programme.

4. Reporting to the Steering Committee:

- The PI and Co-investigators will report to the Steering Committee on the progress of the Stronger SAFE programme.
- The PI and Co-investigators will work with the Chair of the Stronger SAFE Steering Committee to facilitate the discussions of and implementation of recommendations from the Stronger SAFE Steering Committee by:
    - Arranging meetings of the Stronger SAFE Steering Committee
    - Providing additional documents or reports as requested by the Chair of the Stronger SAFE Steering Committee
    - Recording discussions of the Stronger SAFE Steering Committee
    - Promptly distributing minutes of meetings of the Stronger SAFE Steering Committee,

The PI will additionally report annually to the Wellcome Trust on behalf of Stronger SAFE.

5. Steering Committee Meetings:

5.1. Frequency

- The Chair will convene a meeting of the Stronger SAFE Steering Committee twice a year, with dates planned at the preceding meeting. A minimum of 30 working days notice will be provided if there is a change of date required.
- Additional meetings can be called by the PI or Chair if necessary.

5.2. Attendance and Quorum

- Members of the Stronger SAFE Steering Committee may participate in person, by video or by teleconferencing as appropriate.
- A quorum is a simple majority of the Stronger SAFE Steering Committee.

5.3. Agenda

- The agenda will be prepared by the PI and Chair.
- Each Steering Committee Member and listed observers have the right to propose items for the agenda
- The agenda and all committee documents will be distributed among all members at least five working days before the meeting by the administration team.

5.4. Decisions and recommendations

- Decisions / recommendations from the Stronger SAFE Steering Committee should be taken by consensus, where consensus is defined as the situation in which no member of Stronger SAFE Steering Committee expresses a desire to prevent the decision or recommendation being adopted.
- In case of failure to reach consensus, any Voting Member may call upon the Chair for a vote to be taken, and the decision will be included in the minutes.
- All Voting Members attending the meeting (including by conference / video call) are entitled to one vote; there is no right of proxy vote for absentee members.
- All decisions / recommendations put to vote require a simple majority of votes; in the event of a tie the Chair has the casting vote.

5.6. Minutes

- Meetings held virtually will be recorded for record keeping purposes
- The minutes will be taken by the project administrator

- The minutes will include the date, participants, agenda and a record of recommendations / decisions made
- The minutes will be circulated to each member within 10 working days after the meeting
- The PI and Chair will agree the minutes and then circulate by email to all other committee members for review and approval,
- The minutes will be confirmed and circulated by email (assuming an absence of objection or amendment) 10 days after circulation.

6. Confidentiality:

All information presented to the Stronger SAFE Steering Committee is confidential and should not be disclosed to third parties.

7. Term

These Terms of Reference are effective from 5th July 2021 and will remain in force until termination of the agreement between parties (likely to coincide with completion of the programme/ultimate publication arising from the programme).

8. Amendment, Modification or Variation

These Terms of Reference may be amended, varied or modified in writing after consultation and agreement between the Voting and Observer Members of the Stronger SAFE Steering Committee.

Date: 5th July 2021
