# Supplementary material for: Cluster randomised controlled trial of double-dose azithromycin mass drug administration, facial cleanliness and fly control measures for trachoma control in Oromia, Ethiopia: the stronger SAFE trial protocol
Source: BMJ Open. 2024 Dec 23;14(12):e084478. doi: 10.1136/bmjopen-2024-084478 (PMC11751794; doi:10.1136/bmjopen-2024-084478)
Supplement: online supplemental file 13 [file bmjopen-14-12-s013.pdf]

Stronger SAFE
Eliminating Trachoma

APPENDIX 4A: INFORMATION SHEET – MAIN TRIAL & CLINICAL OUTCOMES

Federal Ministry of Health, Ethiopia
Fred Hollows Foundation, Ethiopia
Oromia Regional Health Bureau, Ethiopia
London School of Hygiene and Tropical Medicine, UK

Stronger SAFE: Phase 3 – Cluster-randomised trial of double-dose oral azithromycin combined with targeted transmission-interrupting strategies for trachoma elimination in Ethiopia

INFORMATION SHEET – MAIN TRIAL & CLINICAL OUTCOMES

What is the purpose of this study?
Trachoma is an eye disease caused by a germ called Chlamydia that many people catch in Ethiopia. It can cause people to go blind. Trachoma is transmitted from eye to eye in a number of different ways. We believe we can help prevent trachoma by giving everyone in the community antibiotics to treat infection, combined with measures designed to stop trachoma from spreading between people. These include personal hygiene and fly control measures. We are interested in studying the best ways to remove trachoma from your community by implementing double-dose (two weeks apart) antibiotic treatment. We hope that we will be able to use the information we learn from this study to improve trachoma control in Ethiopia.

What will I be asked to do if I participate in these studies?
(Field Worker to tick the box of sections relevant to each participant.)

☐ **Household census and household survey**
Should you agree to participate, we will record the names, ages and gender of all the people who live in your household at the beginning of the study. We will give you individual identification numbers. You should keep these safe. We will look at your house and compound and note things like the materials, number of rooms and latrines. We will ask you questions about your occupation, religion, education, and wealth, and about your daily life, for example how you collect and use water in your household. We will come back later in the study to check if there have been changes to your household, for example births or deaths.

☐ **Eye and face examination with digital photographs and conjunctival (eye) swabs**
Should you agree to participate; a nurse will examine both eyes in certain children aged 1-9 years in your household. We will take a photo of each eye that we examine using a special camera. We will also take one sample with a cotton swab to test for the presence of the trachoma germ (Chlamydia). These swabs will be tested in laboratories in Ethiopia and the UK. The examination takes a few minutes to complete. We will put a drop of anaesthetic medicine on the surface of the eye to avoid any discomfort while taking the swab sample. This examination has been carried out in many countries including Ethiopia without any problems. Our researcher will observe the faces of you and certain children aged 1-9 years. They will take a photograph of the child's face. They will then wipe each forehead with a cloth wipe and will take a photograph of this wipe. We will look at the amount of dirt or dust that comes off on to the wipe. This is not uncomfortable and will not cause any pain.

☐ **Azithromycin**

Your entire household will be given one or two doses of treatment with the antibiotic tablet (or syrup), Azithromycin, every year. This is the same antibiotic that is usually given in this community, and you will probably have been offered this before. The decision on whether your household will receive one or two doses will be random (as if picked out of a hat). If you receive two doses these will be given about two weeks apart. The antibiotic tablets can occasionally cause some mild stomach ache a few hours after taking them. We do not expect that you will experience any serious side effects from azithromycin but you will receive instructions from the team about who to contact if you have any concerns or questions after taking the treatment.

☐ **Household hygiene and fly control**

Your household might be given some support to improve personal hygiene and to reduce nuisance from flies. The decision on whether your household will receive this support will be random (as if picked out of a hat). You might receive:

(1) **A fly trap**: flies are attracted to a chemical that makes them go inside the trap, where they die. Traps are designed to be built using locally-available materials, so that you can build and look after their own trap. We will provide these materials and training at the beginning of the dry/hot season. However, if you need to replace parts of the trap during the hot/dry season when you are using it, you may need to replace those yourself. We will provide the chemical that attracts the flies.

(2) **A scarf or cap ('repellent headwear')**: these items contain a chemical that flies do not like, called "Permethrin". It may stop flies going to children's eyes. The repellent headwear is for children aged between two and nine years old, as these children are the most bothered by flies and the most at risk of trachoma. Children in this age group should be encouraged to wear the repellent headwear when they are outside, and they are being bothered by flies. The amount of permethrin in the repellent headwear is low and safe for children to use. However, children should be discouraged from putting the repellent headwear into their mouth, eyes, or nose.

(3) **Visits to your household or community** from a health worker or a member of our team. They will share information, messages and materials about hygiene.

General Information

Why have I been selected for this study?

You have been invited to participate in this study because the area you live in has trachoma.

How long is this study and how often will I need to participate?

This study visit will take approximately 5 minutes per child to complete. Following this visit, we might visit you between one and seven times over the next three years to examine children's eyes and faces. These subsequent visits will take less than one hour. If your household receives hygiene and fly control measures, we will visit you more frequently, especially at the beginning of the study and during the dry season. We will visit before the dry season starts, to help you set up the fly trap, and we may visit more often over the dry season to check if you need help or advice about the trap. We will also visit your community every two to four months for information sessions or events about these interventions. If you choose to participate in the study initially you do not need to continue to participate on other occasions if you do not want to.

Where are these studies taking place?

This research is taking place in communities in Oromia. Around 88 communities will participate in the study. After the first round of data collection we will select a random sample of children aged 1-9 years in each community.

Do I have to take part?

No. Your participation is voluntary. Our researcher will help you understand this form and answer your questions. It is up to you to decide to take part or not. If you don't want to take part, that's ok. You can withdraw from any part of the study at any time, for any reason. If you do agree, you are still free to withdraw yourself and your family at any time without any consequences to you or your family. Should you withdraw from the study, you will continue to obtain the regular benefits of any health care services you normally get at the clinic. Should you choose to participate now, you do not need to participate again in a future round of data collection.

What are the possible benefits to being in these studies?

Research is designed to benefit society by contributing new knowledge which will help shape future health programs. You may, however, receive no direct benefit from the study. As part of the assessment we will examine your eyes and if any significant problems are identified we will arrange for you to be referred for help. Azithromycin medicine has been shown to be effective at reducing the amount of eye infection, in addition it has been shown to reduce illness and deaths in young children. If your house receives personal hygiene and fly control measures, it is possible that there will be fewer flies in and around your house, and it is possible that while your child/children use the repellent headwear, they may experience less irritation from fly contact to their face. Improved personal hygiene might lead to you and your children experiencing fewer illnesses.

What could go wrong?

Our researchers are trained to respect your emotions. The collection of the eye swabs is associated with a few seconds of mild discomfort. Azithromycin is a safe and well-tolerated antibiotic that has been used in Ethiopia (several hundred million doses) and many other countries in the world for this purpose. There should be no risks associated with changing personal hygiene habits or any of the items you might receive for your home, but our team will explain what you should do if you do experience any discomfort.

If your house receives fly control measures, there is a small risk that your child will experience skin irritation or a reaction during or after wearing the repellent headwear. If this happens, please immediately take it from them, wash the area with soap and water, and contact someone in our team. If this happens to your child, they will be withdrawn from the study. Because the repellent headwear has a chemical in it, it is best that these items don't contact the eyes, nose or mouth of the child, as this may not be good for them. However, even if this happens, it is unlikely that it will lead to problems. Flies caught in the fly traps present a small risk to people, as flies are unhygienic. Therefore, only those who have been trained in using the trap should touch it. The lure inside the trap does not contain bad chemicals, it is composed of food-based substances including yeast. For these hygiene reasons, it is important that children are discouraged from touching the trap and specifically the lure (bait) or the flies trapped inside.

If you feel uncomfortable with the researcher being in your house or carrying out any of the activities you should inform them immediately or ask to speak to their supervisor, who will do his best to answer your questions (Mr Oumer Shafi, Tel: +251912048181). The London School of Hygiene and Tropical Medicine holds insurance policies which apply to this study. If you experience harm or injury as a result of taking part in this study, you may be eligible to claim compensation.

What will happen to the information you collect?

All information collected about you, including videos/photos taken and geolocations, will be kept private and secure on password-protected computers or in locked cabinets. Only the people organising the study will have access to it. Reports and presentations summarizing the information collected in the study may be made publicly accessible. However, we will not include your name or any personal details that could identify you as a participant in any information we publish about the study. The photographs of faces and face swabs will mainly be used for independent verification, and the people verifying will not know your name. If you give us permission, the face swabs, photographs of these swabs and photographs of your

family's faces will be used to support other research in the future, and may be shared anonymously with other researchers, for their ethically-approved projects.

What if I still have questions about this study or my rights as a participant?
All research on human volunteers is reviewed by both the National and Oromia Health Bureau Ethical Review Board and LSHTM Research Ethics Committee that works to protect your rights and welfare. You have the right to ask, and have answered, any questions you may have about this research and your participation. If you have questions, complaints, or concerns please contact Mr Oumer Shafi.

Who is carrying out this study?
This study is being conducted through a partnership between the Ethiopian Federal Ministry of Health, the Oromia Regional Health Bureau, the Fred Hollows Foundation Ethiopia and the London School of Hygiene & Tropical Medicine, UK. The London School of Hygiene & Tropical Medicine will act as the trial sponsor. The trial is funded by the Wellcome Trust (UK).

Further information: If you would like any further information, please contact

Contact Information
If you have any questions please ask us:

- if you have any questions about this study or your part in it,
- if you feel you have had a research-related injury or an adverse effect from the surgery or the drug,
- if you have questions, concerns or complaints about the research

Mr Oumer Shafi Tel: +251 91 204 8181.
Prof. Matthew Burton at +44 20-7636-8636 or matthew.burton@lshtm.ac.uk

NRERC: NRERC Secretariat
Tel: +251118720943
Email: nrerc2019@gmail.com

EFDA: Medicine Registration and License Directorate
Tel: 00251-1524122/524123
Email: efmhacapharmacovigilance@gmail.com
Email: regulatory@fmhaca.gov.et

You will be given a copy of the information sheet and a signed consent form to keep.
Thank you for considering taking the time to read this sheet.

APPENDIX 4B: CONSENT FORM – MAIN TRIAL & CLINICAL OUTCOMES

Federal Ministry of Health, Ethiopia
Fred Hollows Foundation, Ethiopia
Oromia Regional Health Bureau, Ethiopia
London School of Hygiene and Tropical Medicine, UK

Stronger SAFE: Phase 3 – Cluster-randomised trial of double-dose oral azithromycin combined with targeted transmission-interrupting strategies for trachoma elimination in Ethiopia

CONSENT FORM – MAIN TRIAL & CLINICAL OUTCOMES

TO BE COMPLETED BY ALL ADULTS IN ALL STUDY HOUSEHOLDS

Stronger SAFE: Phase 3 – Cluster-randomised trial of double-dose oral azithromycin combined with targeted transmission-interrupting strategies for trachoma elimination in Ethiopia

Statement	Please initial or thumbprint* each box
I have read/been read the information provided above and I have understood it. I understand the activities that are to take place, and I have asked all the questions I have at this time.	
I give permission for researchers to visit my household at up to seven other occasions over the next three years to perform eye and face examinations of my children aged 1-9, with conjunctival (eye) swabs.	
I understand that everyone in my household will be offered one or two doses of treatment with the antibiotic tablet (or syrup), Azithromycin, every year, and that we will record whether or not you take this.	
I understand that if I receive fly control measures, I will be given the materials and shown how to build a fly trap, and I will be asked to maintain the fly trap. I understand that the research team may visit up to once per month during the dry season (Dec-March) to answer any questions about the trap.	
I understand that if I receive fly control measures, children in this household aged 2-9 will be given repellent headwear (scarf or cap) to wear to protect them against flies. I understand that this repellent headwear contains a chemical that makes flies go away, but the repellent headwear should not be given to children aged less than two years.	
I understand that if I receive hygiene activities, my home and community will be visited on several occasions, and I might be given information, messages, or household items to make it easier to maintain personal hygiene.	
I understand that it is my right to withdraw from the study at any time without giving any reason, without my medical care or legal rights being affected.	
I understand that data collected during the study may be looked at by authorised individuals from the London School of Hygiene and Tropical Medicine, Fred Hollows Foundation, I give permission for these individuals to have access to my records.	
I understand that data/photos may be shared via a public data repository or directly with other researchers, and that I will not be identifiable from this	

Stronger SAFE
Eliminating Trachoma

information.	
I give permission for my data to be reported anonymously to communicate the findings of this research, to analyse this research, and for teaching purposes. I understand that samples and information collected in this study could potentially be seen by researchers and students in the UK and beyond, and by health professionals and decision-makers in Ethiopia/UK and beyond.	
Primary caregiver only: I give my consent for all household members under 18 for whom I am the parent or guardian to participate in the study.	

Photos and videos of me (and my children if primary caregiver) may be taken to document the research (circle one):
Yes **No**

**I give permission for photos and videos to be used as follows:**	**YES** ✓	**NO** ✗
As part of this study report		
In other reports, campaigns and publications by LSHTM or affiliated partners and donors		
On the LSHTM website or in other media about this study		
As part of a public data repository		

____
Signature / Thumbprint of Participant Name Date

____
Signature of Researcher Name Date

____
Signature of Impartial Witness* Name Date
***Note to researcher: Witness signature and date are required on this consent form only when the consenting volunteer is not able to read (illiterate). The researcher may be able to sign their name but still require a witness.**

Stronger SAFE
Eliminating Trachoma

ASSENT FORM – MAIN TRIAL & CLINICAL OUTCOMES
TO BE COMPLETED BY INDIVIDUALS AGED 10-17 YEARS IN ALL HOUSEHOLDS

Stronger SAFE: Phase 3 – Cluster-randomised trial of double-dose oral azithromycin combined with targeted transmission-interrupting strategies for trachoma elimination in Ethiopia

Your parents / guardians have agreed for your household to part of a research study, but I also need to ask you if you are happy to take part. The study is to learn more about trachoma and how we can best deliver antibiotics to prevent it from spreading from the eye of one person to the eye of another person.

If you agree to take part then researchers may wish to visit your household on several occasions over the next 3 years. They will record your name and age, and record whether you take the antibiotics we give you. They might ask to take photos of you and your home and might talk to you about hygiene. You do not have to take part in the study if you do not want to even though the researchers will be in your home. You can ask me any questions about the study now. If you are willing to take part, I need to ask you to write your name or make your thumb print on this sheet to show you have given your permission.

I, ______________________________ have read/been read the information provided above and I have understood it. I have asked all the questions I have at this time. I understand that it is my right to withdraw from the study at any time without it affecting me or my family. I understand that the information/photos/swab sample collected from me will be used to support other research in the future, and may be shared anonymously with other researchers, for their ethically-approved projects.

I am willing to take part in this study (tick one box). ☐ **Yes** ☐ **No**

**I give permission for photos of me (not named) and anonymised results to be used in the following ways:**	**YES** ☑	**NO** ☒
As part of this study report		
In other reports, campaigns and publications by LSHTM or affiliated partners and donors		
On the LSHTM website or in other media about this study		
As part of a public data repository		

____
Signature / Thumbprint of Participant Name Date

____
Signature of Researcher Name Date

____
Signature of Impartial Witness* Name Date

***Note to researcher: Witness signature and date are required on this consent form only when the consenting volunteer is not able to read (illiterate). The researcher may be able to sign their name but still require a witness.**

APPENDIX 4C: INFORMATION SHEET – ENTOMOLOGY IN SENTINAL HOUSEHOLDS - HEAD

Federal Ministry of Health, Ethiopia
Fred Hollows Foundation, Ethiopia
Oromia Regional Health Bureau, Ethiopia
London School of Hygiene and Tropical Medicine, UK

Stronger SAFE: Phase 3 – Cluster-randomised trial of double-dose oral azithromycin combined with targeted transmission-interrupting strategies for trachoma elimination in Ethiopia

INFORMATION SHEET – ENTOMOLOGY IN SENTINAL HOUSEHOLDS - HEAD

Introduction
We would like to invite you to take part in a research study. Joining the study is entirely up to you. Before you decide, you need to understand why the research is being done and what it would involve. One of our team will go through this information sheet with you, and answer any questions you may have. Ask questions if anything you read is not clear or you would like more information. Please feel free to talk to others about the study if you wish. Take time to decide whether or not to take part.

What is the purpose of the study?
We have approached your household because you are already enrolled in the Stronger-SAFE project. We are asking a small number of households in the Stronger SAFE study if we can conduct some more detailed observations on flies around their home.

What will happen to me if I take part?

☐ **Sentinel households (repellent headwear and trap)**

To study flies we need to take measurements regularly, because the number of flies around your house can change very quickly. **Therefore, we will need to visit your house every six to eight weeks over the next three years.** This visit will take no more than 30 minutes, we will contact you in advance to organise a convenient time with you. It will be necessary that your children aged 2 to 9 years are at home while we visit.

During the visits, you and your child will need to be available at your house. The researchers will ask your child to sit on a chair, wearing their repellent headwear (scarf/cap) if they were given this. If they did not receive this, the child will be asked to wear headwear which we will supply at this time. The researchers will then observe the flies on your child's face. They will also video and photograph their face so that we can analyse fly behaviour later. They will record the weight and body temperature of your child.

During the dry season, we will visit on two consecutive days, but again each visit will be no more than 30 minutes. The researchers will place a fly trap outside your home. If you already received a fly trap as part of the Stronger-SAFE study, they will just use the trap that is already there. They will empty the trap and return in 24 or 48 hours to collect any new flies that are trapped. For this part, the researchers will not require your help other than your permission to work around their house. As they leave and collect the trap, the researchers will take some measurements next to the trap, including the temperature. When collecting the trap, they might ask you if it rained at your house overnight. It is very important that the trap is not disturbed over the 24 hours that it is left at your house, because that might affect the research.

☐ <u>**Durability household (repellent headwear only)**</u>

We will visit your house once, at a time that is suitable for you. This visit will take no more than 30 minutes, we will contact you in advance to organise a convenient time with you. It will be necessary that your children aged 2 to 9 years are at home while we visit.

During the visit, you and your child will need to be available at your house. The researchers will ask your child to sit on a chair, wearing their repellent headwear (scarf/cap). The researchers will then observe the flies on your child's face. They will also video and photograph their face so that we can analyse fly behaviour later. They will record the weight and body temperature of your child.

Finally, the researchers will take away the repellent headwear, but they will give the child a new one. This is because we want to conduct further tests on the repellent headwear.

Why have I been selected for this study?

You have been invited because in the area that you live there is trachoma, and there are many flies. Your household has been chosen for frequent visits because there are children of the right age living in your house.

We will discuss the study together and give you a copy of this information sheet. If you agree to take part, we will then ask you to sign a consent form.

What will I have to do?

It is important that your 2 to 9 year-old child is at home for our visits. For the rest of the household, other than allowing us to place a trap (or traps) next to your house, and visit your house to leave and collect the traps, you do not need to do anything.

Do I have to take part?

No. It is up to you to decide to take part or now. If you don't want to take part, that's ok. You can withdraw from any part of the study at any time, for any reason. If you do agree, you are still free to withdraw the participation of your household at any time.

If your child chooses to take part, it is VOLUNTARY

Your child is free to choose whether they want to take part or not, and their primary caregiver is also free to choose if they allow their child to take part or not. If your child agrees, they are still free to withdraw from the study at any time. If you don't want your child to take part, that's ok.

What are the possible risks and disadvantages?

The risks and disadvantages remain the same as participating in the Stronger-SAFE study. These are copied again below for you to check. The main disadvantage is that our research team will visit your household more often.

"If your house receives fly control measures, there is a small risk that your child will experience skin irritation or a reaction during or after wearing the repellent headwear. If this happens, please immediately take it from them, wash the area with soap and water, and contact someone in our team. If this happens to your child, they will be withdrawn from the study. Because the repellent headwear has a chemical in it, it is best that these items don't contact the eyes, nose or mouth of the child, as this may not be good for them. However, even if this happens, it is unlikely that it will lead to problems. Flies caught in the fly traps present a small risk to people, as flies are unhygienic. Therefore, only those who have been trained in using the trap should touch it. The lure inside the trap does not contain bad chemicals, it is composed of food-based substances including yeast. For these hygiene reasons, it is important that children are discouraged from touching the trap and specifically the lure (bait) or the flies trapped inside."

What are the possible benefits?
We cannot promise the study will help you, but the information we get from the study will help our knowledge and understanding of trachoma. It is possible that the traps may reduce the numbers of flies around your house, and it is possible that while your child/children use the repellent headwear, they may experience less irritation and bother from fly contact to their face.

What if something goes wrong?
If you have a concern about any aspect of this study, you should ask to speak to the researchers who will do their best to answer your questions (Mr Oumer Shafi Abdulrahman, Tel: +251 91 204 8181).

The London School of Hygiene and Tropical Medicine holds insurance policies which apply to this study. If any member of your household, including your child, experiences harm or injury as a result of taking part in this study, you may be eligible to claim compensation.

Can I change my mind about taking part?
Yes. You can withdraw from the study at any time. You just need to tell our researchers that you don't want to be in the study anymore. If your household, or your child, decides to withdraw from the study, we will need to use the data collected (fly trapping data and the video footage) up to the point of withdrawal.

What will happen to information collected about me?
All information collected about you, including videos/photos taken and geolocations, will be kept private. Only the study staff and authorities who check that the study is being carried out properly will be allowed to look at information about you. Data may be sent to other study staff in London or Ethiopia but this will be anonymised. The photographs/videos will be used for analysing fly behaviour, and they will be anonymised (not connected with your name or other personal identifiers). They might also be used as teaching material, or in presentations for other people, but they will not be used for anything that you have not consented for. Data collected in the study may be made publicly accessible. However, this will not contain any identifying information

Our research team will send some details about you, your child and the household to the study teams in Shashemene and London who will store it securely. Your personal details will be kept in a different safe place to the other study information and will be destroyed within 10 years of the end of the study.

At the end of the project, the study data will be archived at the London School of Hygiene & Tropical Medicine. The data will be made available to other researchers worldwide for research and to improve medical knowledge and care of people with trachoma. Your personal information, and that of your child or other members of the household, will not be included and there is no way that you can be identified.

What will happen to the results of this study?
The study results will be published in a medical or public health journal so that other people who want to control trachoma can learn from them. Your personal information will not be included in the study report and there is no way that you can be identified from it.

Who is organising and funding this study?
London School of Hygiene & Tropical Medicine is the sponsor for the research and they have full responsibility for the project including the collection, storage and analysis of your data.

Who has checked this study?
All research involving human participants is looked at by an independent group of people, called a Research Ethics Committee, to protect your interests. This study has been reviewed and given favourable opinion by The London School of Hygiene and Tropical Medicine Research Ethics Committee. The National and Oromia

Stronger SAFE
Eliminating Trachoma

Health Bureau Ethical Review Board, the Ministry of Science and Higher Education (MoSH)and the Ethiopian Food, Medicines and Health care administration and Control Authority (EFMHACA) have also reviewed the study and have agreed that it is okay for us to ask people to take part.

Contact Information

If you have any questions please ask us:

- if you have any questions about this study or your part in it,
- if you feel you have had a research-related injury or an adverse effect from the surgery or the drug,
- if you have questions, concerns or complaints about the research

Mr Oumer Shafi Tel: +251 91 204 8181.
Prof. Matthew Burton at +44 20-7636-8636 or matthew.burton@lshtm.ac.uk

NRERC: NRERC Secretariat
Tel: +251118720943
Email: nrerc2019@gmail.com

EFDA: Medicine Registration and License Directorate
Tel: 00251-1524122/524123
Email: efmhacapharmacovigilance@gmail.com
Email: regulatory@fmhaca.gov.et

You will be given a copy of the information sheet and a signed consent form to keep.
Thank you for considering taking the time to read this sheet.

APPENDIX 4D: CONSENT FORM – ENTOMOLOGY IN SENTINAL HOUSEHOLDS - HEAD

Federal Ministry of Health, Ethiopia
Fred Hollows Foundation, Ethiopia
Oromia Regional Health Bureau, Ethiopia
London School of Hygiene and Tropical Medicine, UK

Stronger SAFE: Phase 3 – Cluster-randomised trial of double-dose oral azithromycin combined with targeted transmission-interrupting strategies for trachoma elimination in Ethiopia

<u>CONSENT FORM – ENTOMOLOGY IN SENTINAL HOUSEHOLDS - HEAD</u>

HOUSEHOLD HEAD CONSENT FOR TRAPPING STUDIES (SENTINEL HOUSEHOLDS ONLY)

Household consent to participate in the sentinel fly trapping studies, by allowing fly traps to be placed outside and next to the house. The household head should complete this form if they wish to provide consent for the whole household to participate.

**Statement**	**Please initial or thumbprint* each box**
I have had the information explained to by study personnel in a language that I understand. I have had the opportunity to consider the information, ask questions and have these answered satisfactorily.	
I understand that the participation of this household in sentinel fly trapping studies is voluntary and that I am free to withdraw this household at any time without giving any reason.	
I understand that in participating in this study, the study team will place a fly trap outside my house but inside the compound for a short period of time (1-2 days). I understand that the team will visit to set up the trap, then remove it and any flies it has caught, every 6-8 weeks and for three years.	
I give permission for the researchers to make brief visits during those 6-8 weeks without prior notice, or to call the household phone, and I consent to participate in an interview with the researchers about this trap once every year of the study.	
I understand that data or photos about the householders may be shared via a public data repository or by sharing directly with other researchers, and that I/we will not be identifiable from this information	
I give permission for the research findings from this trapping work to be reported anonymously to communicate the findings of this research, to analyse this research and for teaching purposes. Information about the study could potentially be seen by researchers and students in the UK and beyond, and by health professionals and decision-makers in Ethiopia/UK and beyond.	
I agree for this household to take part in the above named study	

Stronger SAFE
Eliminating Trachoma

____

Signature / Thumbprint of Participant Name Date

____

Signature of Researcher Name Date

____

Signature of Impartial Witness* Name Date

***Note to researcher: Witness signature and date are required on this consent form only when the consenting volunteer is not able to read (illiterate). The researcher may be able to sign their name but still require a witness.**

APPENDIX 4E: INFORMATION SHEET – ENTOMOLOGY IN SENTINAL HOUSEHOLDS - CHILD

Federal Ministry of Health, Ethiopia
Fred Hollows Foundation, Ethiopia
Oromia Regional Health Bureau, Ethiopia
London School of Hygiene and Tropical Medicine, UK

Stronger SAFE: Phase 3 – Cluster-randomised trial of double-dose oral azithromycin combined with targeted transmission-interrupting strategies for trachoma elimination in Ethiopia

INFORMATION SHEET – ENTOMOLOGY IN SENTINAL HOUSEHOLDS - CHILD

Introduction
We would like to invite you to take part in a research study. Joining the study is entirely up to you. Before you decide, you need to understand why the research is being done and what it would involve. One of our team will go through this information sheet with you, and answer any questions you may have. Ask questions if anything you read is not clear or you would like more information. Please feel free to talk to others about the study if you wish. Take time to decide whether or not to take part.

What is the purpose of the study?
Your household is already part of the "Stronger-SAFE" project. Because of that, we need to make some measurements of the flies around your house.

What will happen to me if I take part?

☐ **Sentinel households (repellent headwear and trap)**
We need to take measurements regularly, so we will need to visit your house every six to eight weeks over the next three years. This visit will take no more than 30 minutes. We will contact you in advance and check when is the best time for you.

On that day, you and your caregiver will need to be available at your house. The researchers will ask you to sit on a chair, so that they can observe the flies on your face for ten minutes, and we will also video and photograph your face. If you were given a scarf or cap as part of the Stronger-SAFE project, we will ask you to wear it at that time. We will also record how much you weigh, and your ear temperature.

☐ **Durability household (repellent headwear only)**
We will visit your house once. Our visit will take no more than 30 minutes, and we will contact you in advance and check when is the best time for you.

On that day, you and your caregiver will need to be available at your house. The researchers will ask you to sit on a chair, so that they can observe the flies on your face for ten minutes, and we will also video and photograph your face. We will ask you to wear the scarf or cap you were given at that time. We will also record how much you weigh, and your ear temperature.

Why have I been selected for this study?
You have been chosen because you live somewhere where there are many flies, and you are the right age for this study. Flies prefer to go to the face of young children.

We will discuss the study together and give you a copy of this information sheet. If you agree to take part, we will ask your caregiver to sign a consent form for you.

What will I have to do?
We will need you to be available one day every six to eight weeks for three years. If you have been given a scarf or cap for Stronger-SAFE, we will ask you to continue wearing this when you wish to protect yourself from flies in-between those time points, just as you were already told.

Do I have to take part?
No. It is up to you to decide to take part or now. If you don't want to take part, that's ok. You can withdraw from our study at any time. Your primary caregiver is also free to choose whether you take part or not.

Will being in these studies be bad for me?
The main disadvantage for being in this study is that we will visit your house regularly over the next three years, and we will need to see you on that day. Otherwise, it is the same as being in the Stronger-SAFE project.

Will being in these studies be good for me or help me?
We want to invent new ways to stop people from catching trachoma. By taking part in this study, you will help us to do that.
What if something goes wrong?
If you have any worries about any aspect of this study, please speak to the researchers or your caregiver.

Can I change my mind about taking part?
Yes. You can withdraw from the study at any time. You just need to tell our researchers that you don't want to be in the study anymore.

What will happen to information collected about me?
All the information that we collect, and videos/photos taken, will be kept confidential (we will not share it with many people, only the study researchers). It will be kept safe. The photographs/videos will be used for measuring fly behavior on and around your face, and also may be used for teaching people or to show to other researchers, but they will only be used for things that we have asked your permission to use them for. In time, the data will be made available to other researchers and people, but they will not know that the data came from you.

Your personal details will be kept in a different safe place to the other study information and will be destroyed within 10 years of the end of the study. At the end of the project, the study data will be archived at LSHTM. The data will be made available to other researchers worldwide for research and to improve medical knowledge and patient care. Your personal information will not be included.

Who has checked this study?
Several committees, both in the UK and in Ethiopia, are working to protect your rights and welfare in this project.

<u>Contact Information</u>
If you have any questions please ask us:
- if you have any questions about this study or your part in it,
- if you feel you have had a research-related injury or an adverse effect from the surgery or the drug,
- if you have questions, concerns or complaints about the research

Mr Oumer Shafi Tel: +251 91 204 8181.

Prof. Matthew Burton at +44 20-7636-8636 or matthew.burton@lshtm.ac.uk

NRERC: NRERC Secretariat
Tel: +251118720943
Email: nrerc2019@gmail.com

EFDA: Medicine Registration and License Directorate
Tel: 00251-1524122/524123
Email: efmhacapharmacovigilance@gmail.com
Email: regulatory@fmhaca.gov.et

You will be given a copy of the information sheet and a signed consent form to keep.
Thank you for considering taking the time to read this sheet.

APPENDIX 4F: CONSENT FORM – ENTOMOLOGY IN SENTINAL HOUSEHOLDS - CHILD

Federal Ministry of Health, Ethiopia
Fred Hollows Foundation, Ethiopia
Oromia Regional Health Bureau, Ethiopia
London School of Hygiene and Tropical Medicine, UK

Stronger SAFE: Phase 3 – Cluster-randomised trial of double-dose oral azithromycin combined with targeted transmission-interrupting strategies for trachoma elimination in Ethiopia

CONSENT FORM – ENTOMOLOGY IN SENTINAL HOUSEHOLDS - CHILD

PRIMARY CAREGIVER CONSENT FOR FLY-EYE STUDIES (SENTINEL/DURABILITY HOUSEHOLDS)

Consent from the primary caregiver for their child to participant in fly-eye studies. Consent should be obtained for one child aged between 2 and 9 years.

**Statement**	**Please initial or thumbprint* each box**
I have had the information explained to by study personnel in a language that I understand. I have had the opportunity to consider the information, ask questions and have these answered satisfactorily.	
I understand that the participation of my child is voluntary and that they are free to withdraw at any time without giving any reason, and that I am free to withdraw my child at any time without giving any reason.	
I understand that in participating in this study, my child will have their face observed, filmed and photographed.	
**Sentinel household:** I understand that this will be repeated every 6-8 weeks, for three years. I give permission for the researchers to make brief visits during those 6-8 weeks without prior notice, or to call the household phone, and I consent to participate in an interview with the researchers and my child about this repellent headwear once every year of the study.	
**Durability household:** I understand that this will be one visit.	
I give permission for videos and photos to be taken of my child.	
I understand that data/videos/photos collected from my child may be shared via a public data repository or by sharing directly with other researchers, and that I/we will not be identifiable from this information	
I give permission for the research findings about my child to be reported anonymously to communicate the findings of this research, to analyse this research and for teaching purposes. Information about the study could potentially be seen by researchers and students in the UK and beyond, and by health professionals and decision-makers in Ethiopia/UK and beyond.	

I give my consent for my child who is under the age of 18 years and for whom I am the parent or guardian to take part in the above named study.	

**Statement**	**Please initial or thumbprint* each box**
I give permission for ANONYMISED photos/videos to be used in the following ways:	
As part of the study report	
For teaching purposes, or in presentations for other researchers or the public	
In other reports, campaigns and publications by LSHTM or affiliated partners and donors	
On the LSHTM website or in other media about this study	
As part of a public data repository	

____

Signature / Thumbprint of Participant Name Date

____

Signature of Researcher Name Date

____

Signature of Impartial Witness* Name Date

***Note to researcher: Witness signature and date are required on this consent form only when the consenting volunteer is not able to read (illiterate). The researcher may be able to sign their name but still require a witness.**

APPENDIX 4G: INFORMATION SHEET – WASH OUTCOMES

Federal Ministry of Health, Ethiopia
Fred Hollows Foundation, Ethiopia
Oromia Regional Health Bureau, Ethiopia
London School of Hygiene and Tropical Medicine, UK

Stronger SAFE: Phase 3 – Cluster-randomised trial of double-dose oral azithromycin combined with targeted transmission-interrupting strategies for trachoma elimination in Ethiopia

INFORMATION SHEET – WASH OUTCOMES

Introduction
We would like to invite you to take part in a research study. Joining the study is entirely up to you. Before you decide, you need to understand why the research is being done and what it would involve. One of our team will go through this information sheet with you, and answer any questions you may have. Ask questions if anything is not clear or you would like more information. Please feel free to talk to others about the study if you wish. Take time to decide whether to take part.

What is the purpose of this study?
Trachoma is an eye disease that many people catch in Ethiopia. It can cause people to go blind. Trachoma is transmitted from eye to eye in a number of different ways. We believe we can help prevent trachoma spreading through health programmes. In order to learn whether new programmes work, we need to understand more about daily life and behaviour in this area. The information we get from this study will help to improve future programmes to control trachoma in Oromia and elsewhere in Ethiopia.

What will I be asked to do if I choose to take part?
If you agree to participate, our researcher will arrange a convenient day and time to visit you at home to conduct the following activities. *Researcher to tick all relevant activities and read them to this participant.*

☐ **Structured observation:** On the agreed day of the research, our researcher will visit your home early in the morning OR in the middle of the day to observe your daily routines and the way you do things as a family for 3 hours. The researcher will not judge you or your family in any way; they are just interested in recording the activities you do in your home, so you should continue with your day as normal. They will not accompany you on errands away from the home.

☐ **Survey interview:** Our researcher will ask you some questions about yourself, your family and your domestic activities and childcare and they will record your answers on a form. They may also ask you about some health promotion activities and materials that you may have seen. They will ask to look around your home to help them answer some of these questions. One of your pre-school children may be asked to demonstrate an everyday behaviour during this interview. The researcher may video record this demonstration.

☐ **Facial assessment:** Our researcher will observe the face of the primary caregiver and all children in the home and take a photograph. They will then wipe each face with a cloth wipe and will take a photograph of this wipe. We will look at the amount of dirt or dust that comes off on to the wipe, and take a photograph of the wipe. This is not uncomfortable and will not cause any pain.

General information

Why have I been selected for this study?

You have been invited to participate in this study at random because the area you live in has trachoma. You also have young children, and it is children who are most commonly infected with trachoma.

How long is this study and how often will I need to take part?
The study will take approximately 4 hours to complete in total if you participate in all the activities, but may take as little as 1 hour if the observation is not conducted. Your household might be randomly selected to participate in more than one round of data collection over the next 3 years. However, choosing to participate now does not mean that you would have to participate again in the future.

Where is this study taking place?
This research is taking place in communities in Oromia. Around 260 households will participate in this study (including the observation).

Do I have to take part?
No! Your participation is voluntary. Our researcher will help you understand this form and answer your questions. It is up to you to decide to take part or not. If you do not want to take part, that is ok. You can withdraw from any part of the study at any time, for any reason. If you do agree, you are still free to withdraw yourself and your family at any time without any consequences to you or your family. Should you withdraw from the study, you will continue to obtain the regular benefits of any health care services you normally get at the clinic. Participating now does not mean you need to participate again in the future.

What are the possible benefits?
Research is designed to benefit the wider community by contributing new knowledge that will help shape future health programs. You might not benefit directly from the study.

What could go wrong?
This study does not pose any risks to you or your family. Our researchers are trained to respect your emotions and they will not comment on or judge you or your family's behaviour. If you feel uncomfortable with the researcher being in your house or carrying out any of the activities you should inform them immediately or ask to speak to their supervisor, who will do his best to answer your questions (Mr Oumer Shafi, Tel: +251912048181). The London School of Hygiene and Tropical Medicine holds insurance policies that apply to this study. If you experience harm or injury because of taking part in this study, you may be eligible to claim compensation.

What will happen to the information you collect?
All information collected about you, including videos/photos taken will be kept private and secure on password-protected computers or in locked cabinets. Only the people organising the study will have access to it. Reports and presentations summarizing the information collected in the study might be made publically accessible. However, we will not include your name or any personal details that could identify you as a participant in any information we publish about the study. The photographs of faces and face swabs will mainly be used for independent verification, and the people verifying will not know your name. If you give us permission, the face swabs, photographs of these swabs and photographs of your family's faces will be used to support other research in the future, and may be shared anonymously with other researchers, for their ethically approved projects.

What if I still have questions about this study or my rights as a participant?
All research on human volunteers is reviewed by both the National and Oromia Health Bureau Ethical Review Board and LSHTM Research Ethics Committee that works to protect your rights and welfare. You have the right to ask, and have answered, any questions you may have about this research and your participation. If you have questions, complaints, or concerns please contact Mr Oumer Shafi.

Who is carrying out this study?

This study is being conducted through a partnership between the Ethiopian Federal Ministry of Health, the Oromia Regional Health Bureau, the Fred Hollows Foundation Ethiopia and the London School of Hygiene & Tropical Medicine, UL. The London School of Hygiene & Tropical Medicine will act as the trial sponsor. The trial is funded by the Wellcome Trust (UK).

<u>**Contact Information**</u>

If you have any questions please ask us:

- if you have any questions about this study or your part in it,
- if you feel you have had a research-related injury or an adverse effect from the surgery or the drug,
- if you have questions, concerns or complaints about the research

Mr Oumer Shafi Tel: +251 91 204 8181.
Prof. Matthew Burton at +44 20-7636-8636 or matthew.burton@lshtm.ac.uk

NRERC: NRERC Secretariat
Tel: +251118720943
Email: nrerc2019@gmail.com

EFDA: Medicine Registration and License Directorate
Tel: 00251-1524122/524123
Email: efmhacapharmacovigilance@gmail.com
Email: regulatory@fmhaca.gov.et

You will be given a copy of the information sheet and a signed consent form to keep.
Thank you for considering taking the time to read this sheet.

APPENDIX 4H: CONSENT FORM – WASH OUTCOMES

Federal Ministry of Health, Ethiopia
Fred Hollows Foundation, Ethiopia
Oromia Regional Health Bureau, Ethiopia
London School of Hygiene and Tropical Medicine, UK

Stronger SAFE: Phase 3 – Cluster-randomised trial of double-dose oral azithromycin combined with targeted transmission-interrupting strategies for trachoma elimination in Ethiopia

CONSENT FORM – WASH OUTCOMES

TO BE COMPLETED BY THE PRIMARY CAREGIVER IN ALL STUDY HOUSEHOLDS

Stronger SAFE: Phase 3 – Cluster-randomised trial of double-dose oral azithromycin combined with targeted transmission-interrupting strategies for trachoma elimination in Ethiopia

**Statement**	**Please initial or thumbprint* each box**
I have read/been read the information provided above and I have understood it. I have asked all the questions I have at this time.	
I understand the activities that are to take place, and I give permission for researchers to visit my household on up to five other occasions over the next 3 years	
I understand that it is my right to withdraw from the study at any time without giving any reason, without my medical care or legal rights being affected.	
I understand that data collected during the study may be looked at by authorised individuals from the London School of Hygiene and Tropical Medicine, Fred Hollows Foundation. I give permission for these individuals to have access to my records.	
I understand that data and photos may be shared via a public data repository or by sharing directly with other researchers, and that I will not be identifiable from this information.	
I give permission for my data to be reported anonymously to communicate the findings of this research, to analyse this research and for teaching purposes. I understand that samples and information collected in this study could potentially be seen by researchers and students in the UK and beyond, and by health professionals and decision-makers in Ethiopia/UK and beyond.	
I give my consent for all household members under 18 for whom I am the parent or guardian to participate in the study.	

Photos and videos of me and my children may be taken to document the research (circle one): **Yes** **No**

**I give permission for photos and videos from my family to be used as follows:**	**YES** ✔	**NO** ✘
As part of this study report		
In other reports, campaigns and publications by LSHTM or affiliated partners and donors		
On the LSHTM website or in other media about this study		

As part of a public data repository		

____

Signature / Thumbprint of Participant Name Date

____

Signature of Researcher Name Date

____

Signature of Impartial Witness* Name Date

***Note to researcher: Witness signature and date are required on this consent form only when the consenting volunteer is not able to read (illiterate). The researcher may be able to sign their name but still require a witness.**

Stronger SAFE
Eliminating Trachoma

APPENDIX 4I: CONSENT FORM – WASH OUTCOMES

Federal Ministry of Health, Ethiopia
Fred Hollows Foundation, Ethiopia
Oromia Regional Health Bureau, Ethiopia
London School of Hygiene and Tropical Medicine, UK

Stronger SAFE: Phase 3 – Cluster-randomised trial of double-dose oral azithromycin combined with targeted transmission-interrupting strategies for trachoma elimination in Ethiopia

<u>CONSENT FORM – WASH OUTCOMES</u>

TO BE COMPLETED BY ANY OTHER ADULTS (18 AND OVER) IN HOUSEHOLDS THAT ARE TO BE OBSERVED

**Statement**	**Please initial or thumbprint* each box**
I have read/been read the information provided above and I have understood it. I have asked all the questions I have at this time.	
I understand the activities that are to take place, and I give permission for researchers to visit my household on up to five other occasions over the next 3 years	
I understand that it is my right to withdraw from the study at any time without giving any reason, without my medical care or legal rights being affected.	
I understand that data collected during the study may be looked at by authorised individuals from the London School of Hygiene and Tropical Medicine, Fred Hollows Foundation. I give permission for these individuals to have access to my records.	
I understand that data and photos may be shared via a public data repository or by sharing directly with other researchers, and that I will not be identifiable from this information.	
I give permission for my data to be reported anonymously to communicate the findings of this research, to analyse this research and for teaching purposes. I understand that samples and information collected in this study could potentially be seen by researchers and students in the UK and beyond, and by health professionals and decision-makers in Ethiopia/UK and beyond.	

____
Signature / Thumbprint of Participant Name Date

____
Signature of Researcher Name Date

____
Signature of Impartial Witness* Name Date

***Note to researcher: Witness signature and date are required on this consent form only when the consenting volunteer is not able to read (illiterate). The researcher may be able to sign their name but still require a witness.**

APPENDIX 4J: ASSENT FORM – WASH OUTCOMES

Federal Ministry of Health, Ethiopia
Fred Hollows Foundation, Ethiopia
Oromia Regional Health Bureau, Ethiopia
London School of Hygiene and Tropical Medicine, UK

Stronger SAFE: Phase 3 – Cluster-randomised trial of double-dose oral azithromycin combined with targeted transmission-interrupting strategies for trachoma elimination in Ethiopia

ASSENT FORM – WASH OUTCOMES

TO BE COMPLETED BY INDIVIDUALS AGED 10-17 YEARS IN HOUSEHOLDS TO BE OBSERVED

Your parents / guardians have agreed for your household to part of a research study, but I also need to ask you if you are happy to take part. The study is to learn more about the daily routines and practices of rural Ethiopians and how this may affect health and the transmission of disease.

If you agree to take part then the researchers who will be in your home will take notes about what you are doing during the time they are in your home. They may ask you some questions about your daily activities. Your name will not be recorded and nobody will know that your family took part in the study. They may ask to take a photo of your face and use a wipe on your face. You don't have to take part in the study if you don't want to even though the researchers will be in your home. You can ask me any questions about the study now. If you are willing to take part I need to ask you to write your name or make your thumb print on this sheet to show you have given your permission.

I, ______________________________ have read/been read the information provided above and I have understood it. I have asked all the questions I have at this time. I understand that it is my right to withdraw from the study at any time without it affecting me or my family. I understand that the information/photos/swab sample collected from me will be used to support other research in the future, and may be shared anonymously with other researchers, for their ethically-approved projects.

I am willing to take part in this study (tick one box). ☐ **Yes** ☐ **No**

I agree to photos of me to be taken to document the research (tick one box). ☐ **Yes** ☐ **No**

I agree to a face wipe to be taken from my face (tick one box). ☐ **Yes** ☐ **No**

**I give permission for photos of me (not named) and anonymised results to be used in the following ways:**	**YES** ☑	**NO** ☒
As part of this study report		
In other reports, campaigns and publications by LSHTM or affiliated partners and donors		
On the LSHTM website or in other media about this study		
As part of a public data repository		

Stronger SAFE
Eliminating Trachoma

____

Signature / Thumbprint of Participant Name Date

____

Signature of Researcher Name Date

____

Signature of Impartial Witness* Name Date

***Note to researcher: Witness signature and date are required on this consent form only when the consenting volunteer is not able to read (illiterate). The researcher may be able to sign their name but still require a witness.**

Stronger SAFE
Eliminating Trachoma

APPENDIX 4K: INFORMATION SHEET – F&E MONITORING

Federal Ministry of Health, Ethiopia
Fred Hollows Foundation, Ethiopia
Oromia Regional Health Bureau, Ethiopia
London School of Hygiene and Tropical Medicine, UK

Stronger SAFE: Phase 3 – Cluster-randomised trial of double-dose oral azithromycin combined with targeted transmission-interrupting strategies for trachoma elimination in Ethiopia

INFORMATION SHEET – F & E MONITORING

What is the purpose of this study?
Trachoma is an eye disease that many people catch in Ethiopia. It can cause people to go blind. Trachoma is transmitted from eye to eye in a number of different ways. We believe we can help prevent trachoma spreading through health programmes. We would like to learn more about trachoma programmes in Oromia. The information we get from this study will help to improve future programmes to control trachoma in Oromia and elsewhere in Ethiopia.

What will I be asked to do if I participate in these studies?
Researcher to tick options applicable to this participant:

☐ **Implementer Interview:** Should you agree to participate, our researchers will ask you some questions about your experiences implementing the Stronger-SAFE intervention. During this process they may use props and images to explore different ideas with you. There is no right or wrong answer to anything they ask and we would encourage you to be open and honest in your responses so that we can learn. These discussions will be audio recorded. The recordings will only be listened to by the researchers undertaking this study and will be used to anonymously capture things that you have said without reference to your name, your community or your work. The interview will take place in a private location that is convenient for you. The discussion may take up to an hour.

☐ **Key Informant Interview:** Should you agree to participate, our researchers will ask you some questions about trachoma and its control, including some recent health programmes in this area. During this process they may use props and images to explore different ideas with you. There is no right or wrong answer to anything they ask and we would encourage you to be open and honest in your responses so that we can learn. These discussions will be audio recorded. The recordings will only be listened to by the researchers undertaking this study and will be used to anonymously capture things that you have said without reference to your name, your community or your work. The interview will take place in a private location that is convenient for you. The discussion may take up to an hour.

☐ **Participant Exit Interview:** Should you agree to participate, our researchers will ask you some questions about the activity you have just participated in. During this process they may use props and images to explore different ideas with you. There is no right or wrong answer to anything they ask and we would encourage you to be open and honest in your responses so that we can learn. These discussions will be audio recorded. The recordings will only be listened to by the researchers undertaking this study and will be used to anonymously capture things that you have said without reference to your name, your community or your work. The interview will take place in a private location that is convenient for you. The discussion will take no more than 30 minutes of your time.

☐ **School or health centre audit and interview:** Should you agree to participate, our researcher will ask to be shown around the school or health centre/post and they will ask you some questions about the community and everyday activities that take place in this facility. The discussion will be audio recorded. The audio recordings will only be listened to by the researchers undertaking this study and will be used to anonymously capture things that you have said without reference to your name or school. The discussion should take no more than 30 minutes of your time.

General Information

Where are these studies taking place?
This research is taking place in communities in Oromia. Up to 50 individuals may be interviewed in total.

Do I have to take part?
No! Your participation is voluntary. Our researcher will help you understand this form and answer your questions. It is up to you to decide to take part or not. If you don't want to take part, that's ok. You can withdraw from any part of the study at any time, for any reason. If you do agree, you are still free to withdraw yourself and your family at any time without any consequences to you or your family. Should you withdraw from the study, you will continue to obtain the regular benefits of any health care services you normally get at the clinic.

What are the possible benefits?
Research is designed to benefit the wider community by contributing new knowledge which will help shape future health programs. You may, however, receive no direct benefit from the study.

What could go wrong?
This study does not pose any risks to you or your family. Our researchers are trained to respect your emotions and they will not comment on or judge you. If you feel uncomfortable with the researcher asking any questions you should inform them immediately or ask to speak to their supervisor, who will do his best to answer your questions (Mr Oumer Shafi, Tel: +251912048181). The London School of Hygiene and Tropical Medicine holds insurance policies which apply to this study. If you experience harm or injury as a result of taking part in this study, you may be eligible to claim compensation.

What will happen to the information you collect?
All information collected about you, including audio recordings, will be kept private and secure on password-protected computers or in locked cabinets. Only the people organising the study will have access to it. Reports and presentations summarizing the information collected in the study may be made publically accessible. However, we will not include your name or any personal details that could identify you as a participant in any information we publish about the study. If you give us permission, the information we collect may be used to support other research in the future, and may be shared anonymously with other researchers, for their ethically-approved projects.

What if I still have questions about this study or my rights as a participant?
All research on human volunteers is reviewed by both the National and Oromia Health Bureau Ethical Review Board and LSHTM Research Ethics Committee that works to protect your rights and welfare. You have the right to ask, and have answered, any questions you may have about this research and your participation. If you have questions, complaints, or concerns please contact Mr Oumer Shafi.

Who is carrying out this study?
This study is being conducted through a partnership between the Ethiopian Federal Ministry of Health, the Oromia Regional Health Bureau, the Fred Hollows Foundation Ethiopia and the London School of Hygiene &

Tropical Medicine, UL. The London School of Hygiene & Tropical Medicine will act as the trial sponsor. The trial is funded by the Wellcome Trust (UK).

<u>Contact Information</u>
If you have any questions please ask us:

- if you have any questions about this study or your part in it,
- if you feel you have had a research-related injury or an adverse effect from the surgery or the drug,
- if you have questions, concerns or complaints about the research

Mr Oumer Shafi Tel: +251 91 204 8181.
Prof. Matthew Burton at +44 20-7636-8636 or matthew.burton@lshtm.ac.uk

NRERC: NRERC Secretariat
Tel: +251118720943
Email: nrerc2019@gmail.com

EFDA: Medicine Registration and License Directorate
Tel: 00251-1524122/524123
Email: efmhacapharmacovigilance@gmail.com
Email: regulatory@fmhaca.gov.et

You will be given a copy of the information sheet and a signed consent form to keep.
Thank you for considering taking the time to read this sheet.

APPENDIX 4L: CONSENT FORM – F&E MONITORING

Federal Ministry of Health, Ethiopia
Fred Hollows Foundation, Ethiopia
Oromia Regional Health Bureau, Ethiopia
London School of Hygiene and Tropical Medicine, UK

Stronger SAFE: Phase 3 – Cluster-randomised trial of double-dose oral azithromycin combined with targeted transmission-interrupting strategies for trachoma elimination in Ethiopia

CONSENT FORM – F & E MONITORING

**Statement**	**Please initial or thumbprint* each box**
I have read/been read the information provided above and I have understood it. I have asked all the questions I have at this time.	
I understand that it is my right to withdraw from the study at any time without giving any reason, without my medical care or legal rights being affected.	
I understand that data collected during the study may be looked at by authorised individuals from the London School of Hygiene and Tropical Medicine, Fred Hollows Foundation. I give permission for these individuals to have access to my records.	
I understand that data, including audio recording from interviews may be shared via a public data repository or by sharing directly with other researchers, and that I will not be identifiable from this information.	
I give permission for the research findings from this work to be reported anonymously to communicate the findings of this research, to analyse this research and for teaching purposes. I understand that samples and information collected in this study could potentially be seen by researchers and students in the UK and beyond, and by health professionals and decision-makers in Ethiopia/UK and beyond.	

____

Signature / Thumbprint of Participant Name Date

____

Signature of Researcher Name Date

____

Signature of Impartial Witness* Name Date

***Note to researcher: Witness signature and date are required on this consent form only when the consenting volunteer is not able to read (illiterate). The researcher may be able to sign their name but still require a witness.**
