# Supplementary material for: Cluster randomised controlled trial of double-dose azithromycin mass drug administration, facial cleanliness and fly control measures for trachoma control in Oromia, Ethiopia: the stronger SAFE trial protocol
Source: BMJ Open. 2024 Dec 23;14(12):e084478. doi: 10.1136/bmjopen-2024-084478 (PMC11751794; doi:10.1136/bmjopen-2024-084478)
Supplement: Supplementary file 14 [file bmjopen-2024-084478.reviewer_comments.pdf]

PEER REVIEW HISTORY

BMJ Open publishes all reviews undertaken for accepted manuscripts. Reviewers are asked to complete a checklist review form and are provided with free text boxes to elaborate on their assessment. These free text comments are reproduced below.

ARTICLE DETAILS

Title (Provisional)

Cluster randomised controlled trial of double-dose azithromycin mass drug administration, facial cleanliness and fly control measures for trachoma control in Oromia, Ethiopia: The Stronger SAFE trial protocol

Authors

Last, Anna R.; Shafi Abdurahman, Oumer; Greenland, Katie; Robinson, Ailie; Collin, Claire; Czerniewska, Alexandra; Etu, Edao Sinba; Versteeg, Bart; Butcher, Robert; Guye, Meseret; Legesse, Demitu; Nuri, Kedir Temam; Shuka, Gemeda; Haji Mohammed Yousuf, Munira; Dheressa, Gaddisa; Dumessa, Gebeyehu; Akalu, Melesse; Tadesse, Mesfin; Adugna Kumsa, Dereje; Seife Gebretsadik, Fikre; Abashawl, Aida; Habtamu, Esmael; Sarah, Virginia; Alemayehu, Wondu; Solomon, Anthony; Weiss, Helen Anne; Logan, James G.; Macleod, David; Burton, Matthew J

VERSION 1 - REVIEW

**Reviewer**	**1**
**Name**	**O'Brien, Kieran**
**Affiliation**	**University of California, San Francisco, Francis I. Proctor Foundation**
**Date**	**13-Apr-2024**
**COI**	**None**

This study protocol describes a 4-arm cluster-randomized trial comparing a combination of enhanced A, F, and E interventions to standard SAFE implementation to control trachoma in Ethiopia. The protocol is well written, clearly described, and presents a trial with the potential to impact the field and thus I recommend this manuscript for publication, with the minor revisions suggested below.

Introduction. The rationale for the trial is made well. The authors mention a modeling exercise that examined the efficacy of the proposed interventions – it would be great to add another sentence with more detail about that work and the results to help make the case for the trial. Related, I think the introduction could benefit from a bit more detail on the rationale for the specific azithromycin MDA approach taken, the double dose strategy and its

timing. Lines 11-12 call for strengthened antibiotic schedules and transmission reduction approaches – could the authors add another sentence elaborating on why this specific approach meets this call, including rationale for and evidence supporting the choice of two doses, and the two weeks in between?

• Line 5 – missing the word “of” (preventable infectious cause of blindness)

Methods

• Study setting, lines 37-39 on page 7 – would it be possible for the authors to provide a map of the study communities in a bit more detail, perhaps as an additional panel in Supplementary 2, in order to visualize the geographic spread and proximity of the ‘white’ and the ‘yolk’ households within a cluster and between clusters? I realize it might be challenging to see all households/clusters, so one option might be to show a few in a second map.

• Antibiotic Interventions, starting line 42 on page 7 – while I do understand the rationale for the order of methods sections presented, I found myself wondering why “two clusters per zone” was emphasized throughout the Interventions section, and so needed to scroll down to understand the randomization first. I imagine others will wonder as well – consider moving the Randomization section to appear before the Interventions section.

• Fly control interventions, starting line 25 on page 8 – did the team’s prior studies include evaluation of acceptability and/or likelihood of actual and proper use of either intervention? If so, this would be great to mention in this section as well.

• Fidelity of hygiene behavior change intervention, starting line 9 on page 10 – I might have missed it, are there any measures of whether the household is following the intervention? Actually, now I see it in the population for outcome monitoring section. Perhaps the authors could mention on page 10 that fidelity to the intervention is also monitored as an outcome and described later?

• Seasonal reinforcement events on page 10 – could the authors add 1-2 more sentences describing what these are and how they are implemented? Also, what are the enhanced efforts used to ensure socially excluded and vulnerable households are included?

• Census and baseline surveys on page 10– how was the sample size of 3 households per cluster decided for the WASH survey at baseline?

• Primary outcome, on page 11, in the paragraph about population for outcome monitoring, the authors indicate that “all children aged 1-9 years in the “yolks”… will be examined and sampled” and then on page 12 starting lines 7-8, the authors indicate that a random sample of 60 children will be taken at the primary outcome time point. Could the authors reword one or both of these sections to make them consistent? The way it currently reads, I thought the first section implied that every child would be monitored for outcomes (“examined and sampled” – not exactly sure whether sampling here refers to sampling to be included in the monitoring population or having samples taken). It sounds like repeated cross-sectional

random sampling is in fact used, presumably at all time points? Or is this only used at the primary time point and all children are taken at all other time points?

• Sample size, page 12 – could the authors provide references for the assumptions about Ct prevalence in the comparison arm and the expected ICC?

• Analysis – would the authors provide a reference and/or justification for their choice of approach to estimate risk difference? I'm curious about the rationale for the two-stage approach as opposed using a risk difference model in one step (such as a GLM with a binomial outcome and an identity link)? Now that I've read further, in the secondary outcomes section, the Hayes & Moulton reference is given – if this is referencing the same method, could the authors move this sentence up to the primary outcome section?

o Also, is this all estimated at the cluster level, or is this estimated at the individual level with some method used to account for clustering (and if so, what method)?

• Figure 2 – the image appears distorted or stretched for me

Reviewer 2

Name Xiong, Tao

Affiliation West China Second University Hospital, Sichuan University, Department of Pediatrics

Date 13-Jul-2024

COI none

This manuscript is a protocol for a cluster randomised controlled trial of enhanced antibiotic or/and enhanced F&E on trachoma control. This study could help in decision-making for trachoma programme managers. The method is detailed and the design is strict, but there are some concerns.

1. Based on the trial status provided in the manuscript, it seems that lots of works have been done. The authors stated "Further reinforcement events for the 'Enhanced F&E' interventions will be held in 2023-2024. The primary outcome survey will be conducted in May 2024, and thereafter data collection will end". Were these works complete as expected? If so, these works should state in the past tense. Importantly, was the data collection finished? If it was complete, the protocol will not suitable for publication.

2. Based on the manuscript, three annual rounds of antibiotic MDA were delivered. Assessments will be conducted at 2 month, 12 months, 14 months, 24 months, 26 months, and 36 months after the first baseline assessment. Was the first year of MDA conducted during the baseline assessment time? Moreover, it is not clear whether the second and third

year of annual MDA were delivered prior to or after the assessments at 12 months and 24 months.

3. Three assessments was conducted at 2 months, 14 months, and 26 months (2 months after annual MDA in standard antibiotic arms and 1.5 months after annual MDA in enhanced antibiotic arms). There is a concern about whether 2 months is too short to compare the effectiveness between standard and enhanced MDA. Given that the prevalence of ocular Ct could drop rapidly after antibiotic MDA, and it could rebound as time goes by, the effectiveness of enhanced antibiotic could be overestimated if the assessment times are too close to antibiotic distribution time.

4. This study is conducted in Oromia, Ethiopia. MDA with azithromycin has been given annually in this region since 2016. Prior to the trial intervention MDA had not been given since 2018. How about the baseline prevalence of ocular Ct or active trachoma in this region. How the previous MDA perform on controlling trachoma in this region? It is better to show some local epidemiologic data about the prevalence of trachoma in the introduction part.

5. Why children under two years of age are excluded for the second dose of azithromycin in the enhanced antibiotic arms instead of under six months of age.

6. Comparison will be conducted between the stronger SAFE arm (arm 4) and the standard SAFE arm (arm 1). Whether the comparisons between arm 2 and arm 1 or between arm 3 and 1 will be performed.

VERSION 1 - AUTHOR RESPONSE

We would like to extend our thanks to the reviewers for their consideration of our manuscript for the approved protocol for a cluster randomised controlled trial of double-dose azithromycin mass drug administration, facial cleanliness and fly control measures for trachoma control in Oromia, Ethiopia (Stronger SAFE). We hope that you will find our response, with requested amendments to our manuscript, sufficient to recommend publication within your journal for this globally important trial in trachoma control.

Reviewer: 1
Miss Kieran O'Brien, University of California, San Francisco
Comments to the Author:
This study protocol describes a 4-arm cluster-randomized trial comparing a combination of enhanced A, F, and E interventions to standard SAFE implementation to control trachoma in Ethiopia. The protocol is well written, clearly described, and presents a trial with the potential to impact the field and thus I recommend this manuscript for publication, with the minor revisions suggested below.

Thank you for your considered review of our protocol manuscript. We have provided our response to your queries below (in bold).

Introduction

The rationale for the trial is made well. The authors mention a modelling exercise that examined the efficacy of the proposed interventions – it would be great to add another sentence with more detail about that work and the results to help make the case for the trial. Related, I think the introduction could benefit from a bit more detail on the rationale for the specific azithromycin MDA approach taken, the double dose strategy and its timing. Lines 11-12 call for strengthened antibiotic schedules and transmission reduction approaches – could the authors add another sentence elaborating on why this specific approach meets this call, including rationale for and evidence supporting the choice of two doses, and the two weeks in between?

We regret that there isn't additional word space to include much more on this, but we have referenced previous studies relating to these decisions (Line 111), and have additionally added a referenced comment about the modelling study by Pinsent et al (BMC Medicine 2016) which informed the azithromycin dose and combination intervention strategies (Lines 120-123).

• Line 5 – missing the word "of" (preventable infectious cause of blindness)

Corrected (Line 105).

Methods

• Study setting, lines 37-39 on page 7 – would it be possible for the authors to provide a map of the study communities in a bit more detail, perhaps as an additional panel in Supplementary 2, in order to visualize the geographic spread and proximity of the 'white' and the 'yolk' households within a cluster and between clusters? I realize it might be challenging to see all households/clusters, so one option might be to show a few in a second map.

The map in Supplementary 2 shows the scale of the study area. We have updated the map and expanded the legend to include further information on cluster make-up. In brief, each cluster is comprised of 90 households (approximately three administrative 'garee' units). The 'yolk' includes 50 of these households, with the 'white' including the remaining 40 households. The 'white' provides a (small) buffer zone, deemed adequate given the nature of the household-level enhanced F&E interventions. The relative proximity of the clusters was due to logistic constraints (including significant security concerns in the study region during the study). The programmatic distribution of azithromycin MDA necessitated MDA to be given at 'zone' level, which also ensured a wide enough buffer zone and adequate separation between clusters randomised to receive single or double dose azithromycin.

The distribution of MDA at 'zone' level meant that randomisation was done so that two clusters per zone were allocated to one of the two MDA options. Please note that there is some heterogeneity in geographic spread due to clusters being defined by administrative (population-based) units (kebeles, zones and garees). We have taken care to ensure that clusters are separate enough to avoid significant contamination between arms, whilst ensuring the feasibility of the intervention delivery and outcome measurements.

• Antibiotic Interventions, starting line 42 on page 7 – while I do understand the rationale for the order of methods sections presented, I found myself wondering why "two clusters per zone" was emphasized throughout the Interventions section, and so needed to scroll down to understand the randomization first. I imagine others will wonder as well – consider moving the Randomization section to appear before the Interventions section.

We decided to order the sections in this way to ensure that the interventions were described early in the manuscript and to ensure that the randomization section was clear thereafter. We have added a signpost to the 'Randomization and Allocation of Clusters' (Line 179-80) for additional clarity. The rationale for this approach is detailed in Lines 308-311.

• Fly control interventions, starting line 25 on page 8 – did the team's prior studies include evaluation of acceptability and/or likelihood of actual and proper use of either intervention? If so, this would be great to mention in this section as well.

Thank you, yes, they did. This influenced the design and choice of interventions. This has been emphasised (Line 213). Proper use, adherence and acceptability of these interventions during the trial are also being monitored (Lines 417-418 and Supplementary 3).

• Fidelity of hygiene behavior change intervention, starting line 9 on page 10 – I might have missed it, are there any measures of whether the household is following the intervention? Actually, now I see it in the population for outcome monitoring section. Perhaps the authors could mention on page 10 that fidelity to the intervention is also monitored as an outcome and described later?

In the section detailing the 'Hygiene Behaviour Change Intervention', intervention fidelity is included (Lines 298-301) in addition to including this in the section describing the Population for Outcome Monitoring (Lines 416-419 and Supplementary 3).

• Seasonal reinforcement events on page 10 – could the authors add 1-2 more sentences describing what these are and how they are implemented? Also, what are the enhanced efforts used to ensure socially excluded and vulnerable households are included?

Seasonal reinforcement events were conducted in the dry and rainy seasons each year to reinforce messaging and allow for problem-solving of challenges faced by communities in adhering to the intervention, including overcoming specific seasonal barriers related to water availability and affordability of soap, and maintenance of wash stations. These events involved gender-separate discussions with groups of 10 households at a time. We have added further detail to this section (Lines 292-296). Regarding the inclusion of socially marginalised and vulnerable households, care was taken during the grouping of households to ensure any local dynamics did not limit participation (Line 301). Invited households that did not attend intervention activities were followed up at home to ensure that key elements of the intervention could be delivered, in line with our written protocol for delivery of the intervention through household follow-up.

• Census and baseline surveys on page 10– how was the sample size of 3 households per cluster decided for the WASH survey at baseline?

The full detail is included in Supplementary 3. This was not included in the main text due to journal word limits. In brief, the sample size calculation was based on the proportion of face washes that include soap during a three-hour observation period, as this was felt to be the behavioural outcome requiring the largest sample size. Assuming a prevalence of 25% in the control arm and a design effect of 1.33, a sample of 204 individuals per arm will provide 90% power to detect an increase in the prevalence to 40%. For practical reasons we opted to sample all individuals in three households in the 34 clusters in each of the four arms (n=204 households, 3 per cluster).

• Primary outcome, on page 11, in the paragraph about population for outcome monitoring, the authors indicate that "all children aged 1-9 years in the "yolks"… will be examined and sampled" and then on page 12 starting lines 7-8, the authors indicate that a random sample of 60 children will be taken at the primary outcome time point. Could the authors reword one or both of these sections to make them consistent? The way it currently reads, I thought the first section implied that every child would be monitored for outcomes ("examined and sampled" – not exactly sure whether sampling here refers to sampling to be included in the monitoring population or having samples taken). It sounds like repeated cross-sectional random sampling is in fact used, presumably at all time points? Or is this only used at the primary time point and all children are taken at all other time points?

Repeated cross-sectional random sampling is used at each of the monitoring survey time points. For logistic reasons we did sample all yolk household children in the baseline survey. The sample size calculation for the primary endpoint comparison requires a sample of 60 children aged 1-9 years randomly sampled from yolk households (Lines 379-381 and 405), whereby we ensured that each cluster achieved at least this number.

• Sample size, page 12 – could the authors provide references for the assumptions about Ct prevalence in the comparison arm and the expected ICC?

The expected prevalence of Ct in 1-9 year olds in the comparison arm was estimated from Ramadhani et al PLoS Negl Trop Dis 2016 https://doi.org/10.1371/journal.pntd.0005080). Based on this and the assumption 95% of the cluster level prevalence data were expected to lie in the range between 0 and 14% this gives an approximate coefficient of variation, k, of 0.7 (k=sd/prevalence), which converted to an ICC is approximately 0.025 (icc= [k^2]*prev/[1-prev]). We have amended the text and included the reference above (Lines 434-435 and 677-679). These assumptions were born out in the baseline data with balance between arms.

• Analysis – would the authors provide a reference and/or justification for their choice of approach to estimate risk difference? I'm curious about the rationale for the two-stage approach as opposed to using a risk difference model in one step (such as a GLM with a binomial outcome and identity link)? Now that I've read further, in the secondary outcomes section, the Hayes & Moulton reference is given – if this is referencing the same method, could the authors move this sentence to the primary outcome section? Also, is this all estimated at the cluster level, or is this estimated at the individual level with some method used to account for clustering (and if so, what method)?

All outcomes will be estimated as cluster-level summary statistics. Our analytical approach is considered to be a robust and standard approach for cluster randomised trials. We have moved the referenced statement to the primary outcome section as requested, for clarity (Lines 467-468). We agree that the method proposed by the reviewer would also be appropriate and this was considered originally. We concluded that either method would be appropriate, but that our chosen approach may be more robust if there were clusters with a prevalence of zero. We will conduct a sensitivity analysis using the method the reviewer describes to ensure that results are not dependent upon the method chosen.

• Figure 2 – the image appears distorted or stretched for me.

Thank you, this has been adjusted.

Reviewer: 2
Dr. Tao Xiong, West China Second University Hospital, Sichuan University
Comments to the Author:
This manuscript is a protocol for a cluster randomised controlled trial of enhanced antibiotic or/and enhanced F&E on trachoma control. This study could help in decision-making for trachoma programme managers. The method is detailed and the design is strict, but there are some concerns. **Thank you for your considered review of our protocol manuscript. We have provided our response to your queries below (in bold).**

1. Based on the trial status provided in the manuscript, it seems that lots of works have been done. The authors stated "Further reinforcement events for the 'Enhanced F&E' interventions will be held in 2023-2024. The primary outcome survey will be conducted in May 2024, and thereafter data collection will end". Were these works complete as expected? If so, these works should state in the past tense. Importantly, was the data collection finished? If it was complete, the protocol will not suitable for publication.

The tense has been amended as appropriate throughout the manuscript. The primary outcome survey was recently completed in August 2024. Field data collection continued until the end of August 2024, with laboratory work towards the primary outcome currently on-going. This protocol manuscript was submitted to BMJ Open on 19th January 2024 and was returned to us to respond to reviewer comments on 17th August 2024. We hope that this delay on behalf of the journal will be taken into account in order to publish this protocol for an important trial towards the elimination of a globally important infectious eye disease.

2. Based on the manuscript, three annual rounds of antibiotic MDA were delivered. Assessments will be conducted at 2 month, 12 months, 14 months, 24 months, 26 months, and 36 months after the first baseline assessment. Was the first year of MDA conducted during the baseline assessment time? Moreover, it is not clear whether the second and third year of annual MDA were delivered prior to or after the assessments at 12 months and 24 months.

The assessments were not done at the time of MDA, rather prior to or after an MDA to evaluate the impact of the intervention over time. Similarly, the baseline assessment was conducted prior to, not at the same time, as MDA that year. The 12-month assessment (one year after the first MDA), the 24-month assessment (one year after the second MDA, and two after the first) and the 36-month assessment (one year after the third, and three after the first) were conducted prior to the MDA delivery that year (with the exception of the 36-month assessment, which was the study endpoint, after which no further MDA was given. The Trial Timeline schematic in Supplementary 4 shows the relationship between MDA and the surveys (clinical assessments) and demonstrates their separation.

3. Three assessments was conducted at 2 months, 14 months, and 26 months (2 months after annual MDA in standard antibiotic arms and 1.5 months after annual MDA in enhanced antibiotic arms). There is a concern about whether 2 months is too short to compare the effectiveness between standard and enhanced MDA. Given that the prevalence of ocular Ct could drop rapidly after antibiotic MDA, and it could rebound as time goes by, the effectiveness of enhanced antibiotic could be overestimated if the assessment times are too close to antibiotic distribution time.

We conducted assessments two months after each MDA in addition to the 12 and 24-month time points (prior to the MDA) to provide data on the effectiveness of MDA during the trial to inform estimates of the likelihood of clearance of infection by one or two doses of azithromycin. In all arms it was 2 months following, rather than 1.5, as the surveys were rolling across the study clusters such that the assessments would occur cross-sectionally in the appropriate window after each MDA had been completed. The reviewer is correct to say that these time points would be too early to demonstrate overall effectiveness of MDA, which is the basis for the final 36-month time point, which is one year following the final round of MDA in the study communities.

4. This study is conducted in Oromia, Ethiopia. MDA with azithromycin has been given annually in this region since 2016. Prior to the trial intervention MDA had not been given since 2018. How about the baseline prevalence of ocular Ct or active trachoma in this region. How the previous MDA perform on controlling trachoma in this region? It is better to show some local epidemiologic data about the prevalence of trachoma in the introduction part.

There were no population-based ocular Ct data available in this region prior to this trial. Rationale for choosing the study site, and for the trial itself is given in Lines 159-161, where we indicate that Global Trachoma Mapping Project data for the Oromia region allowed us to purposively select kebeles with a high prevalence of TF in children aged 1-9 years for inclusion in the trial. Subsequent trial baseline TF and Ct prevalence was in line with these assumptions and was used to ensure balance across study arms.

5. Why children under two years of age are excluded for the second dose of azithromycin in the enhanced antibiotic arms instead of under six months of age.

The Ethiopian Federal Drug Administration and National Research Ethics Committee in Ethiopia did not allow us to give a second dose of azithromycin to children under the age of 2 years, despite the availability of safety and tolerability data, and therefore we were unable to administer the intervention to this age group. We have alluded to this in Lines 185-187. Our primary outcome population will include children aged 1-9 years and we will discuss this in our interpretation of these data, including as a potential limitation of the trial.

6. Comparison will be conducted between the stronger SAFE arm (arm 4) and the standard SAFE arm (arm 1). Whether the comparisons between arm 2 and arm 1 or between arm 3 and 1 will be performed.

The comparison between standard SAFE (arm 1) and Stronger SAFE (arm 4) is the primary (endpoint) comparison. We will also compare arm 2 (enhanced MDA only) with arm 1 and arm 3 (enhanced F&E) with arm 1. We will additionally assess the effectiveness of each

intervention, using a factorial-style approach (arms 1&2 vs. arms 3&4, and arms 1&3 vs. arms 2&4). These comparisons will use the same two-stage approach as used for the primary comparison, although for the F&E intervention comparison we will use the matched pair approach, additionally adjusting for the matched pair in stage 1. For the factorial-style analysis we will additionally adjust each intervention for the other (i.e. the effect estimate of enhanced antibiotic will be adjusted for F&E type, and vice versa). This has been included in the manuscript in Lines 472-477.

Reviewer: 1
Competing interests of Reviewer: None

Reviewer: 2
Competing interests of Reviewer: none

VERSION 2 - REVIEW

**Reviewer**	**1**
**Name**	**O'Brien, Kieran**
**Affiliation**	**University of California, San Francisco, Francis I. Proctor Foundation**
**Date**	**31-Oct-2024**
**COI**	

I would like to thank the authors for their detailed attention and responses to the editorial and reviewer comments. All of my initial questions and concerns have been adequately addressed, and I recommend this manuscript be accepted for publication.

**Reviewer**	**2**
**Name**	**Xiong, Tao**
**Affiliation**	**West China Second University Hospital, Sichuan University, Department of Pediatrics**
**Date**	**27-Oct-2024**
**COI**	

Thank you for your responses. My questions have been answered and I recommend this manuscript for publication.
